# Breast cancer mortality in 500 000 women with early invasive breast cancer diagnosed in England, 1993-2015: population based observational cohort study

**DOI:** 10.1136/bmj-2022-074684

**Published:** 2023-06-13

**Authors:** Carolyn Taylor, Paul McGale, Jake Probert, John Broggio, Jackie Charman, Sarah C Darby, Amanda J Kerr, Timothy Whelan, David J Cutter, Gurdeep Mannu, David Dodwell

**Affiliations:** 1 Nuffield Department of Population Health, University of Oxford, Oxford, UK; 2 Oxford University Hospitals, Oxford, UK; 3 National Disease Registration Service (NDRS), NHS England, Birmingham, UK; 4 Department of Oncology, McMaster University and Juravinski Cancer Centre, Hamilton, ON Canada

## Abstract

**Objectives:**

To describe long term breast cancer mortality among women with a diagnosis of breast cancer in the past and estimate absolute breast cancer mortality risks for groups of patients with a recent diagnosis.

**Design:**

Population based observational cohort study.

**Setting:**

Routinely collected data from the National Cancer Registration and Analysis Service.

**Participants:**

All 512 447 women registered with early invasive breast cancer (involving only breast and possibly axillary nodes) in England during January 1993 to December 2015, with follow-up to December 2020.

**Main outcome measures:**

Annual breast cancer mortality rates and cumulative risks by time since diagnosis, calendar period of diagnosis, and nine characteristics of patients and tumours.

**Results:**

For women with a diagnosis made within each of the calendar periods 1993-99, 2000-04, 2005-09, and 2010-15, the crude annual breast cancer mortality rate was highest during the five years after diagnosis and then declined. For any given time since diagnosis, crude annual breast cancer mortality rates and risks decreased with increasing calendar period. Crude five year breast cancer mortality risk was 14.4% (95% confidence interval 14.2% to 14.6%) for women with a diagnosis made during 1993-99 and 4.9% (4.8% to 5.0%) for women with a diagnosis made during 2010-15. Adjusted annual breast cancer mortality rates also decreased with increasing calendar period in nearly every patient group, by a factor of about three in oestrogen receptor positive disease and about two in oestrogen receptor negative disease. Considering just the women with a diagnosis made during 2010-15, cumulative five year breast cancer mortality risk varied substantially between women with different characteristics: it was <3% for 62.8% (96 085/153 006) of women but ≥20% for 4.6% (6962/153 006) of women.

**Conclusions:**

These five year breast cancer mortality risks for patients with a recent diagnosis may be used to estimate breast cancer mortality risks for patients today. The prognosis for women with early invasive breast cancer has improved substantially since the 1990s. Most can expect to become long term cancer survivors, although for a few the risk remains appreciable.

**Figure:**
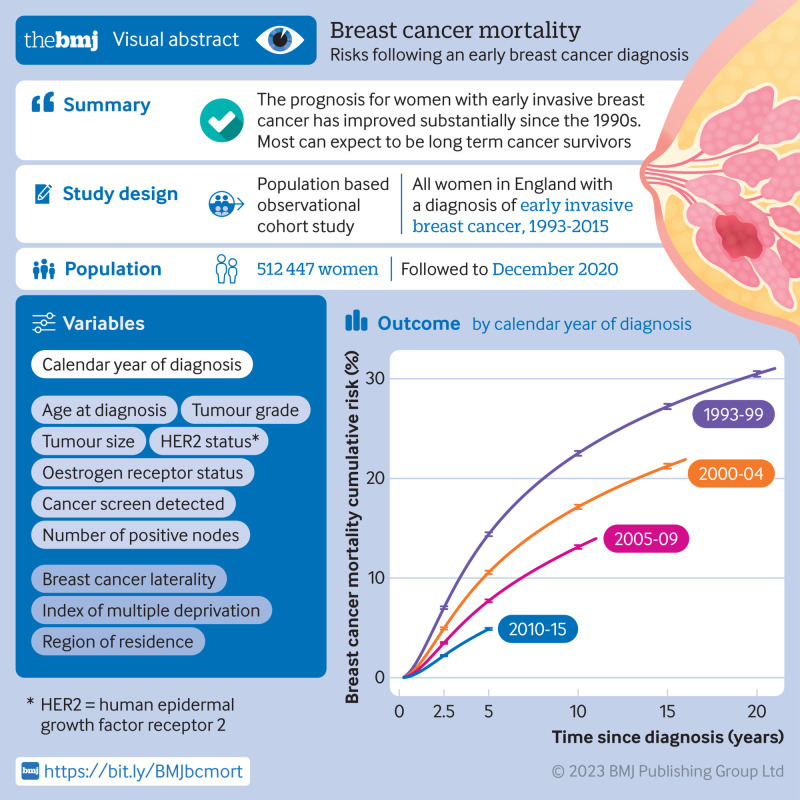


## Introduction

Worldwide, more than 2 million patients receive a diagnosis of invasive breast cancer each year.^1^ For most, it is their first cancer. Most have early stage disease and receive surgery as their first treatment. Outcomes following treatment for early invasive breast cancer differ substantially across different countries and across patients with different characteristics within a country. Patients with breast cancer and the clinicians who treat them need estimates of their likely prognosis to inform treatment decisions, follow-up, and prediction of event rates for groups of patients in clinical trials. These estimates require large scale, population based studies that consider the effects of multiple patient related and tumour related factors on breast cancer mortality. Survival has improved, and detailed breast cancer mortality estimates based on patients treated recently are not available. These are needed to enable clinicians to estimate prognosis for patients treated today by using characteristics such as age, tumour size, nodal status, tumour grade, receptor status, and screening status.

Most population based studies in women with early invasive breast cancer either consider just a few specific determinants of breast cancer mortality or consider selected groups of women with specific characteristics.^2^ ^3^ ^4^ ^5^ ^6^ ^7^ ^8^ ^9^ ^10^ ^11^ An additional limitation is that few of these studies have considered the extent to which breast cancer mortality has changed over the past few decades for women with early invasive breast cancer or the way in which it varies with time since diagnosis.

We conducted the first observational cohort study showing long term outcomes in all women registered in England with early invasive breast cancer as their first cancer and who were treated initially with surgery. We describe annual breast cancer mortality rates and cumulative breast cancer mortality risks in all women with a diagnosis made during 1993-2015, and the extent to which they vary with calendar period of diagnosis, time since diagnosis, whether the cancer was screen detected, and characteristics of patients and tumours. We then focus on women with a diagnosis made during 2010-15 to inform patients and clinicians about the likely absolute mortality risks for individual patients treated for breast cancer today, taking into account their tumour characteristics, including whether the cancer was screen detected. We did not aim to assess the effects of treatment, which would need data from randomised trials.^12^ Neither did we aim to quantify the effects of screening on changes in mortality.

## Methods

### Study population

The National Cancer Registration and Analysis Service (NCRAS), together with its preceding organisations, has registered all patients with a diagnosis of cancer in England for many decades.^13^ These registrations are routinely linked with other information at the level of the patient. For this study, all women registered in England during January 1993 to December 2015 with breast cancer as their first invasive cancer were identified and their data were de-personalised before release for analysis.

Early invasive breast cancer was defined as disease detected only in the breast or, in the case of women with node positive disease, the breast and axillary lymph nodes with no evidence of metastatic disease.^14^ Surgery was either breast conserving surgery or mastectomy.

Data included pathological staging (tumour size and number of positive axillary nodes in the surgical specimen), grade (low, medium, or high), and oestrogen receptor status (with oestrogen receptor positivity defined as >10% staining or Allred score 3-8). For women with a diagnosis made during 2010-15, human epidermal growth factor receptor 2 (HER2) status was also included. These variables are routinely used in the clinic to estimate prognosis,^15^ ^16^ to define groups of patients in clinical guidelines, to inform treatment decisions, and to assess eligibility for clinical trials. Other variables were calendar period of diagnosis, age at diagnosis, screening status, laterality, index of multiple deprivation, region of residence, and, where applicable, dates of emigration and death, together with causes of death, up to 31 December 2020.

Data were collated and checked before analysis; see supplementary text S1 and figure S1 for details. We received data on a total of 783 980 women (figure S2). We excluded women if they were aged <18 or ≥90 years at diagnosis (n=24 157), their registrations were based on a death certificate only or they had less than three months of follow-up (n=33 086), or their histology was not invasive breast cancer (n=10 798). We also excluded the 22 577 women with diagnoses of two simultaneous cancers, as patterns of breast cancer mortality among these women may differ from those of women with just one cancer. These women comprised those with a second primary non-breast cancer within three months of registration and women with bilateral breast cancer or a contralateral breast cancer within three months of registration, who would each have two sets of breast tumour related factors. Analysing data for them would require a different method from that appropriate for women with unilateral breast cancer and just one set of tumour related factors. In addition, we excluded 138 911 women because they had likely metastatic disease. A further 42 004 were recorded as receiving neoadjuvant therapy (7.6% (42 004/554 451) of women overall; 8.1% (19 996/248 176) of women with a diagnosis made in 1993-2004 and 7.2% (22 008/306 275) with a diagnosis made in 2005-15). We excluded them as the characteristics of their tumours were recorded before the neoadjuvant therapy was given and are therefore based on clinical staging. This is in contrast to other women, for whom pathological staging was recorded.

### Statistical analysis

Women were included in the study from three months after diagnosis and removed on the earliest of date of death, emigration, 95th birthday, or end of follow-up. We considered calendar period in five year categories, except that we included women with diagnoses made during 1993-94 with the 1995-99 period and included women with diagnoses made during 2015 with the 2010-14 period. Within each calendar period category, we wanted the maximum possible length of follow-up to be the same for all women. Therefore, unless a woman had previously died, emigrated, or reached her 95th birthday, we removed her from the study at 21 years from diagnosis if she had a diagnosis made during 1993-99, 16 years if she had a diagnosis made during 2000-04, 11 years if she had a diagnosis made during 2005-09, and five years if she had a diagnosis made during 2010-15 (text S2).

Information was missing for some women on tumour size, number of positive nodes, tumour grade, oestrogen receptor status, laterality, and (applicable to the period 2010-15) HER2 status. Therefore, to avoid the loss of precision and possible bias that would be caused by omitting these women from the analysis or by creating separate categories for missing values, we used multiple imputation of missing values in analyses involving these characteristics (see text S2, table S1, and table S2 for details of missing data).

We did the main analyses in four steps. In the first step, we estimated crude annual breast cancer mortality rates by time since diagnosis assuming a Poisson distribution. We then smoothed these annual rates (see figure S3 for a comparison of raw and smoothed rates) and estimated cumulative risks by time since diagnosis from the smoothed rates. We made these calculations separately for categories of calendar period of diagnosis, screening status, and oestrogen receptor status and also for combinations of these three characteristics.

In the second step, which we did separately for women with oestrogen receptor positive and oestrogen receptor negative disease, we calculated adjusted annual mortality rates according to each available characteristic by Poisson regression, with time since diagnosis and all other available characteristics as categorical variables using categories defined as shown in table 1. The third step examined the extent to which both absolute and proportional changes in the mortality rate with increasing calendar period of diagnosis varied across the different characteristics. We found substantial variability on both absolute and proportional scales. This meant that the data could not be summarised in any simple quantitative model. Therefore, in the fourth step, we did analyses of just the women with diagnoses made during 2010-15 to describe breast cancer mortality rates for groups of patients with a recent diagnosis. We calculated adjusted annual mortality rates for each available characteristic by Poisson regression, as in the second step above. This was followed by tests for pairwise interactions between the characteristics. Lastly, we calculated cumulative five year risks separately for women with different combinations of the characteristics HER2 status, age at diagnosis, screening status, number of positive nodes, and tumour size and grade.

**Table 1:** Characteristics of 512 447 women given a diagnosis of early breast cancer in England during 1993-2015.

Characteristic	No (%) women by calendar period of diagnosis				
	1993-99 (n=113 354; 16 193/year)	2000-04 (n=114 826; 22 965/year)	2005-09 (n=127 929; 25 586/year)	2010-15 (n=156 338; 26 056/year)	All years (n=512 447; 22 280/year)
Age at diagnosis, years*:					
18-39	7852 (7)	7002 (6)	6552 (5)	6717 (4)	28 123 (5)
40-49	21 718 (19)	19 313 (17)	22 369 (17)	27 908 (18)	91 308 (18)
50-64	47 465 (42)	50 761 (44)	53 741 (42)	62 074 (40)	214 041 (42)
65-70	13 385 (12)	14 399 (13)	20 207 (16)	27 419 (18)	75 410 (15)
71-79	16 988 (15)	16 703 (15)	17 122 (13)	21 972 (14)	72 785 (14)
80-89	5946 (5)	6648 (6)	7938 (6)	10 248 (7)	30 780 (6)
Cancer screen detected:					
Eligible—screen detected	14 981 (13)	20 190 (18)	41 272 (32)	51 797 (33)	128 240 (25)
Eligible—not screen detected	32 484 (29)	30 571 (27)	32 676 (26)	37 696 (24)	133 427 (26)
Not eligible for screening	65 889 (58)	64 065 (56)	53 981 (42)	66 845 (43)	250 780 (49)
Tumour size, mm†:					
1-20	66 733 (59)	66 811 (58)	73 659 (58)	93 336 (60)	300 539 (59)
21-50	42 375 (37)	43 333 (38)	48 315 (38)	56 238 (36)	190 261 (37)
>50	4246 (4)	4682 (4)	5955 (5)	6764 (4)	21 647 (4)
No of positive nodes†:					
0	53 773 (47)	63 095 (55)	69 693 (54)	101 898 (65)	288 459 (56)
1-3	41 169 (36)	34 458 (30)	39 543 (31)	40 494 (26)	155 664 (30)
4-9	13 498 (12)	12 034 (10)	12 591 (10)	9601 (6)	47 724 (9)
≥10	4914 (4)	5239 (5)	6102 (5)	4345 (3)	20 600 (4)
Tumour grade†:					
Low	25 257 (22)	23 427 (20)	22 036 (17)	26 738 (17)	97 458 (19)
Medium	50 361 (44)	53 362 (46)	61 618 (48)	79 842 (51)	245 183 (48)
High	37 736 (33)	38 037 (33)	44 275 (35)	49 758 (32)	169 806 (33)
Oestrogen receptor status†:					
Positive	86 965 (77)	93 781 (82)	106 485 (83)	134 398 (86)	421 629 (82)
Negative	26 389 (23)	21 045 (18)	21 444 (17)	21 940 (14)	90 818 (18)
HER2 status†:					
Negative	-	-	-	135 875 (87)	135 875 (27)
Positive	-	-	-	20 463 (13)	20 463 (4)
Before 2010	113 354 (100)	114 826 (100)	127 929 (100)	-	356 109 (69)
Breast cancer laterality†:					
Left	58 708 (52)	59 093 (51)	65 830 (51)	80 276 (51)	263 907 (51)
Right	54 646 (48)	55 733 (49)	62 099 (49)	76 062 (49)	248 540 (49)
Index of multiple deprivation:					
<20% (least deprived)	25 386 (22)	26 034 (23)	29 601 (23)	37 027 (24)	118 048 (23)
20-39%	25 389 (22)	26 205 (23)	29 360 (23)	36 112 (23)	117 066 (23)
40-59%	23 854 (21)	24 143 (21)	26 987 (21)	32 825 (21)	107 809 (21)
60-79%	21 148 (19)	21 131 (18)	23 273 (18)	27 985 (18)	93 537 (18)
≥80% (most deprived)	17 577 (16)	17 313 (15)	18 708 (15)	22 389 (14)	75 987 (15)
Region of residence‡:					
Eastern	12 195 (11)	12 734 (11)	13 623 (11)	18 238 (12)	56 790 (11)
North West	16 270 (14)	15 936 (14)	17 514 (14)	20 280 (13)	70 000 (14)
Northern and Yorkshire	14 095 (12)	15 521 (14)	17 039 (13)	20 275 (13)	66 930 (13)
Oxford	7776 (7)	6643 (6)	7149 (6)	9374 (6)	30 942 (6)
South West	18 712 (17)	17 589 (15)	20 222 (16)	24 520 (16)	81 043 (16)
Thames	24 723 (22)	23 093 (20)	27 231 (21)	32 564 (21)	107 611 (21)
Trent	5174 (5)	10 455 (9)	11 218 (9)	15 109 (10)	41 956 (8)
West Midlands	14 409 (13)	12 855 (11)	13 933 (11)	15 978 (10)	57 175 (11)
End of follow-up (years from diagnosis):					
≤1	3135 (3)	2130 (2)	1688 (1)	1308 (1)	8261 (2)
1-2	5166 (5)	3864 (3)	3190 (2)	2695 (2)	14 915 (3)
2-3	5216 (5)	4057 (4)	3579 (3)	3122 (2)	15 974 (3)
3-4	5064 (4)	4009 (3)	3778 (3)	3458 (2)	16 309 (3)
4-5	3959 (3)	3573 (3)	3200 (3)	145 755 (93)	156 487 (31)
5-10	16 231 (14)	14 874 (13)	15 735 (12)	-	46 840 (9)
10-15	12 784 (11)	13 497 (12)	96 759 (76)	-	123 040 (24)
15-20	11 229 (10)	68 822 (60)	-	-	80 051 (16)
20-21	50 570 (45)	-	-	-	50 570 (10)

HER2=human epidermal growth factor receptor 2 (data available only for period 2010-15).

* Categories reflect eligibility for breast cancer screening programme (ie, 50-64 years for all calendar periods of diagnosis and 65-70 years from 2005). See table S3 for separate values according to screening status.

† Some values were unknown in original data; values for these characteristics have been estimated using multiple imputation (see text S2 for details).

‡ Based on former cancer registry regions.

We did analyses for all cause mortality along similar lines to those for breast cancer mortality. We used Stata version 17 for all calculations. Further details are in text S2 and tables S1 and S2.

### Patient and public involvement

Two patients representing the organisation Independent Cancer Patients’ Voice were involved as research partners. They helped to develop the research questions. They identified the need for up-to-date information on outcomes after breast cancer diagnosis to inform prognosis for current and future patients. They advised on which analyses were important to patients with breast cancer and provided input to the interpretation. They reviewed and commented on the main findings in the manuscript. Their input was provided in face-to-face meetings, in teleconferences, and via email. They have agreed to help with dissemination of the findings.

## Results

### Characteristics of study population

A total of 512 447 women with early invasive breast cancer were included in the study. Breast surgery was either breast conserving surgery (60%; 307 714 women) or mastectomy (40%; 204 733 women). For axillary surgery, 154 583 (30%) had axillary dissection only, 218 313 (43%) had sentinel lymph node biopsy only, 79 559 (16%) had both, and 59 992 (12%) had no record of either. The number of women who received a diagnosis of early breast cancer each year increased over the study period from an average of 16 193 during 1993-99 to 26 056 during 2010-15 (table 1). Before 2005 only women aged 50-64 years were eligible for screening and the overall percentage of women whose cancers were screen detected was 13% (14 981/113 354) in 1993-99 and 18% (20 190/114 826) in 2000-04. These percentages increased substantially when screening was also offered to women aged 65-70 years, to 32% (41 272/127 929) during 2005-09 and 33% (51 797/156 338) during 2010-15. Overall, nearly half the cancers among women in age groups eligible for screening were screen detected.

The percentage of women with node negative disease increased from 47% (53 773/113 354) in 1993-99 to 65% (101 898/156 338) in 2010-15, and the percentage with tumours categorised as oestrogen receptor positive increased from 77% (86 965/113 354) in 1993-99 to 86% (134 398/156 338) in 2010-15. By contrast, the percentage of women with a diagnosis of low grade disease decreased from 22% (25 257/113 354) in 1993-99 to 17% (26 738/156 338) in 2010-15.

The geographical spread reflected the population of England, with more than 30 000 women from each region. Among the 512 447 women, duration of follow-up from time of diagnosis was five years or less for 211 946 (41%) women, five to 10 years for 46 840 (9%) women, 10-15 years for 123 040 (24%) women, 15-20 years for 80 051 (16%) women, and more than 20 years for 50 570 (10%) women (table 1). By end of follow-up, 77 975 (15%) of the women in the study had died from breast cancer and 155 895 (30%) from any cause.

### All women: crude breast cancer mortality

**Fig 1:**
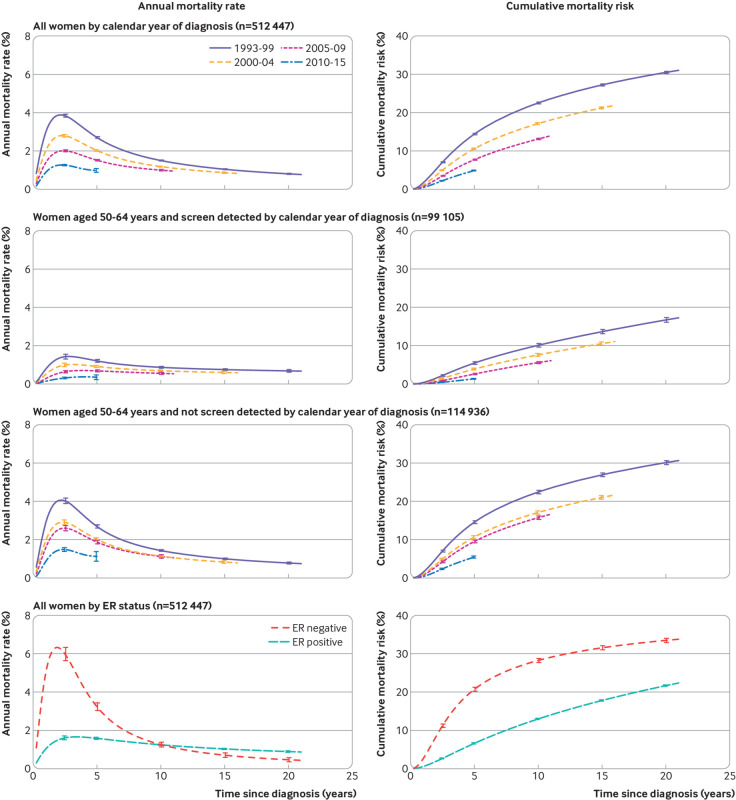
Crude annual breast cancer mortality rates and cumulative breast cancer mortality risks in 512 447 women with early breast cancer by time since diagnosis according to calendar period of diagnosis: for all women (top panel), for women aged 50-64 years (who would all have been eligible for screening) according to whether their cancer was screen detected (middle panels), and according to oestrogen receptor (ER) status (bottom panel). Vertical lines are 95% confidence intervals. Numbers of women at risk, numbers of deaths from breast cancer, annual rates, and cumulative mortality risks are given in tables S4 to S6. Further analyses of women by ER status are in figures S4 and S5. Analyses of non-breast cancer mortality and all cause mortality are in figures S23 and S24. Analyses of women with metastatic as well as early breast cancer, and including women who received neoadjuvant therapy, are in figure S25

To assess the pattern of breast cancer mortality by time since diagnosis in each calendar period, we analysed data on women with a diagnosis made in each calendar period separately. Regardless of calendar period of diagnosis, annual breast cancer mortality rates increased during the two years following diagnosis, peaked during the third year, and then declined (fig 1, top left). For any given time since diagnosis, cumulative risk of breast cancer mortality decreased progressively in successive calendar periods (fig 1, top right). Five year cumulative mortality risk was 14.4% (95% confidence interval 14.2% to 14.6%) for women with a diagnosis made during 1993-99 and reduced progressively with increasing calendar period to 4.9% (4.8% to 5.0%) for women with a diagnosis made during 2010-15. We observed corresponding decreases in mortality risk when we repeated the analyses including deaths from other causes as well as breast cancer (figures S23 and S24) or including all women with a diagnosis of breast cancer, even if they had metastatic disease at diagnosis or were recorded as receiving neoadjuvant therapy (figure S25).

To assess whether the decreases in breast cancer mortality among women given a diagnosis of early invasive breast cancer resulted solely from increases in screening, we did separate analyses for women with a diagnosis made in different calendar periods and with different screening status for women aged 50-64 years at diagnosis, all of whom would have been eligible for screening throughout the period included in the study (fig 1, middle panels). For patients with a diagnosis of either screen detected or non-screen detected cancers, annual breast cancer mortality rates and cumulative breast cancer mortality risks showed similar patterns by calendar period of diagnosis to those for all women, but with lower values in women with screen detected compared with non-screen detected cancers.

To assess differences in breast cancer mortality according to oestrogen receptor status, we did separate analyses for women with oestrogen receptor positive and oestrogen receptor negative disease. Considering all calendar periods together, annual breast cancer mortality rates peaked during the third year after diagnosis in both groups but at a much higher rate in oestrogen receptor negative disease (fig 1, bottom panel). Beyond three years, annual breast cancer mortality rates decreased in both groups but more rapidly in oestrogen receptor negative than oestrogen receptor positive disease so that, beyond 10 years, the annual breast cancer mortality rate was higher in oestrogen receptor positive than oestrogen receptor negative disease. In both groups, deaths from breast cancer continued to occur until the end of follow-up, irrespective of screening status (figure S4).

### All women: adjusted breast cancer mortality

We analysed data on all 512 447 women with a diagnosis made during 1993-2015 to assess the independent effects of calendar period of diagnosis and eight other characteristics of patients and tumours on breast cancer mortality in oestrogen receptor positive and oestrogen receptor negative disease. In these analyses, we calculated annual breast cancer mortality rates separately for each characteristic by oestrogen receptor status after adjusting for the other eight characteristics and for time since diagnosis.

**Fig 2:**
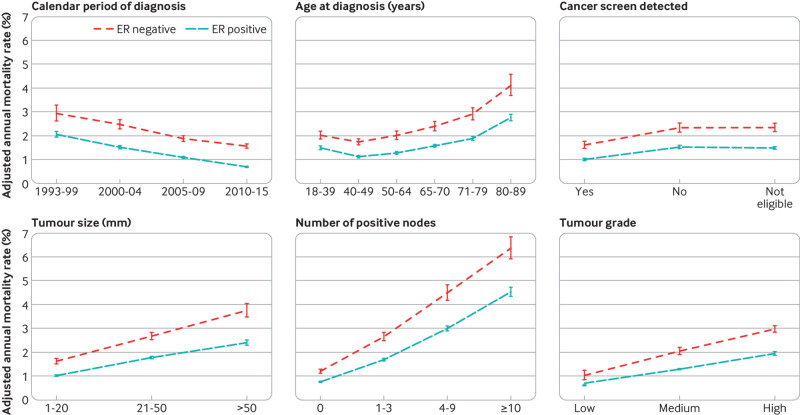
Adjusted annual breast cancer mortality rates in 512 447 women with early breast cancer with oestrogen receptor (ER) positive or ER negative disease, by various characteristics. For each characteristic, rates are adjusted for all other characteristics shown and also for time since diagnosis, breast cancer laterality, index of multiple deprivation, and region of residence. Vertical lines are 95% confidence intervals. Results for breast cancer laterality, index of multiple deprivation, and region of residence are in figure S6. Further details are in figure S7, and separate rates for years 0-5, 5-15, and ≥15 since diagnosis are in figures S8-S10. Analyses of all cause mortality are in figures S26-S28

The adjusted annual breast cancer mortality rate declined steadily with calendar period of diagnosis in both oestrogen receptor positive and oestrogen receptor negative disease, but rates were higher in oestrogen receptor negative disease (fig 2, top left). For every other characteristic, the adjusted annual breast cancer mortality rates also followed a similar pattern in oestrogen receptor positive and oestrogen receptor negative disease but with higher rates in oestrogen receptor negative disease (fig 2; figures S6 and S7). They were lowest for women in their 40s at diagnosis and highest for women in their 80s at diagnosis, and they were lower in women with screen detected cancer than in women with non-screen detected cancer or in women who were not eligible for screening. Tumour size, number of nodes, and tumour grade all had substantial independent effects, whereas the effects of cancer laterality, deprivation, and region of residence were smaller (figure S6). See figures S26-S28 for analyses of all cause mortality.

The variation in the adjusted breast cancer mortality rates for each characteristic considered was greatest in the first five years after diagnosis, but the effects of age at diagnosis, screening status, tumour size, number of positive nodes, and tumour grade were all still strong five to 15 years after diagnosis, and the effects of tumour size, number of nodes, and, in oestrogen receptor positive disease, age at diagnosis were still clearly present at ≥15 years (figures S8-S10).

**Fig 3:**
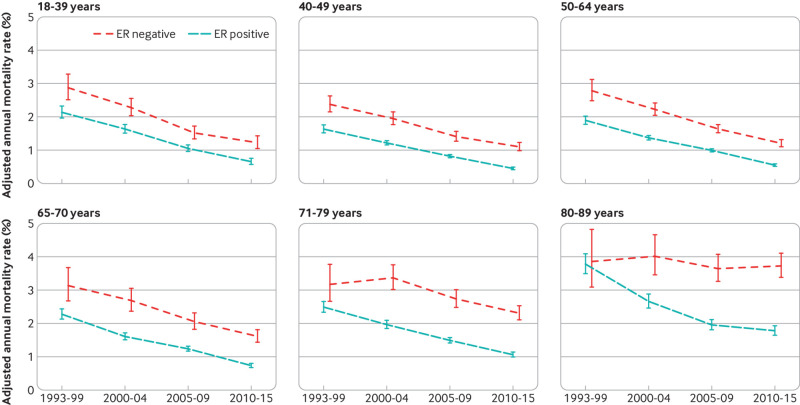
Adjusted annual breast cancer mortality rates in 512 447 women with early breast cancer with oestrogen receptor (ER) positive or ER negative disease by calendar period of diagnosis, according to age at diagnosis. Rates are adjusted for whether cancer was screen detected, tumour size, number of positive nodes, tumour grade, time since diagnosis, breast cancer laterality, index of multiple deprivation, and region of residence. Vertical lines are 95% confidence intervals. Further details are in figure S13. Results including only five years of follow-up (so that all age groups have the same length of follow-up) are in figure S11. Results for breast cancer laterality, index of multiple deprivation and region of residence are in figure S12. Results for all cause mortality are in figures S29 and S30

To examine whether the decreases in breast cancer mortality with more recent calendar period (fig 2, top left) took place to the same extent among all groups of women, we calculated adjusted annual breast cancer mortality rates by oestrogen receptor status and calendar period of diagnosis for women with different characteristics. The breast cancer mortality rate decreased substantially between 1993-99 and 2010-15 for all ages at diagnosis in women with oestrogen receptor positive disease and for most ages in women with oestrogen receptor negative disease, apart from those aged 80-89 at diagnosis, for whom we saw hardly any decrease (fig 3; figures S11 and S13). In younger women, breast cancer was the main cause of death so reductions in breast cancer mortality were similar to reductions in all cause mortality according to calendar period. In older women, all cause mortality was much higher than breast cancer mortality. Mortality reductions according to calendar period were greater for all cause mortality than for breast cancer mortality (figure S30).

For screening status, tumour size, number of positive nodes, tumour grade, breast cancer laterality, deprivation, and region of residence, the breast cancer mortality rate decreased substantially between 1993-99 and 2010-15 for every category in both oestrogen receptor positive and oestrogen receptor negative disease (fig 4; fig 5; fig 6; fig 7; figures S12 and S14-20). See figures S29 and S30 for analyses of all cause mortality.

**Fig 4:**
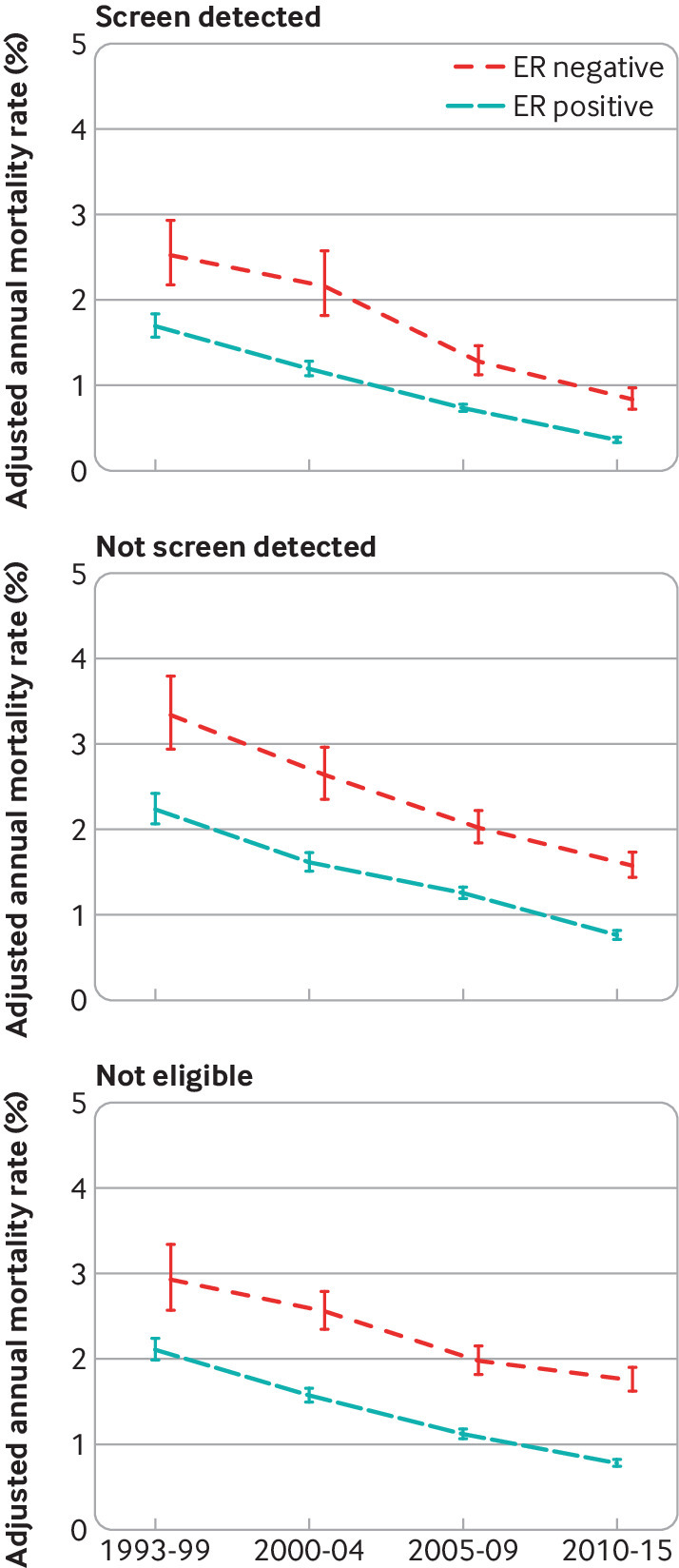
Adjusted annual breast cancer mortality rates in 512 447 women with early breast cancer with oestrogen receptor (ER) positive or ER negative disease by calendar period of diagnosis, according to whether their cancer was screen detected. Rates are adjusted for age at diagnosis, tumour size, number of positive nodes, tumour grade, time since diagnosis, breast cancer laterality, index of multiple deprivation, and region of residence. Vertical lines are 95% confidence intervals. Further details are in figure S14

**Fig 5:**
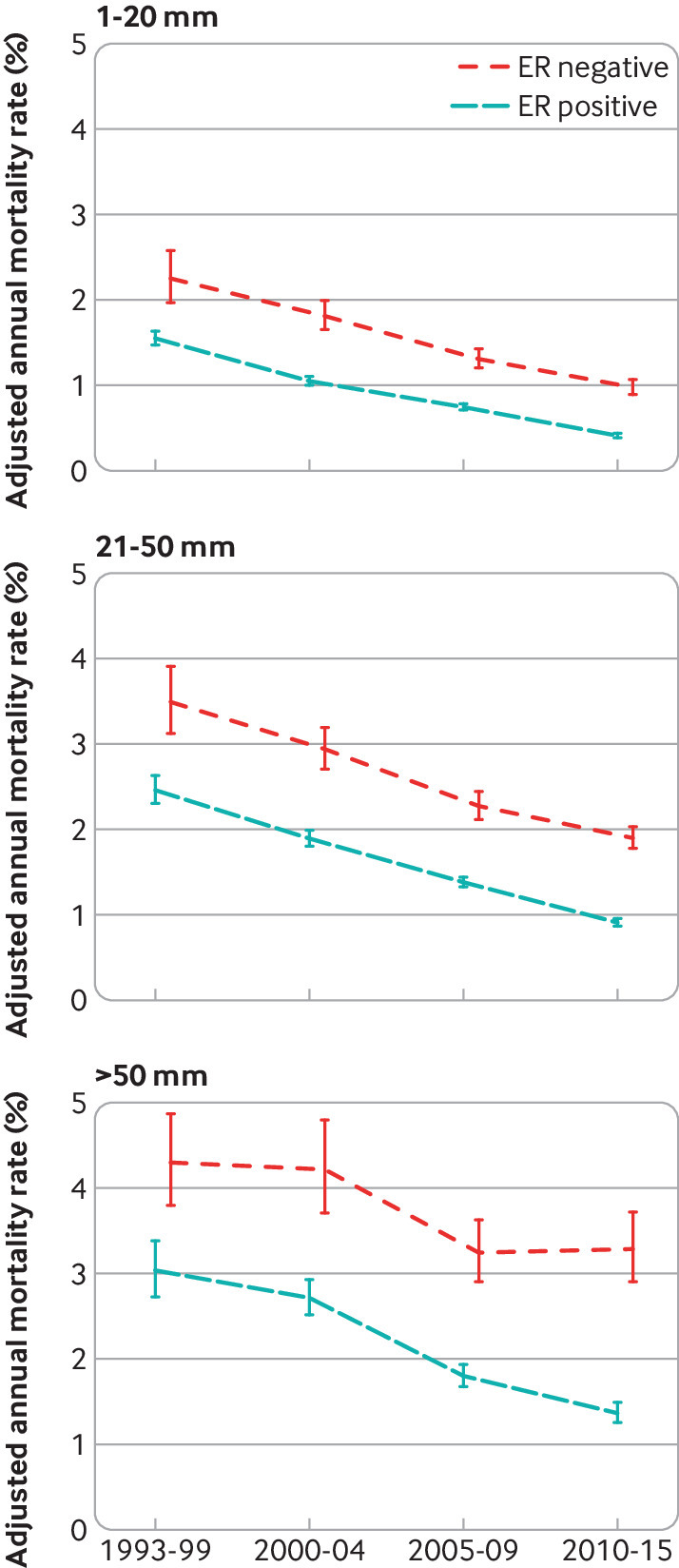
Adjusted annual breast cancer mortality rates in 512 447 women with early breast cancer with oestrogen receptor (ER) positive or ER negative disease by calendar period of diagnosis, according to tumour size. Rates are adjusted for age at diagnosis, whether cancer was screen detected, number of positive nodes, tumour grade, time since diagnosis, breast cancer laterality, index of multiple deprivation, and region of residence. Vertical lines are 95% confidence intervals. Further details are in figure S15

**Fig 6:**
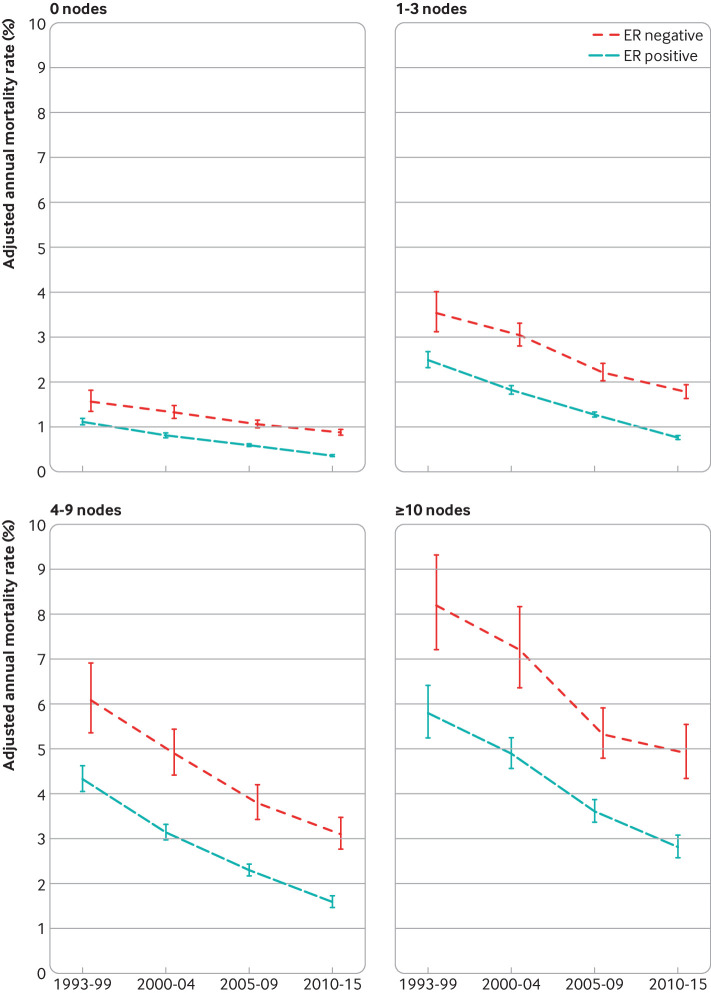
Adjusted annual breast cancer mortality rates in 512 447 women with early breast cancer with oestrogen receptor (ER) positive or ER negative disease by calendar period of diagnosis, according to number of positive nodes. Rates are adjusted for age at diagnosis, whether cancer was screen detected, tumour size, tumour grade, time since diagnosis, breast cancer laterality, index of multiple deprivation, and region of residence. Vertical lines are 95% confidence intervals. Further details are in figure S16

**Fig 7:**
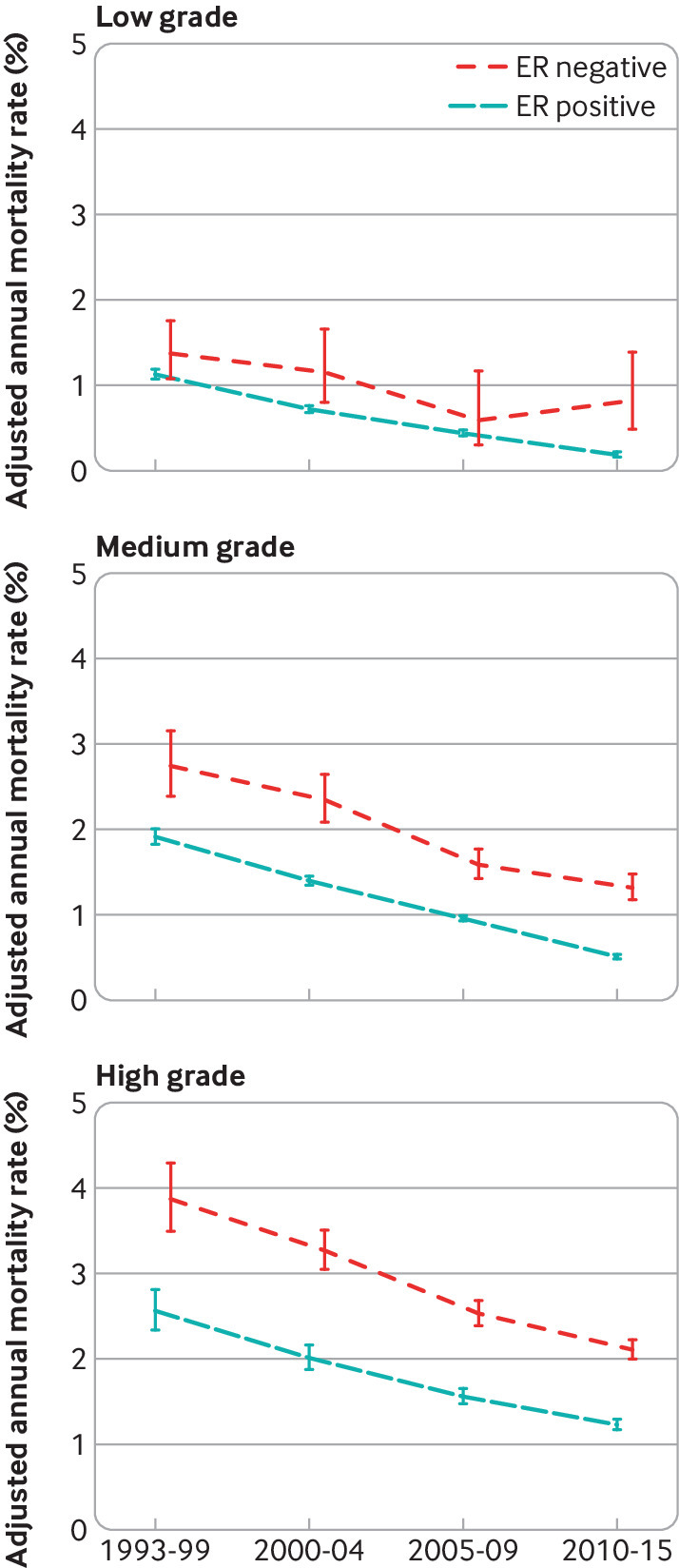
Adjusted annual breast cancer mortality rates in 512 447 women with early breast cancer with oestrogen receptor (ER) positive or ER negative disease by calendar period of diagnosis, according to tumour grade. Rates are adjusted for age at diagnosis, whether cancer was screen detected, tumour size, number of positive nodes, time since diagnosis, breast cancer laterality, index of multiple deprivation, and region of residence. Vertical lines are 95% confidence intervals. Further details are in figure S17

Although a decrease in breast cancer mortality rate in more recent calendar periods was apparent for all groups of women, apart from those aged 80-89 with oestrogen receptor negative disease, the absolute magnitude of the decrease varied substantially. For example, in oestrogen receptor positive disease, the adjusted annual rate decreased by 2.99 percentage points (from 5.80% (5.24% to 6.41%) in 1993-99 to 2.81% (2.57% to 3.08%) in 2010-15) among women with ≥10 positive nodes, but by only 0.75 percentage points (from 1.11% (1.05% to 1.19%) in 1993-99 to 0.36% (0.34% to 0.38%) in 2010-15) for women with no positive nodes (figure S16). Similarly, in oestrogen receptor negative disease, the adjusted rate decreased by 3.30 percentage points (from 8.20% (7.21% to 9.32%) in 1993-99 to 4.90% (4.34% to 5.54%) in 2010-15) among women with ≥10 positive nodes, but by only 0.68 percentage points (from 1.56% (1.35% to 1.81%) in 1993-99 to 0.88% (0.81% to 0.94%) in 2010-15) among women with no positive nodes. We also saw substantial variations in the absolute magnitude of the decrease across the different levels of many other characteristics (figures S13-15, S17-S20). The magnitude of the decreases with calendar period of diagnosis also varied between different levels of many characteristics when considered on a proportional scale (table S7). Therefore, as women who had a diagnosis made in the most recent calendar period are most relevant to women with diagnoses of breast cancer today, further analysis considered these women in greater detail.

### Women with diagnosis made 2010-15: breast cancer mortality

**Fig 8:**
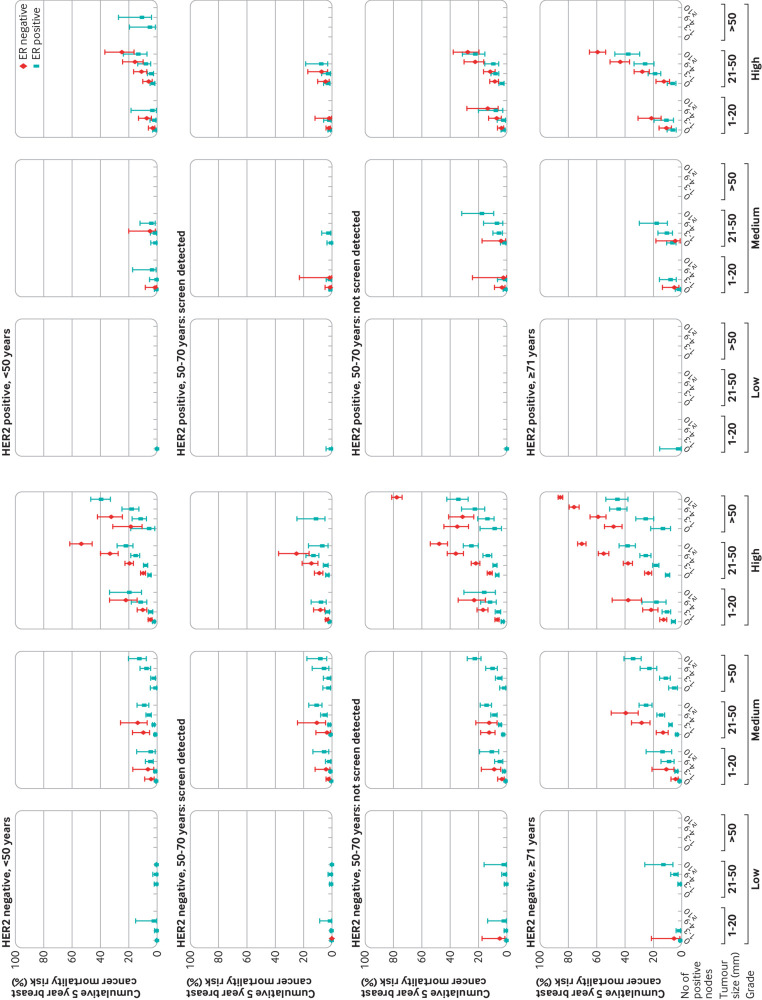
Cumulative five year breast cancer mortality risks in 156 338 women with early breast cancer diagnosed during 2010-15 by categories of tumour grade, tumour size and number of positive nodes in women with oestrogen receptor (ER) positive or ER negative disease. Figures are split by human epidermal growth factor receptor 2 (HER2) status, age and screening status. Vertical lines are 95% confidence intervals. Points are plotted for groups of women with data on ≥40 women and include 153 006/156 338 (97.9%) of women. Further details, including values of plotted points, are given in tables S9 and S10. Results on a square root scale are in figure S22. Results for all cause mortality are in figure S31

Among the 156 338 women with a diagnosis made during 2010-15, the adjusted annual breast cancer mortality rates varied substantially across categories of age, screening status, tumour size, number of positive nodes, and tumour grade in both oestrogen receptor positive and oestrogen receptor negative disease, whereas for HER2 status, which was available for women with diagnoses made in this time period, the adjusted annual mortality rate was lower in HER2 positive than HER2 negative disease in both oestrogen receptor positive and oestrogen receptor negative disease (figure S21). In addition, we found evidence that some of these factors did not combine in a multiplicative fashion in oestrogen receptor positive disease (table S8). Therefore, we calculated separate estimates of cumulative five year breast cancer mortality risk for all the 576 patient groups with different combinations of these factors (fig 8; table S9).

The largest such group was the 15 533 women aged 50-70 years at diagnosis with screen detected cancer that was HER2 negative, oestrogen receptor positive, medium grade, size 1-20 mm, and node negative (fig 8, second row, left side; table S9b). Women in this group had an average cumulative five year breast cancer mortality risk of 0.5% (0.4% to 0.7%). We found, however, substantial variability in the five year cumulative risk between the different combinations of characteristics. It took values <1% for 34.2% (52 257/153 006) of women, <3% for 62.8% (96 085/153 006), <5% for 73.7% (112 814/153 006), and <10% for 88.0% (134 655/153 006), whereas it was ≥20% for 4.6% (6962/153 006), ≥40% for 0.7% (1085/153 006), and ≥60% for 0.2% (238/153 006) of women (table S10).

## Discussion

We have identified all 512 447 women registered with early invasive breast cancer in England during 1993 to 2015 who were treated initially with surgery and followed them to 31 December 2020. We started by documenting associations between the breast cancer mortality rate and nine individual characteristics, providing insight into the prognostic value of each. We showed that the breast cancer mortality rate has decreased with calendar period of diagnosis over the period in our study. Although decreases occurred across nearly all groups of patients, the magnitude of the decrease in the mortality rate varied substantially between women with different characteristics, both on an absolute scale and on a proportional scale. This indicates that the associations between the variables we have studied and breast cancer mortality have been changing over time. Therefore, although the long term trends in women whose cancer was diagnosed during 1993-2009 are of some relevance for long term predictions, it is the mortality of women with breast cancer diagnosed in the most recent period that is the most relevant to women receiving a diagnosis today. Breast cancer mortality is highest during the five years following diagnosis and, although deaths from breast cancer will continue to occur beyond this, the risk during each subsequent five year period is likely to be lower than during the first five years. For these reasons, we presented the five year breast cancer mortality risks for women with a diagnosis made during 2010-15 with different combinations of age, screening status, oestrogen receptor status, number of positive nodes, and tumour size and grade. Focusing on 2010-15 also enabled us to consider HER2 status, which was not available for earlier calendar periods. Clinicians can use this information to estimate breast cancer mortality risks for patients today.

### Strengths and limitations of study

This is the first large, population based, long term study of women with early invasive breast cancer describing associations between breast cancer mortality and multiple characteristics of patients and tumours. Women with bilateral, previous, or metastatic cancer were excluded, as were women who did not receive initial surgery. Our results therefore derive from the large majority of women with a diagnosis of breast cancer made during the study period. Most existing studies consider only a few characteristics of patients and tumours, whereas we included all those available from NCRAS, including screening status.

Before analysis, the data underwent rigorous checking by statisticians with input from a surgeon, a pathologist, and three oncologists. We queried inconsistent or missing values with NCRAS staff and corrected them wherever possible. For any remaining missing values, we used multiple imputation. This method enables all women to be included in the analysis, taking account of any correlations between the missing variable and the variables that are known. It also allows for the uncertainty arising from the missing values in confidence intervals and significance tests.

Our study provides an accurate picture of breast cancer mortality in a complete population of women for up to 20 years. During this period, the biology of the disease may have changed owing to hormonal changes arising from obesity, the use of hormone replacement therapy, and reproductive factors.^17^ New systemic therapies, including aromatase inhibitors and taxanes, have become established in clinical use,^18^ and surgery and radiotherapy have become better targeted. These changes in treatment undoubtedly account for some of the decrease in breast cancer mortality seen over the study period. The use of trastuzumab in HER2 positive disease has also increased since the mid-2000s, and our results indicate that women with HER2 positive disease had lower breast cancer mortality than those with HER2 negative disease after adjustment for other factors, probably owing to the use of trastuzumab. Other factors, such as increased breast awareness, screening, and more sensitive breast imaging techniques, are also likely to have contributed to reducing breast cancer mortality but may in some cases have led just to earlier diagnosis and apparently longer survival without, in fact, changing the clinical course of the disease. Notably, however, the improvements in breast cancer mortality seen in women with screen detected cancers were paralleled by improvements in those whose cancers were not screen detected. Therefore, increases in screening cannot solely explain the decreases in breast cancer mortality that we observed. To account for the possible effect of stage migration, we also repeated key analyses in all women with unilateral breast cancer, including those with metastatic disease or who received neoadjuvant treatments. In these analyses, breast cancer mortality still decreased according to calendar period of diagnosis. Therefore, stage migration is not solely responsible for the observed decreases in breast cancer mortality. Neither can the decreases be attributed only to changes in tumour size, number of positive nodes, or tumour grade, as breast cancer mortality still decreased with calendar period of diagnosis, even after adjustment for these factors. Beyond these considerations, this observational study cannot determine the specific causes of these reductions in mortality. However, the main aim of our study was not to quantify the causal role played by different factors in the observed decreases in the breast cancer mortality rate. Instead, we provide information for clinicians to use when estimating breast cancer mortality risks for patients today, taking into account the characteristics of their tumour, including whether the cancer was screen detected. Our study provides, for the first time, estimates of prognosis based on all patients recently receiving a diagnosis of breast cancer in the whole of England.

A limitation of our study was that data on cancer recurrence were not available. Also, we were unable to include women who received neoadjuvant therapy, as pathological tumour size and number of positive axillary nodes at diagnosis were unavailable for them, and we could not include women simultaneously with a diagnosis of more than one cancer in these analyses.

### Comparison with other studies

In recent years, the age standardised annual incidence rate of female breast cancer in the whole UK population rose from 137 per 100 000 in 1993-95 to 169 in 2016-18, whereas the corresponding breast cancer mortality rate in the whole UK population decreased steadily from 53 per 100 000 in 1993-95 to 34 in 2016-18.^19^ Therefore, the finding of a decrease in breast cancer specific mortality in our study of women with a diagnosis of breast cancer is not unexpected. However, national summary statistics provide little insight into the nature of the decrease—for example, whether decreases have occurred for women with early invasive breast cancer as well as those with metastatic disease or whether they have occurred in oestrogen receptor negative as well as oestrogen receptor positive disease. A literature search identified 10 studies published since 2000, each including at least 5000 women, that examined breast cancer mortality in patients with early invasive breast cancer in population based cancer registries or randomised trials (table S11).^2^ ^3^ ^4^ ^5^ ^6^ ^7^ ^8^ ^9^ ^10^ ^11^ Most studies focused on just a few specific factors, and the extent to which adjustment was made for other factors was variable. Up-to-date population based estimates of breast cancer mortality for groups of patients with individual tumour characteristics and with a recent diagnosis were unavailable. Several studies reported lower breast cancer mortality for women with more recent diagnoses,^3^ ^4^ ^6^ ^8^ ^9^ and individual studies reported mortality patterns similar to those we have reported for oestrogen receptor positive versus oestrogen receptor negative disease,^5^ ^10^ age at diagnosis,^5^ ^7^ ^9^ tumour size,^2^ ^11^ number of positive nodes,^7^ ^11^ and tumour grade.^5^ ^7^ ^11^ One study showed a smaller increase in survival according to calendar period for women aged over 70 years at diagnosis than for younger women.^9^ In our data, the lack of any decrease in mortality for women aged 80-89 years with oestrogen receptor negative disease may be because these women do not usually receive adjuvant systematic therapy or radiotherapy,^20^ so any improvements in these treatments would not have affected mortality in this patient group. Patients aged less than 40 years at diagnosis had higher breast cancer mortality risk than patients in their 40s at diagnosis, after adjustment for patient and tumour related factors.^7^ ^9^ This may be because breast cancers in younger women are intrinsically more aggressive than those in older women. Several studies have built models that focus on estimating the effects of treatment and so are not comparable with our study.^21^ ^22^

### Implications of findings

Our findings illustrate the substantial improvement in prognosis for women with a diagnosis of early invasive breast cancer that has been made since the 1990s. More than six in 10 of the women given the diagnosis in England during 2010-15 had a risk of dying from breast cancer during the following five years of 3% or less, and nearly nine in 10 had a risk below 10%. Our findings have several implications.

Firstly, these breast cancer mortality risks inform patients today about their likely prognosis. For example, the estimated five year breast cancer mortality risk for a woman aged 60 at diagnosis, with a screen detected tumour, size <20 mm, low grade, oestrogen receptor positive, HER2 negative, and node negative would be 0.2% (table S9b). This is likely to provide her with reassurance about her prognosis. Such estimates are also relevant to decisions about treatment. Most women with breast cancer are cured by surgery, but undetected microscopic disease remains after surgery in some women, and adjuvant therapies such as chemotherapy, radiotherapy, and endocrine therapy can eradicate this, reducing long term breast cancer mortality risk. Many patients receive several adjuvant treatments. Randomised trials have shown that most adjuvant breast cancer therapies reduce breast cancer mortality by similar proportional amounts in both women with good prognosis and those with a poor prognosis.^23^ ^24^ ^25^ By contrast, the absolute benefits of a given treatment will vary according to prognosis; for a patient with poor prognosis, the absolute mortality benefit from a particular treatment will be greater than for a patient with good prognosis. The decreases in breast cancer mortality during the past few decades suggest that the absolute benefit conferred by each individual adjuvant treatment being considered is likely to be smaller now than in the past.

Secondly, low event rates may make achieving adequate power in randomised trials difficult. Current and planned breast cancer trials may therefore need to recruit large numbers of women to meet their objectives. The likely event rates can be estimated using our data. For example, for a trial including women with tumour size 1-20 mm, grade 1 or 2, node negative, and oestrogen receptor positive disease, five year breast cancer mortality and its confidence interval may be estimated by combining the relevant rows in table S9. In this example, combining rows provides an estimated five year breast cancer mortality of 0.66% (95% confidence interval 0.59% to 0.73%). Details of the method are given in section 6.3 of text S2.

Thirdly, these decreases in breast cancer mortality have implications for decision aids, which are used in the clinic to estimate the absolute benefits of adjuvant treatments for individual patients. These models combine proportional treatment effects in past randomised trials with breast cancer mortality risks in observational data to estimate absolute treatment effects for individual patients. These models need to include breast cancer mortality risks in women with a recent diagnosis, while taking into account the long term mortality patterns seen in women with diagnoses made in the more distant past.

Finally, our findings can be used to reassure most women treated for early breast cancer that they are likely to become long term survivors. They can also be used to identify the groups of women for whom the risk of breast cancer mortality remains substantial.

## What is already known on this topic

The risk of mortality from breast cancer after a diagnosis of early invasive breast cancer has decreased during the past few decades
The extent of the decrease in mortality is unknown, as is whether it is limited to patients with certain characteristics or whether it applies to all patients
Detailed, population based estimates of breast cancer mortality risks according to routinely available factors are unavailable for patients treated recently

## What this study adds

Since the 1990s, the five year risk of death from breast cancer has decreased from 14.4% to 4.9% overall, with reductions seen in nearly all patient groups
Five year risks of death from breast cancer in patients with a recent diagnosis varied widely and were under 3% for 62.8% of women but 20% or higher for 4.6% of women

## Data Availability

De-personalised study data may be made available on request to accredited researchers who submit a proposal that is approved by NHS England’s Data Access Request Service (DARS).

## Web extra

Extra material supplied by authors

Web appendix: Supplementary materials

## Notes

Contributors: CT, PM, GM, DJC, and SCD contributed to the study conceptualisation and design. PM, JP, JB, JC, DJC, GM, and DD contributed to the data collection and collation. PM, JP, and SCD contributed to the data analysis. CT, SD, and AJK contributed to the literature review. CT, PM, JP, SCD, TW, DJC, GM, and DD participated in data interpretation and manuscript preparation. All authors read and approved the final version. CT, PM, and JP contributed equally (joint first authors). DJC, GM, and DD contributed equally (joint last authors). CT and PM are the guarantors. The corresponding author attests that all listed authors meet authorship criteria, and that no others meeting the criteria have been omitted.

The lead authors affirm that this manuscript is an honest, accurate and transparent account of the study being reported; that no important aspects of the study have been omitted; and that any discrepancies from the study as planned (and, if relevant, registered) have been explained.

Dissemination to participants and related patient and public communities: The results have already been presented to representatives of appropriate patient groups. After publication, they will be freely available both to stakeholders and to the broader public.

Provenance and peer review: Not commissioned; externally peer reviewed.

## References

[1] The International Agency for Research on Cancer. Breast fact sheet. 2020. https://gco.iarc.fr/today/data/factsheets/cancers/20-Breast-fact-sheet.pdf.

[2] Sopik V Narod SA . The relationship between tumour size, nodal status and distant metastases: on the origins of breast cancer. Breast Cancer Res Treat 2018;170:647-56. 10.1007/s10549-018-4796-9 29693227 PMC6022519

[3] Holleczek B Brenner H . Trends of population-based breast cancer survival in Germany and the US: decreasing discrepancies, but persistent survival gap of elderly patients in Germany. BMC Cancer 2012;12:317. 10.1186/1471-2407-12-317 22838641 PMC3522526

[4] van der Meer DJ Kramer I van Maaren MC . Comprehensive trends in incidence, treatment, survival and mortality of first primary invasive breast cancer stratified by age, stage and receptor subtype in the Netherlands between 1989 and 2017. Int J Cancer 2021;148:2289-303. 10.1002/ijc.33417 33252836 PMC8048677

[5] Ai B Wang X Kong X Wang Z Fang Y Wang J . Conditional Survival of female patients with operable invasive Breast Cancer in US: A population-based study. J Cancer 2020;11:5782-91. 10.7150/jca.46183 32913471 PMC7477435

[6] Guo F Kuo YF Shih YCT Giordano SH Berenson AB . Trends in breast cancer mortality by stage at diagnosis among young women in the United States. Cancer 2018;124:3500-9. 10.1002/cncr.31638 30189117 PMC6191354

[7] Rose BS Jiang W Punglia RS . Effect of lymph node metastasis size on breast cancer-specific and overall survival in women with node-positive breast cancer. Breast Cancer Res Treat 2015;152:209-16. 10.1007/s10549-015-3451-y 26041688

[8] Coleman MP Forman D Bryant H ICBP Module 1 Working Group . Cancer survival in Australia, Canada, Denmark, Norway, Sweden, and the UK, 1995-2007 (the International Cancer Benchmarking Partnership): an analysis of population-based cancer registry data. Lancet 2011;377:127-38. 10.1016/S0140-6736(10)62231-3 21183212 PMC3018568

[9] Nordenskjöld AE Fohlin H Arnesson LG . Breast cancer survival trends in different stages and age groups - a population-based study 1989-2013. Acta Oncol 2019;58:45-51. 10.1080/0284186X.2018.1532601 30513223

[10] van Maaren MC Strobbe LJA Smidt ML Moossdorff M Poortmans PMP Siesling S . Ten-year conditional recurrence risks and overall and relative survival for breast cancer patients in the Netherlands: Taking account of event-free years. Eur J Cancer 2018;102:82-94. 10.1016/j.ejca.2018.07.124 30144661

[11] Pan H Gray R Braybrooke J EBCTCG . 20-Year Risks of Breast-Cancer Recurrence after Stopping Endocrine Therapy at 5 Years. N Engl J Med 2017;377:1836-46. 10.1056/NEJMoa1701830 29117498 PMC5734609

[12] McGale P Cutter D Darby SC Henson KE Jagsi R Taylor CW . Can observational data replace randomized trials? J Clin Oncol 2016;34:3355-7. 10.1200/JCO.2016.68.8879 27458289

[13] National Cancer Registration and Analysis Service. National Disease Registration Service, NHS England. Cancer. 2023. https://digital.nhs.uk/ndrs/about/ncras.

[14] Bleyer A Welch HG . Effect of three decades of screening mammography on breast-cancer incidence. N Engl J Med 2012;367:1998-2005. 10.1056/NEJMoa1206809 23171096

[15] Zhao A Larbi M Miller K O’Neill S Jayasekera J . A scoping review of interactive and personalized web-based clinical tools to support treatment decision making in breast cancer. Breast 2022;61:43-57. 10.1016/j.breast.2021.12.003 34896693 PMC8669108

[16] Kerr AJ Dodwell D McGale P . Adjuvant and neoadjuvant breast cancer treatments: A systematic review of their effects on mortality. Cancer Treat Rev 2022;105:102375. 10.1016/j.ctrv.2022.102375 35367784 PMC9096622

[17] Ravdin PM Cronin KA Howlader N . The decrease in breast-cancer incidence in 2003 in the United States. N Engl J Med 2007;356:1670-4. 10.1056/NEJMsr070105 17442911

[18] Burton R Stevenson C . Assessment of breast cancer mortality trends associated with mammographic screening and adjuvant therapy from 1986 to 2013 in the state of Victoria, Australia. JAMA Netw Open 2020;3:e208249. 10.1001/jamanetworkopen.2020.8249 32573707 PMC7312387

[19] Cancer Research UK. Breast Cancer Statistics, https://www.cancerresearchuk.org/health-professional/cancer-statistics/statistics-by-cancer-type/breast-cancer.

[20] National Audit of Breast Cancer in Older Patients. 2020 Annual report. https://www.nabcop.org.uk/content/uploads/2021/03/NABCOP-2020-Annual-Report-V1.1_high-res.pdf.

[21] Wishart GC Azzato EM Greenberg DC . PREDICT: a new UK prognostic model that predicts survival following surgery for invasive breast cancer. Breast Cancer Res 2010;12:R1. 10.1186/bcr2464 20053270 PMC2880419

[22] Alaa AM Gurdasani D Harris AL Rashbass J Van der Schaar M . Machine learning to guide the use of adjuvant therapies for breast cancer. Nat Mach Intell 2021;31:716-26 10.1038/s42256-021-00353-8.

[23] Peto R Davies C Godwin J Early Breast Cancer Trialists’ Collaborative Group (EBCTCG) . Comparisons between different polychemotherapy regimens for early breast cancer: meta-analyses of long-term outcome among 100,000 women in 123 randomised trials. Lancet 2012;379:432-44. 10.1016/S0140-6736(11)61625-5 22152853 PMC3273723

[24] Darby S McGale P Correa C Early Breast Cancer Trialists’ Collaborative Group (EBCTCG) . Effect of radiotherapy after breast-conserving surgery on 10-year recurrence and 15-year breast cancer death: meta-analysis of individual patient data for 10,801 women in 17 randomised trials. Lancet 2011;378:1707-16. 10.1016/S0140-6736(11)61629-2 22019144 PMC3254252

[25] Davies C Godwin J Gray R Early Breast Cancer Trialists’ Collaborative Group (EBCTCG) . Relevance of breast cancer hormone receptors and other factors to the efficacy of adjuvant tamoxifen: patient-level meta-analysis of randomised trials. Lancet 2011;378:771-84. 10.1016/S0140-6736(11)60993-8 21802721 PMC3163848

