# Supplementary material for: Breast cancer mortality in 500 000 women with early invasive breast cancer diagnosed in England, 1993-2015: population based observational cohort study
Source: BMJ. 2023 Jun 13;381:e074684. doi: 10.1136/bmj-2022-074684 (PMC10261971; doi:10.1136/bmj-2022-074684)
Supplement: Supplementary file 1 — Web appendix: Supplementary materials [file tayc074684.ww1.pdf]

Breast Cancer Mortality in 500,000 Women Diagnosed with Early Invasive Breast Cancer in England, 1993-2015: Population Based Observational Cohort Study

Supplementary Appendix

	Statistical Methods and Basic Tabulations	Page
Text S1	Data collation and checking	5
Figure S1	Crude all-cause (blue), breast cancer (red) and non-breast-cancer (green) mortality rates in 512,447 women with early breast cancer by combinations of numbers of positive nodes and tumour size, for categories of attained age	7
Figure S2	Composition of the study population among women diagnosed with invasive breast cancer in England during January 1993 to December 2015	8
Text S2	Statistical Methods	9
Table S1	Patterns of missing values in the study population of 512,447 women with early breast cancer	15
Table S2	Characteristics of the study population of 512,447 women with early breast cancer, showing unknown values before multiple imputation, by calendar period of breast cancer diagnosis	16
Figure S3	Comparison of raw and smoothed crude annual breast cancer mortality rates in 512,447 women with early breast cancer by time since diagnosis, according to calendar period of diagnosis	17
Table S3	Characteristics of the study population of 512,447 women with early breast cancer by screening status and calendar period of breast cancer diagnosis	18
	**Breast Cancer Mortality**	
Table S4	Numbers of women diagnosed with early breast cancer, crude annual breast cancer mortality rates and cumulative breast cancer mortality risks by calendar period of diagnosis, and time since diagnosis	22
Table S5	Numbers of women diagnosed with early breast cancer at ages 50-64 years, crude annual breast cancer mortality rates and cumulative breast cancer mortality risks by screening status, calendar period of diagnosis, and time since diagnosis	23
Table S6	Numbers of women diagnosed with early breast cancer, crude annual breast cancer mortality rates and cumulative breast cancer mortality risks by ER status, and time since diagnosis	25
Figure S4	Crude annual breast cancer mortality rates and cumulative breast cancer mortality risks in 512,447 women with early breast cancer by time since diagnosis, according to ER status and screening status	26
Figure S5	Crude annual breast cancer mortality rates and cumulative breast cancer mortality risks in 512,447 women with early breast cancer with ER-positive or ER-negative disease by time since diagnosis, according to calendar period of diagnosis	27
Figure S6	Adjusted annual breast cancer mortality rates in women with early breast cancer with ER-positive or ER-negative disease, according to breast cancer laterality, index of multiple deprivation and region of residence	28
Figure S7	Adjusted annual breast cancer mortality rates in women with early breast cancer with ER-positive or ER-negative disease according to nine characteristics, and time since diagnosis	29
Figure S8	Adjusted annual breast cancer mortality rates during 0-5 years after diagnosis in women with early breast cancer with ER-positive or ER-negative disease according to nine characteristics, and time since diagnosis	30

Figure S9	Adjusted annual breast cancer mortality rates during 5-15 years after diagnosis in women with early breast cancer with ER-positive or ER-negative disease according to nine characteristics, and time since diagnosis	31
Figure S10	Adjusted annual breast cancer mortality rates during 15+ years after diagnosis in women with early breast cancer with ER-positive or ER-negative disease according to nine characteristics, and time since diagnosis	32
Figure S11	Adjusted annual breast cancer mortality rates during 0-5 years after diagnosis in women with early breast cancer with ER-positive or ER-negative disease by calendar period of diagnosis, according to age at diagnosis	33
Figure S12	Adjusted annual breast cancer mortality rates in women with early breast cancer with ER-positive or ER-negative disease by calendar period of diagnosis, according to breast cancer laterality, index of multiple deprivation and region of residence	34
Figure S13	Adjusted annual breast cancer mortality rates in women with early breast cancer with ER-positive or ER-negative disease by calendar period of diagnosis, according to age at diagnosis	35
Figure S14	Adjusted annual breast cancer mortality rates in women with early breast cancer with ER-positive or ER-negative disease by calendar period of diagnosis, according to screening status	36
Figure S15	Adjusted annual breast cancer mortality rates in women with early breast cancer with ER-positive or ER-negative disease by calendar period of diagnosis, according to tumour size	37
Figure S16	Adjusted annual breast cancer mortality rates in women with early breast cancer with ER-positive or ER-negative disease by calendar period of diagnosis, according to number of positive nodes	38
Figure S17	Adjusted annual breast cancer mortality rates in women with early breast cancer with ER-positive or ER-negative disease by calendar period of diagnosis, according to tumour grade	39
Figure S18	Adjusted annual breast cancer mortality rates in women with early breast cancer with ER-positive or ER-negative disease by calendar period of diagnosis, according to breast cancer laterality	40
Figure S19	Adjusted annual breast cancer mortality rates in women with early breast cancer with ER-positive or ER-negative disease by calendar period of diagnosis, according to index of multiple deprivation	41
Figure S20	Adjusted annual breast cancer mortality rates in women with early breast cancer with ER-positive or ER-negative disease by calendar period of diagnosis, according to region of residence	42
Table S7	Tests for interactions in adjusted annual breast cancer mortality rates between pairs of characteristics in women with early breast cancer with ER-positive or ER-negative disease	43
Figure S21	Adjusted annual breast cancer mortality rates in women diagnosed with early breast cancer during 2010-2015, with ER-positive or ER-negative disease according to nine characteristics, and time since diagnosis	44
Table S8	Tests for interactions in adjusted annual breast cancer mortality rates between pairs of characteristics in women diagnosed with early breast cancer during 2010-2015, with ER-positive or ER-negative disease	45
Table S9	Numbers of women diagnosed with early breast cancer during 2010-2015 and five-year cumulative breast cancer mortality risks by categories of tumour grade, size and number of positive nodes in women with ER-positive or ER-negative disease. Tables are split by HER2 status, age and screening status	46
Table S10	Distribution of cumulative five-year breast cancer mortality risks for women diagnosed with early breast cancer during 2010-2015	54
Figure S22	Cumulative five-year breast cancer mortality risks in 156,338 women with early breast cancer diagnosed during 2010-2015 by categories of tumour grade, size and number of positive nodes in women with ER-positive or ER-negative disease. Figures are split by HER2 status, age and screening status. Y-axes are plotted using square-root scale.	55

Other Causes of Mortality

Figure S23	Crude annual rates and cumulative risks of (a) breast cancer mortality (b) non-breast-cancer mortality and (c) all-cause mortality in 512,447 women with early breast cancer by time since diagnosis according to calendar period of diagnosis	57
Figure S24	Crude annual all-cause mortality rates and cumulative all-cause mortality risks in 512,447 women with early breast cancer by time since diagnosis according to calendar period of diagnosis: (a) for all women; (b and c) for women aged 50-64 years (who would all have been eligible for screening) according to whether or not their cancer was screen-detected; and (d) according to ER status	58
Figure S25	Crude annual rates and cumulative risks of (a) breast cancer mortality (b) non-breast-cancer mortality and (c) all-cause mortality in 693,362 women, including 512,447 women with early breast cancer, 138,911 with probable metastatic disease, and 42,004 recorded as receiving neoadjuvant therapy, by time since diagnosis according to calendar period of diagnosis	59
Figure S26	Adjusted annual all-cause mortality rates in women with early breast cancer with ER-positive or ER-negative disease, by various characteristics	60
Figure S27	Adjusted annual all-cause mortality rates in women with early breast cancer with ER-positive or ER-negative disease, by breast cancer laterality, index of multiple deprivation and region of residence	61
Figure S28	Adjusted annual all-cause mortality rates in women with early breast cancer with ER-positive or ER-negative disease according to nine characteristics, and time since diagnosis	62
Figure S29	Adjusted annual all-cause mortality rates in women with early breast cancer with ER-positive or ER-negative disease by calendar period of diagnosis, according to various characteristics	63
Figure S30	Adjusted annual all-cause mortality rates and breast cancer mortality rates in women with early breast cancer with ER-positive or ER-negative disease by calendar period of diagnosis, according to age at diagnosis	65
Figure S31	Cumulative five-year all-cause mortality risks in 156,338 women with early breast cancer diagnosed during 2010-2015 by categories of tumour grade, size and number of positive nodes in women with ER-positive or ER-negative disease. Figures are split by HER2 status, age and screening status	66

Comparable Studies

Table S11	Other studies of breast cancer specific mortality in populations of patients with breast cancer or of relative mortality in populations of patients with breast cancer compared with the general population	68

Statistical Methods and Basic Tabulation

Text S1: Data collating and checking

1. Datafiles

The central dataset received from the National Cancer Registration and Analysis Service (NCRAS) contained one record for every woman registered with invasive breast cancer during the period 1 January 1993 to 31 December 2015. Women who had previously been registered with an invasive cancer were excluded. The file contained a pseudonymised patient identifier, a pseudonymised tumour identifier for each woman's first ('index') breast cancer, and details of that cancer. A total of eighteen other datasets were also received from NCRAS. Each record in these datasets either related to an individual woman and contained the pseudonymised patient identifier and information about that woman, including any previous non-invasive breast cancers and any subsequent invasive cancers, or related to the index tumour and contained the pseudonymised tumour identifier and information relating to that tumour. Many of the files included multiple records per woman or multiple records per tumour.

To create a single datafile for use in analyses, it was necessary to identify the relevant records and variables in each dataset and merge them together to create a new datafile with a single record per woman. This datafile included:

A. Patient details: age of the woman when the index breast cancer was diagnosed, quintile of multiple deprivation[1] (a measure of poverty) based on her address at diagnosis, region of residence (based on the regions covered by the former regional cancer registries), and, if relevant, month and year of any prior non-invasive breast cancers and of any subsequent invasive cancers, month and year of embarkation or month and year of death, together with cause of death.

B. Tumour details for the index breast cancer: year and month of registration, death certificate only registration (yes/no), screen-detected[2] (yes/no), surgery (mastectomy/breast conserving/none), International Classification of Diseases (ICD)_O2 code, morphology, behaviour, histology (categorised into cancer subtype), grade, pathological tumour size, number of involved axillary nodes, oestrogen receptor (ER) status, laterality, indication of metastatic disease at diagnosis (yes/no), indication of neoadjuvant therapy (yes/no). In all analyses, the status screen-detected=yes was only allocated to women aged 50-64 years at diagnosis plus, from 2005, women aged 65-70 years at diagnosis, in order to reflect the coverage of the National Health Service screening program.

Before conducting any analyses, consistency and range checks were carried out. The checks described below were also conducted. Where appropriate, inconsistencies were queried with NCRAS staff for clarification.

2. Review of diagnostic information

For all the index cancers, and wherever there was an indication that the woman had been diagnosed with a previous non-invasive breast cancer, all the available pathological and diagnostic information for that tumour was reviewed by a team of three oncologists, while a pathologist reviewed all the histology codes. Any inconsistencies were discussed with NCRAS staff and, if required, the information recorded was corrected.

For any women recorded as having a second cancer, the date of the first subsequent primary cancer (ignoring non-melanoma skin cancer, records of a diagnoses with no accompanying cancer site, and non-invasive tumours) was ascertained. Women whose index cancer was followed by a second primary cancer or a contralateral invasive breast cancer within 3 months were excluded from the study.

3. Tumour characteristics

Information on tumour characteristics was reviewed. Undifferentiated/anaplastic cancers were grouped with high grade cancers. Nodal status was categorised based on the number of involved axillary nodes irrespective of the number excised. Tumour size and nodal status information were in accordance with TNM staging, allowing TN stage to be defined from these categories if needed .Breast cancer specimens with >10% positively staining cells, or an Allred score of 3-8, were considered to be ER-positive. Those with borderline ER status were categorised as ER-negative.

4. Treatment information

Detailed systemic therapy information was rarely recorded. However, some information on the treatments was available, although it was scattered across multiple datafiles. To make the best possible use of this information, all treatments with an event date within a year of the registration date of the index cancer were identified. Any indication that the woman had received palliative therapy or care was noted. Drug names were also investigated. To do this, a standard drug name dictionary was established and then drug names recorded in the data were matched against it using approximate string matching ("fuzzy" matching). A team of three oncologists with expertise in breast cancer adjudicated any matches with a medium or low score. Records containing drug names associated with the treatment of metastatic disease were noted. The flagged information was then reviewed in conjunction with its timing in relation to the date of cancer registration. Any woman judged likely to have had metastatic cancer at diagnosis was flagged. Women likely to have received neoadjuvant therapy were also flagged.

5. Underlying cause of death

Most causes of deaths were coded according to either the ninth or the tenth revision of ICD. Some regional cancer registries had, however, used different coding systems for the early years of the study and NCRAS provided tables to convert these to ICD10 codes. Deaths coded as ICD9 174 or ICD10 C50 were considered to be deaths from breast cancer.

If a single ICD code was supplied as the underlying cause then this was accepted. Where multiple ICD codes were given as the underlying cause, or where no underlying cause was given but some ICD codes were given in sections 1a-c of the death certificate then, if cancer was mentioned, it took priority. Cancer codes that specified the site of the cancer took priority over cancers of unspecified site, e.g. breast cancer over any secondary cancer and, if more than one primary cancer site was specified, the first was taken. Finally, if 1a-c were all empty, information in part 2 was considered. Examining patterns of cause of death by attained age and stage did not suggest any strong biases in assigning death from breast cancer as underlying cause (see Figure S1).

6. Plausibility of tumour characteristics

Kaplan-Meier curves for breast cancer mortality were constructed for each variable to check the plausibility of the coding. It was expected that the failure curves would be ordered by highest to lowest value of the variable i.e. high grade tumours would have higher mortality than medium grade etc. Ordering of the risk was re-examined after adjustment for age and year of diagnosis. These plots confirmed the plausibility of the coding (data not shown).

References:

1. Noble S, McLennan D, Noble M, Plunkett E, Gutacker N, Silk M, Wright G. (2019). The English Indices of Deprivation 2019: Research Report, London: Ministry of Housing, Communities & Local Government

2. Advisory Committee on Breast Cancer Screening (2006) Screening for breast cancer in England: past and future. NHS Cancer Screening Programmes. http://www.cancerscreening.nhs.uk (Accessed 8th Apr 2022)

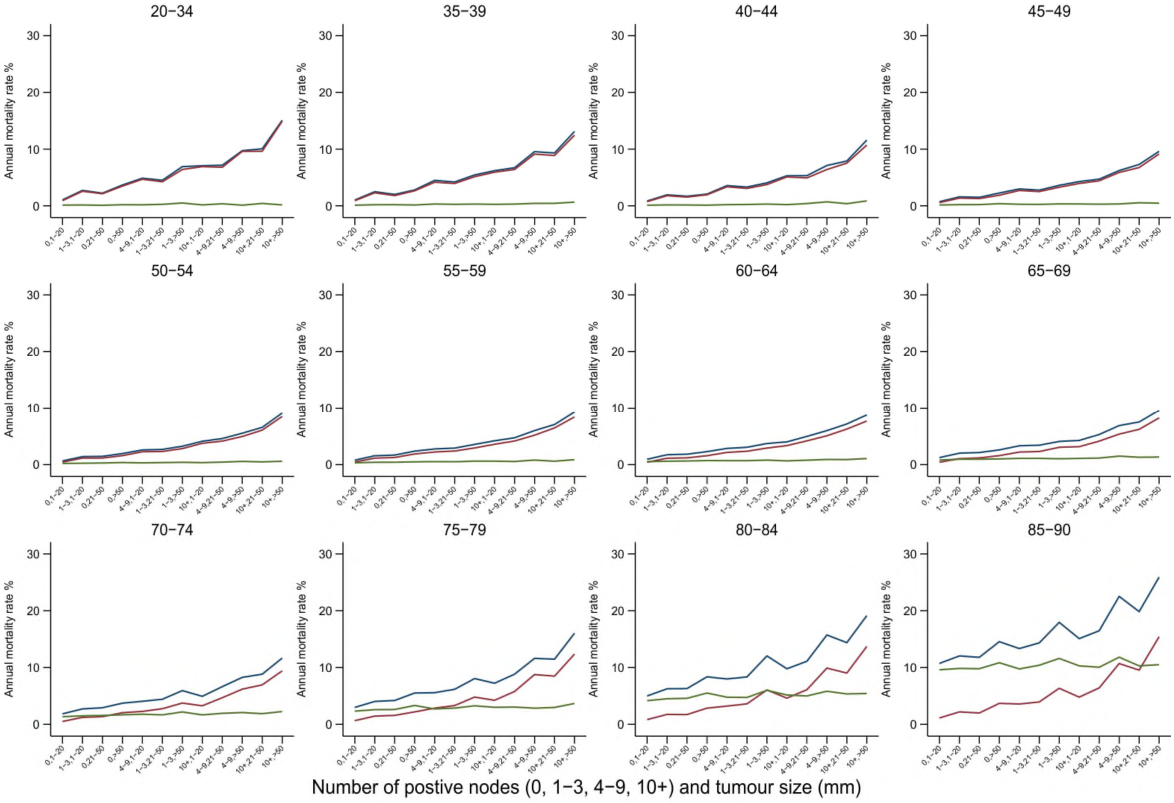

Figure S1: Crude all-cause (blue), breast cancer (red) and non-breast-cancer (green) mortality rates in 512,447 women with early breast cancer by combinations of numbers of positive nodes and tumour size, for categories of attained age

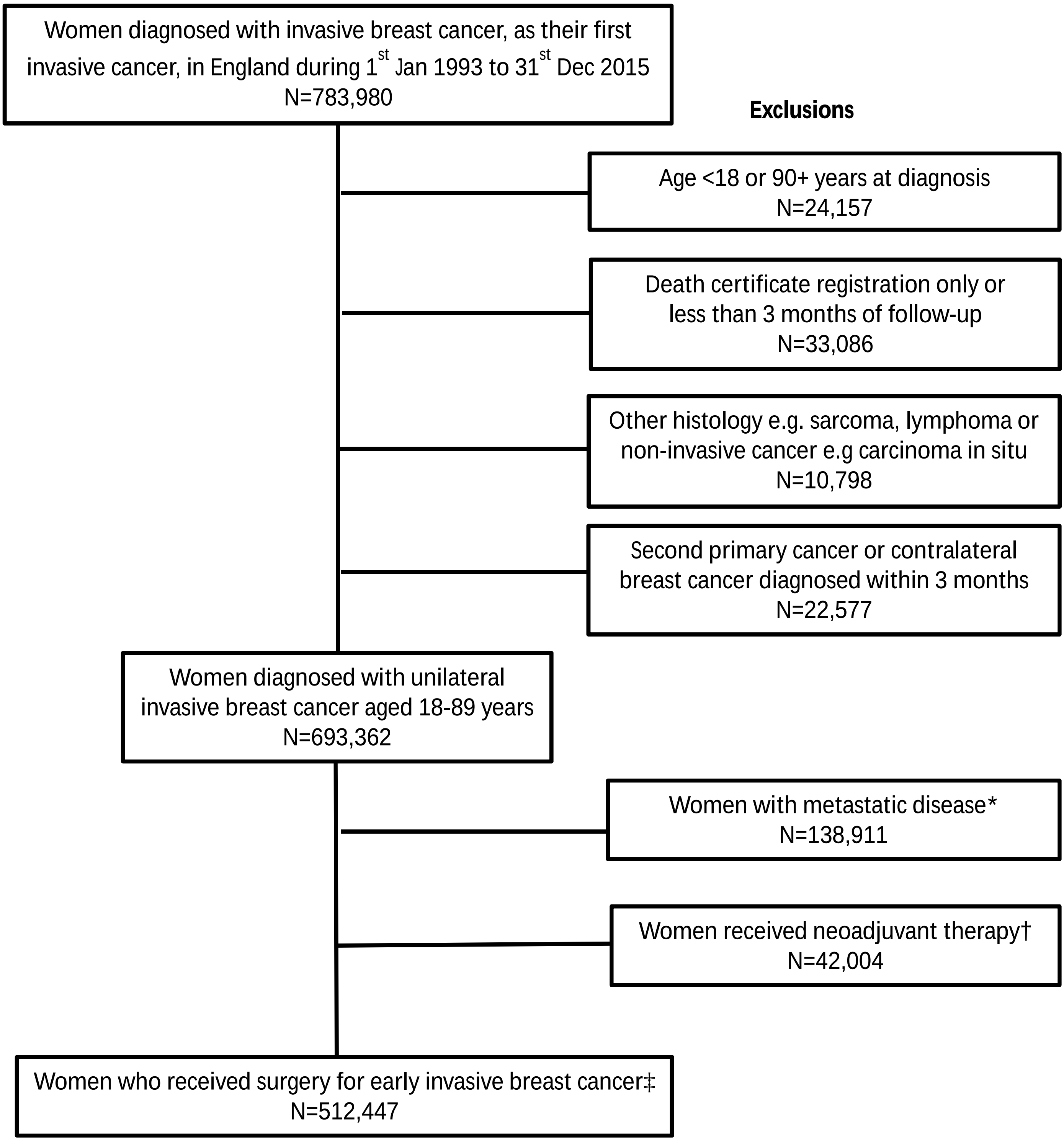

Figure S2: Composition of the study population among women diagnosed with invasive breast cancer in England during January 1993 to December 2015

*Women with metastatic disease were identified as follows: record of metastatic disease, drug usually given for metastatic disease, or palliative radiotherapy within a year of breast cancer diagnosis (N=17,434). This category also includes women with no surgery recorded (N=121,477). Reasons for no surgery may include metastatic disease, and treatment of ER-positive disease with endocrine therapy only in some elderly women.

†Women recorded as receiving neoadjuvant therapy (chemotherapy, endocrine therapy, targeted therapy or radiotherapy) were analysed separately because pre-treatment staging information was unavailable for them. Overall 7.6% of women were recorded as receiving neoadjuvant therapy: 8.1% of women diagnosed during the earlier part of the study (1993-2004) and 7.2% of women diagnosed more recently (2005-2015)

‡Surgery was either breast-conserving surgery or mastectomy

Text S2: Statistical Methods

1. Tabulation of person-years at risk and observed events

For each woman who was eligible for the study, her contribution to the person-years at risk was calculated. Women started to contribute to the person-years at risk from 3 months after diagnosis of breast cancer and stopped on the earliest of: date of death, emigration, 95th birthday or end of follow-up.

In the analysis, calendar period was considered in five-year categories except that women diagnosed during 1993/1994 were included with the 1995-1999 period, and women diagnosed during 2015 were included with the 2010-2014 period. These categories were chosen (before any data were analysed) for the following reasons. Eligibility for screening changed at the beginning of 2005 to include women aged 65-70, so one cut-point was between 2004 and 2005. Then, in considering how to subdivide the period 2005-2015, a cut-point at the beginning of 2010 rather than later was used, as there were fewer deaths for women diagnosed during 2010-2015 than during 2005-2009. Finally, when considering how to subdivide the period 1993-2004, it was decided to make the more recent category the same length as 2005-2009, leading to a cut-point at the beginning of 2000.

It was desirable that, within each calendar period category, the maximum length of follow-up was independent of individual calendar year of diagnosis. Therefore, unless a woman had previously died, emigrated or reached her 95th birthday, she stopped contributing to the person-years at: 21 years from diagnosis if she was diagnosed during 1993-1999, 16 years if she was diagnosed during 2000-2004, 11 years if she was diagnosed during 2005-2009 and 5 years if she was diagnosed during 2010-2015. The individual contributions to the person-years at risk were then added together. The number of women whose contribution to the person-years was terminated by death from breast cancer (or from all causes) was also obtained. Both the numbers of person-years and the numbers of deaths were then tabulated simultaneously according to all the factors considered in the study. It was assumed throughout that the date of each woman's cancer registration was her date of diagnosis.

2. Nature and amount of missing data

The following tables show the nature and amount of missing data:
Tables: S1, S2

Information on the following variables was available for all women in the study: year and month of breast cancer diagnosis, age at breast cancer diagnosis, screening status, quintile of index of multiple deprivation (IMD) and region of residence, together with month and year of emigration if she had emigrated, and month and year of death, with cause of death, if she had died before the end of her follow-up. For 130,745 (25.5%) of the women, information was also available on all the following characteristics: laterality, tumour grade, HER2 status (during the calendar period 2010-2015), tumour size, number of positive lymph nodes, and ER status (Table S1). However, for 26.7%, 28.8%, 14.3%, 4.0%, 0.6% and 0.04% respectively information was missing on 1, 2, 3, 4, 5, or all 6 of these variables.

For each variable with some missing values, the proportion that were missing was strongly correlated with calendar period of cancer diagnosis (Table S2). This trend is likely to have arisen from the increasing effort put into obtaining these characteristics by cancer registration staff in recent years. All the variables concerned would have been collected, or missed, at – or close to - the time the woman was registered with breast cancer.

From 2013 onwards, the Cancer Outcomes and Services Dataset started to enable those providing information on cancer registrations and subsequent cancer treatments to submit their data to NCRAS using a variety of different systems. However, its implementation was phased and it made little

difference to our study, apart from the fact that there were fewer missing values during 2013-2015 than during previous years. Notifications of death dates and causes continued to be supplied to NCRAS from the Office of National Statistics without interruption.

3. Multiple imputation

The following tables and figures use the imputed datasets:
Tables: 1, S3, S6-S10
Figures: 1-8, S4-S22, S24d, S26-S31

In order to be able to include all the women in every analysis, irrespective of whether data on some characteristics were missing, the method of multiple imputation was used.[1,2] In this method, multiple datasets are created in which the missing values are replaced by imputed (i.e. predicted) values that have been sampled from their predictive distributions. The predictive distributions take into account any correlations between the known values of the variables with missing values and other variables in the data, thus enabling the imputed values to take appropriate account of the correlations present in the data. In addition, differences between the different imputed datasets enable the uncertainty arising from the imputed values to be taken into account in any ensuing confidence intervals and significance tests.

All the variables for which any values were missing were categorical and there were no complex design features in the data. For each missing value, its predictive distribution was obtained using chained ordered logistic regressions. Independent variables in the predictions were age at diagnosis, screening status, index of multiple deprivation, region of residence, breast cancer death (yes or no), non-breast-cancer death (yes or no) and the Nelson-Aalen estimate of the cumulative hazard function for breast-cancer deaths. Cancer subtype (16 categories) was also included as an auxiliary variable in the prediction because of its association with ER-status. As the study covered cancer diagnoses during a period of over 20 years, prediction was carried out separately for each calendar period of diagnosis. This allows for possible interactions between calendar period and all the other variables in the prediction model. The assumption was then made that, within each calendar period, missing values in the other random variables in the prediction model occurred at random.

Calculation was carried out using the multiple imputation suite of programs in Stata.[3] The default burn-in period was 10 iterations and, to confirm that this number was sufficient, trace plots for a burn-in period of 100 iterations were produced and examined. To examine the plausibility of the imputed values, their distribution for each variable in every calendar period category was compared with that of the known values for that variable. In every case good agreement was found. In addition, the distributions of the variables for which values were missing were compared with distributions in the published literature for other comparable populations of breast cancer patients and discussed with a number of experienced oncologists. The tables and analyses presented in this report are based on a total of 60 imputed datasets. This number was chosen in accordance with the guidelines recommended by van Buuren ie. that the number of imputations should be similar to the percentage of cases that are incomplete.[2] In order to confirm that it was sufficient, several of the analyses were conducted using both 40 and 100 imputations and the results were found to differ little.

For the variables where imputation was necessary, the tables and figures in this report present the number of women in each category averaged over the imputed datasets. Analyses were carried out separately on each imputed dataset, as described in sections 4. and 5. below. The resulting estimates and their variances were then combined via Rubin's rules.[1] Tests of statistical significance of heterogeneity (screening status and region of residence) or for a linear trend (all other variables) were conducted. These assumed that the between-imputation variance was proportional to the within-imputation variance.[1] No corrections have been made to p-values for multiple testing. However, the

large number of tests that have been conducted should be borne in mind when interpreting our results and, to aid with this, we have presented p-values in scientific notation.

4. Smoothed crude annual breast cancer mortality rates and cumulative risks

The following tables and figures make use of smoothing:
Tables: S4, S5, S6
Figures: 1, S3-S5, S23-S25

Poisson regression[4] was used to estimate the breast cancer mortality rates by time since diagnosis. These rates were then approximated by a continuous function of the logarithm of time since diagnosis, and this function provided a smoothed estimate of the crude annual breast cancer mortality rate for all women combined.

The function was derived by considering restricted cubic splines with three degrees of freedom, interior knots at the 33rd and 66th percentiles of the event time distribution and boundary knots at the minimum and maximum values of time since diagnosis. The appropriate number of interior knots was determined both graphically and formally, using the Bayesian information criterion. Sensitivity analyses confirmed that the shape of the smoothed crude annual breast cancer mortality rate was not sensitive to the placement of the knots. The mortality rate was then estimated for each 1 month interval using Poisson regression with the number of deaths as the dependent variable, the log of the person-years as a fixed offset, and the spline basis variables and covariate of interest as independent variables.

Separate models were fitted for each covariate considered. In each of these models, an interaction between each category of the covariate (e.g. calendar period of diagnosis) and each spline basis variable was included. In this way, the models provided smoothed estimates for the different category levels of the covariate that were not constrained to be proportional to each other.

Before proceeding further, a comparison was made between smoothed and unsmoothed rates to check for any systematic effect that might have been introduced by the smoothing algorithm. These provided reassurance that the changes in the crude rates with time since diagnosis were well described in all the calendar period categories (see e.g. Figure S3).

Where imputation was needed, the mortality rates were calculated as described above within each imputed dataset, and then combined using Rubin's rules.

Cumulative risks were derived by first summing the smoothed mortality rates over time since diagnosis, with weighting proportional to the length of the time-interval covered, to give the cumulative rate, Λ, and then calculating $1-\exp(-\Lambda)$. The standard errors of the cumulative risks were based on the standard errors of the cumulative rates. The cumulative risk calculation for each calendar period category continued until the maximum time since diagnosis, described in section 1 above, was reached.

5. Adjusted annual breast cancer mortality rates

The following figures display adjusted annual mortality rates:
Figures: 2-7, S6-S21, S26-S30

To simplify the calculation of adjusted annual breast cancer mortality rates, a two-stage approach was adopted.

In the first step, separate models were fitted for women with ER-positive and ER-negative disease. In these models, adjusted annual breast cancer mortality rate ratios were estimated using Poisson

regression, with the numbers of deaths as the dependent variable and the log of the person-years, which were assumed to be fixed, as an offset. Time since diagnosis was classified into one-year intervals for the first five years and five-year intervals thereafter, and it was considered as a categorical variable. This variable, and all the other variables listed in Table 1 (apart from ER-status and HER2 status), were included in the model simultaneously as independent variables using the categories displayed in Table 1.

In addition, in order to investigate the pattern of breast cancer mortality with time since diagnosis, a series of models were fitted to the data which included all the variables described above and also a two-way interaction between calendar period of diagnosis and each one of the other variables in turn. These models provided estimates of the adjusted annual mortality rates and their confidence intervals and the rate ratios and their confidence intervals separately for women with ER-positive and ER-negative disease.

In the second step, the estimated coefficients arising from the above models were used to obtain adjusted annual mortality rates for women with ER-positive disease and ER-negative disease that were directly comparable with each other. This was done by means of the technique of adjusted predictions, as described by Williams,[5] and using the Stata command 'margins'. This comprised predicting, for each variable, the number of deaths in each category of the variable for each combination of all the other variables included in the model. The adjusted annual mortality rates were then obtained by averaging these predicted numbers of deaths by the proportions of the total person years in the entire study (i.e. both women with ER-positive and women with ER-negative disease) which had that combination of all the other variables included in the model. This is equivalent to fitting a single model that includes an interaction between ER-status and every other variable, but is much easier and quicker to fit. Further explanation is given in Williams[5] and examples 1 and 15 of the relevant section of the documentation for Stata[3] (available in https://www.stata.com/manuals/rmargins.pdf).

As HER2 status was only available for women diagnosed during 2010-2015, it was not considered in analyses of women diagnosed during the whole study period, 1993-2015. HER2 status was, however, included in a separate analysis just of women diagnosed during 2010-2015.

6. Five-year cumulative mortality risks for 156,338 women diagnosed during 2010-2015

The following tables and figures display cumulative mortality risks for women diagnosed during 2010-2015:
Tables: S9, S10
Figures: 8, S22, S31

For women diagnosed during 2010-2015, the women were categorised simultaneously by all of the five available tumour characteristics (ER status, HER2 status, grade, tumour size and number of positive nodes) as well as by their age and screening status. This gave rise to 576 possible groups of women with different combinations of the available characteristics. Some of these groups contained hundreds or thousands of women, but some contained few women, or no women at all. To avoid basing risk estimates on very sparse data, it was decided to focus just on groups that contained at least 40 women on average across the 60 imputed datasets. There were 253 such groups and they included 97.9% (i.e. 153,006/156,338) of the women diagnosed during 2010-2015.

In 248 of the 253 groups, there was at least one death within five years of breast cancer diagnosis in at least three quarters (i.e. 45) of the 60 imputed datasets. In the remaining 5/253 groups there were no deaths within five years of breast cancer diagnosis in at least 45 of the 60 imputed datasets.

Different methods were used to estimate the five-year cumulative mortality risk for these two different types of group.

6.1 Method for 248 groups with at least one death in at least 45 of the 60 imputed datasets.

The annual mortality rate during the first five years of follow-up (Λ) was calculated by dividing the total number of deaths from breast cancer (or from all causes) by the corresponding number of person-years at risk within each of the 60 imputed datasets. Cumulative annual mortality rates were then calculated as $w\Lambda$, where w is the length of the interval being considered (i.e. 4.75 years, as follow-up began at 3 months after diagnosis and ended at 5 years after diagnosis), and percentage cumulative mortality risks were calculated as $100[1-\exp(-w\Lambda)]$. The standard errors of the cumulative risks were based on the standard errors of the cumulative rates and the estimates and their variances were then combined via Rubin's rules, as in section 3 above.

6.2 Method for 5 groups in which there were no deaths in at least 45 of the 60 imputed datasets.

For these 5 groups, the total numbers of deaths in all 60 imputed datasets were 14, 5, 2, 1 and 1 respectively. The large sample assumptions used for estimating the standard errors in section 6.1 above were, therefore, inappropriate and a simpler, approximate, approach was taken. The annual mortality rate, Λ, in each group was estimated by the sum of the deaths in all the 60 imputed datasets for the group divided by the sum of the person-years in all 60 imputed datasets for the group. The cumulative mortality rate was then estimated as $w\Lambda$, where, as above, w is the length of the interval being considered, and the percentage cumulative mortality risk was estimated as $100[1-\exp(-w\Lambda)]$. An upper 95% confidence limit for the percentage cumulative risk was derived assuming that the total number of deaths across the 60 imputed datasets had a Poisson distribution. Therefore, if O_U is the upper 95% confidence limit for the mean of the underlying Poisson distribution, then the approximate upper limit of the 95% confidence interval for the percentage cumulative mortality risk is $100[1-\exp(-wO_U/P)]$, where P is the total number of person-years observed in all 60 imputed datasets. For these groups the lower limit of the confidence interval was set to zero.

6.3 Estimating risks for broader groups of women

If estimates of the five-year cumulative mortality risk are desired for broader groups of women than those displayed in Table S9, then the following approximate method can be used.

The annual mortality rate, Λ, in the broader group can be estimated by the sum of the numbers of deaths shown in Table S9 for all the groups that are being combined, divided by the sum of the person-years in all the groups that are being combined. The cumulative mortality rate in the broader group is then estimated as $w\Lambda$, where, as above, w is the length of the interval being considered (i.e. 4.75 years), and the percentage cumulative mortality risk can be estimated as $100[1-\exp(-w\Lambda)]$).

If the number of deaths in the groups being combined into the broader group is large (e.g. more than about 15), then an approximate 95% confidence interval for the percentage cumulative risk is $100\{1-\exp(-w(\Lambda \pm 1.96\sqrt{O}\,/P))\}$, where O is the total number of deaths in the groups being combined and P is the total number of person-years. This method does not account for the variance due to the imputation.

Alternatively, if the total number of deaths observed in the broader group is small (e.g. less than about 15), then a 95% confidence interval for the percentage cumulative risk can then be estimated as in section 6.2 above.

For example, for a trial including women with tumour size 1-20 mm, grade 1 or 2, node negative, and oestrogen receptor positive disease, five year breast cancer mortality risk and its confidence interval

may be estimated by combining the relevant rows in table S9. In this example, combining rows provides a total of 350 deaths and 251,737.9 person-years. Hence, the cumulative risk is calculated by

$$100 \times \left(1 - \exp\left(-4.75 \times \frac{350}{251737.9}\right)\right),$$

whilst the confidence interval is calculated by

$$100 \times \left(1 - \exp\left(-4.75 \times \left(\frac{350}{251737.9} \pm \frac{1.96 \times \sqrt{350}}{251737.9}\right)\right)\right)$$

Therefore, these women have an estimated five year breast cancer mortality risk of 0.66% (95% confidence interval 0.59% to 0.73%).

References:

1. Rubin D. B. Multiple Imputation for Nonresponse in Surveys. John Wiley and Sons, New York. 1987.
2. Van Buuren S. Flexible Imputation of Missing Data. Second Edition. CRC/Chapman & Hall, FL: Boca Raton 2018
3. StataCorp. Stata Statistical Software: Release 15.1 [program] StataCorp, Texas. 2017
4. Royston, P. and Lambert P.C. Flexible Parametric Survival Analysis Using Stata: Beyond the Cox Model. Stata Press, College Station, TX. 2011.
5. Williams R. Using the margins command to estimate and interpret adjusted predictions and marginal effects. The Stata Journal, 2012; 12:308-331.

Table S1: Patterns of missing values in the study population of 512,447 women with early breast cancer

Number of variables missing	Variables						Number of women	
	Laterality	Grade	HER2 status	Size	Nodes positive	ER status		
0	1	1	1	1	1	1	130,745	25.5%
1	1	1	1	1	1	0	116,807	
1	1	1	1	1	0	1	5,906	
1	1	1	1	0	1	1	4,933	
1	1	1	0	1	1	1	7,853	
1	1	0	1	1	1	1	1,143	
1	0	1	1	1	1	1	39	
Subtotal							136,681	26.7%
2	1	1	1	1	0	0	111,492	
2	1	1	1	0	1	0	10,748	
2	1	1	1	0	0	1	4,117	
2	1	1	0	1	1	0	14,504	
2	1	1	0	1	0	1	791	
2	1	1	0	0	1	1	669	
2	1	0	1	1	1	0	3,848	
2	1	0	1	1	0	1	168	
2	1	0	1	0	1	1	251	
2	1	0	0	1	1	1	125	
2	0	1	1	1	1	0	969	
2	0	1	1	1	0	1	7	
2	0	1	1	0	1	1	9	
2	0	1	0	1	1	1	1	
Subtotal							147,699	28.8%
3	1	1	1	0	0	0	48,497	
3	1	1	0	1	0	0	8,457	
3	1	1	0	0	1	0	1,863	
3	1	1	0	0	0	1	619	
3	1	0	1	1	0	0	10,546	
3	1	0	1	0	1	0	1,616	
3	1	0	1	0	0	1	381	
3	1	0	0	1	1	0	242	
3	1	0	0	1	0	1	41	
3	1	0	0	0	1	1	59	
3	0	1	1	1	0	0	940	
3	0	1	1	0	1	0	111	
3	0	1	1	0	0	1	26	
3	0	1	0	1	1	0	47	
3	0	0	1	1	1	0	70	
3	0	0	1	0	1	1	2	
Subtotal							73,517	14.3%
4	1	1	0	0	0	0	2,858	
4	1	0	1	0	0	0	16,219	
4	1	0	0	1	0	0	203	
4	1	0	0	0	1	0	156	
4	1	0	0	0	0	1	60	
4	0	1	1	0	0	0	921	
4	0	1	0	1	0	0	39	
4	0	1	0	0	1	0	13	
4	0	1	0	0	0	1	1	
4	0	0	1	1	0	0	138	
4	0	0	1	0	1	0	46	
4	0	0	1	0	0	1	15	
4	0	0	0	1	1	0	5	
4	0	0	0	0	1	1	1	
Subtotal							20,675	4.0%
5	1	0	0	0	0	0	914	
5	0	1	0	0	0	0	77	
5	0	0	1	0	0	0	1,907	
5	0	0	0	1	0	0	3	
5	0	0	0	0	1	0	7	
Subtotal							2,908	0.6%
6	0	0	0	0	0	0	222	0.04%
**Total number of women**							512,447	100.0%

*A value of 1 in a cell indicates data are present and a value of 0 indicates data are absent.

Table S2: Characteristics of the study population of 512,447 women with early breast cancer, showing unknown values before multiple imputation, by calendar period of breast cancer diagnosis

Characteristic		Number of women by calendar period of diagnosis (%) 1993-1999	2000-2004	2005-2009	2010-2015	Breast deaths (%) All years	Any death (%) All years	Women (%) All years
**Age at diagnosis (years)**	18-39	7,852 (7)	7,002 (5)	6,552 (6)	6,717 (3)	6,614 (9)	7,268 (5)	28,123 (5)
	40-49	21,718 (19)	19,313 (17)	22,369 (17)	27,908 (18)	14,337 (18)	17,736 (11)	91,308 (18)
	50-64	47,465 (42)	50,761 (44)	53,741 (42)	62,074 (40)	27,526 (35)	48,076 (31)	214,041 (42)
	65-70	13,385 (12)	14,399 (13)	20,207 (16)	27,419 (18)	9,958 (13)	24,282 (16)	75,410 (15)
	71-79	16,988 (15)	16,703 (15)	17,122 (13)	21,972 (14)	13,456 (17)	39,092 (25)	72,785 (14)
	80-89	5,946 (5)	6,648 (6)	7,938 (6)	10,248 (7)	6,084 (8)	19,441 (12)	30,780 (6)
**Cancer screen-detected**	Eligible: screen-detected	14,981 (13)	20,190 (17)	41,272 (32)	51,797 (33)	7,686 (10)	18,508 (12)	128,240 (25)
	Eligible: not screen-detected	32,484 (29)	30,571 (27)	32,676 (26)	37,696 (24)	22,997 (29)	36,930 (24)	133,427 (26)
	Not eligible for screening	65,889 (58)	64,065 (56)	53,981 (42)	66,845 (43)	47,292 (61)	100,457 (64)	250,780 (49)
**Tumour size (mm)**	1-20	45,901 (41)	53,045 (46)	63,063 (49)	84,562 (54)	20,705 (27)	52,833 (34)	246,571 (48)
	21-50	27,651 (24)	32,999 (29)	40,751 (32)	50,044 (32)	32,162 (41)	54,845 (35)	151,445 (30)
	>50	2,707 (2)	3,483 (3)	4,969 (4)	5,954 (4)	5,517 (7)	7,822 (5)	17,113 (3)
	Unknown	37,095 (33)	25,299 (22)	19,146 (15)	15,778 (10)	19,591 (25)	40,395 (26)	97,318 (19)
**Number of positive nodes**	0	13,987 (12)	26,372 (24)	46,298 (35)	88,208 (57)	10,386 (13)	28,763 (19)	174,865 (34)
	1 to 3	10,910 (10)	16,441 (14)	23,848 (19)	34,052 (22)	13,812 (18)	23,367 (15)	85,251 (17)
	4 to 9	4,213 (4)	6,037 (5)	7,270 (6)	7,939 (5)	8,694 (11)	11,645 (7)	25,459 (5)
	10 or more	1,647 (1)	2,663 (2)	3,423 (3)	3,574 (2)	5,565 (7)	6,755 (4)	11,307 (2)
	Unknown	82,597 (73)	63,313 (55)	47,090 (37)	22,565 (14)	39,518 (51)	85,365 (55)	215,565 (42)
**Tumour grade**	Low	20,151 (18)	21,233 (19)	21,234 (17)	26,219 (17)	4,696 (6)	19,248 (12)	88,837 (18)
	Medium	41,889 (37)	48,495 (42)	59,287 (46)	78,262 (50)	28,540 (37)	62,520 (40)	227,933 (44)
	High	31,616 (28)	34,585 (30)	42,467 (33)	48,621 (31)	36,778 (47)	57,096 (37)	157,289 (31)
	Unknown	19,698 (17)	10,513 (9)	4,941 (4)	3,236 (2)	7,961 (10)	17,031 (11)	38,388 (7)
**Oestrogen-receptor status**	Positive	2,336 (2)	11,942 (11)	20,356 (16)	99,979 (64)	8,135 (11)	17,136 (11)	134,613 (26)
	Negative	610 (1)	2,688 (2)	4,076 (3)	15,975 (10)	4,150 (5)	6,044 (4)	23,349 (5)
	Unknown	110,408 (97)	100,196 (87)	103,497 (81)	40,384 (26)	65,690 (84)	132,715 (85)	354,485 (69)
**HER2 status**	Negative	-	-	-	101,234 (65)	4,332 (5)	8,246 (6)	101,234 (20)
	Positive	-	-	-	15,274 (10)	896 (1)	1,455 (1)	15,274 (3)
	Before 2010	113,354 (100)	114,826 (100)	127,929 (100)	-	70,597 (91)	142,308 (91)	356,109 (69)
	Unknown	-	-	-	39,830 (25)	2,150 (3)	3,886 (2)	39,830 (8)
**Breast cancer laterality**	Left	57,531 (51)	58,198 (50)	65,249 (51)	80,024 (51)	40,253 (52)	80,240 (51)	261,002 (51)
	Right	53,550 (47)	54,895 (48)	61,559 (48)	75,825 (49)	36,567 (47)	73,203 (47)	245,829 (48)
	Other/unknown	2,273 (2)	1,733 (2)	1,121 (1)	489 (0)	1,155 (1)	2,452 (2)	5,616 (1)
**Index of multiple deprivation**	<20% (least deprived)	25,386 (22)	26,034 (23)	29,601 (23)	37,027 (24)	16,217 (21)	30,702 (19)	118,048 (23)
	20-39%	25,389 (22)	26,205 (23)	29,360 (23)	36,112 (23)	17,082 (22)	33,585 (22)	117,066 (23)
	40-59%	23,854 (21)	24,143 (21)	26,987 (21)	32,825 (21)	16,426 (21)	33,151 (21)	107,809 (21)
	60-79%	21,148 (19)	21,131 (18)	23,273 (18)	27,985 (18)	15,087 (19)	31,059 (20)	93,537 (18)
	80+% (most deprived)	17,577 (16)	17,313 (15)	18,708 (15)	22,389 (14)	13,163 (17)	27,398 (18)	75,987 (15)
**Region of residence**	Eastern	12,195 (10)	12,734 (11)	13,623 (10)	18,238 (11)	8,176 (11)	16,134 (11)	56,790 (11)
	North West	16,270 (14)	15,936 (14)	17,514 (14)	20,280 (13)	10,876 (14)	22,926 (15)	70,000 (14)
	Northern & Yorkshire	14,095 (12)	15,521 (14)	17,039 (13)	20,275 (13)	10,093 (13)	20,398 (13)	66,930 (13)
	Oxford	7,776 (7)	6,643 (6)	7,149 (6)	9,374 (6)	4,452 (6)	8,510 (5)	30,942 (6)
	South West	18,712 (17)	17,589 (15)	20,222 (16)	24,520 (16)	12,007 (15)	24,638 (16)	81,043 (16)
	Thames	24,723 (22)	23,093 (20)	27,231 (21)	32,564 (21)	17,285 (22)	33,433 (21)	107,611 (21)
	Trent	5,174 (5)	10,455 (9)	11,218 (9)	15,109 (10)	5,716 (7)	11,362 (7)	41,956 (8)
	West Midlands	14,409 (13)	12,855 (11)	13,933 (11)	15,978 (10)	9,370 (12)	18,494 (12)	57,175 (11)
**End of follow-up (years from diagnosis)**	≤1	3,135 (3)	2,130 (2)	1,688 (1)	1,308 (1)	7,193 (5)	7,193 (5)	8,261 (1)
	1-2	5,166 (5)	3,864 (3)	3,190 (2)	2,695 (2)	14,841 (10)	14,841 (10)	14,915 (3)
	2-3	5,216 (5)	4,057 (4)	3,579 (3)	3,122 (2)	15,942 (10)	15,942 (10)	15,974 (3)
	3-4	5,064 (4)	4,009 (3)	3,778 (3)	3,458 (2)	16,277 (10)	16,277 (10)	16,309 (3)
	4-5	3,959 (3)	3,573 (3)	3,200 (3)	145,755 (93)	13,801 (9)	13,801 (9)	156,487 (31)
	5-10	16,231 (14)	14,874 (13)	15,735 (12)	-	45,320 (29)	45,320 (29)	46,840 (9)
	10-15	12,784 (11)	13,497 (12)	96,759 (76)	-	27,706 (18)	27,706 (18)	123,040 (24)
	15-20	11,229 (10)	68,822 (60)	-	-	12,852 (8)	12,852 (8)	80,051 (16)
	20-21	50,570 (45)	-	-	-	1,963 (1)	1,963 (1)	50,570 (10)
**Total**		**113,354 (100)**	**114,826 (100)**	**127,929 (100)**	**156,338 (100)**	**77,975 (100)**	**155,895 (100)**	**512,447 (100)**

HER2: human epidermal growth factor receptor 2. Data available only for period 2010-2015.

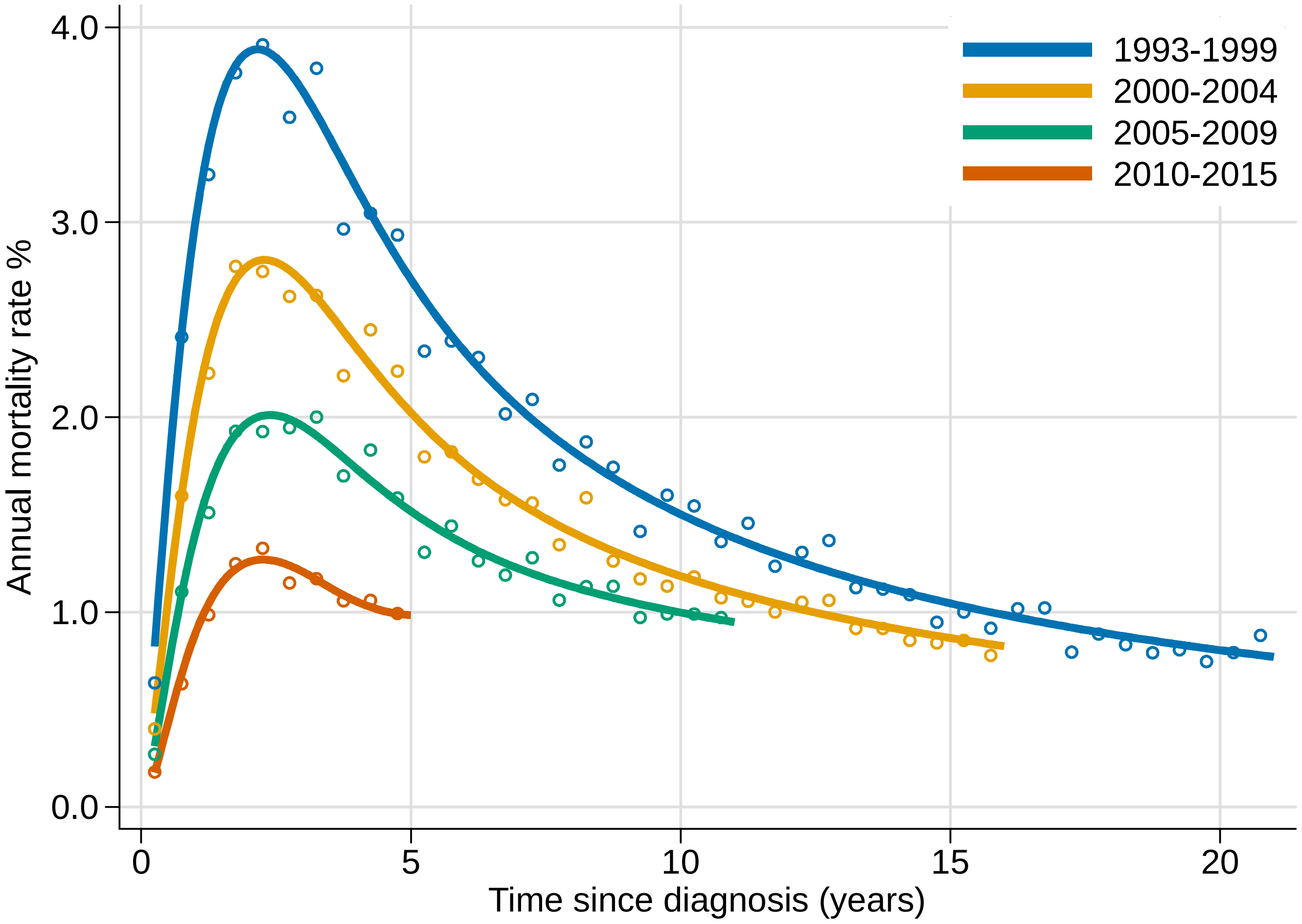

Figure S3: Comparison of raw and smoothed crude annual breast cancer mortality rates in 512,447 women with early breast cancer by time since diagnosis, according to calendar period of diagnosis

Dots are raw rates derived by dividing the numbers of deaths by the numbers of person years in six-month periods. Lines are smoothed rates.

Table S3: Characteristics of the study population of 512,447 women with early breast cancer by screening status and calendar period of breast cancer diagnosis

a) Screen-detected cancers

Characteristic		Percent of women by calendar period of diagnosis 1993-1999	2000-2004	2005-2009	2010-2015	Percent All years	Women All years
**Age at diagnosis (years)**	50-64	100	100	71	67	77	99,105
	65-70	0	0	29	33	23	29,135
**Cancer screen-detected**	Eligible: screen-detected	100	100	100	100	100	128,240
**Tumour size (mm)**	1-20	80	77	76	78	78	99,805
	21-50	19	21	22	20	20	26,284
	>50	1	2	2	2	2	2,151
**Number of positive nodes**	0	62	65	67	77	70	90,157
	1 to 3	29	25	25	19	23	29,305
	4 to 9	7	7	6	3	5	6,347
	10 or more	2	3	2	1	2	2,431
**Tumour grade**	Low	37	33	27	26	29	36,681
	Medium	44	48	52	54	51	65,971
	High	19	19	21	20	20	25,588
**Oestrogen-receptor status**	Positive	81	89	90	92	90	114,901
	Negative	19	11	10	8	10	13,339
**HER2 status**	Negative	0	0	0	90	36	46,585
	Positive	0	0	0	10	4	5,212
	Before 2010	100	100	100	0	60	76,443
**Breast cancer laterality**	Left	51	51	51	51	51	65,583
	Right	49	49	49	49	49	62,657
**Index of multiple deprivation**	<20% (least deprived)	24	24	23	25	24	30,626
	20-39%	23	24	24	23	24	30,233
	40-59%	21	22	21	21	21	27,423
	60-79%	18	18	18	18	18	22,774
	80+% (most deprived)	14	12	14	13	13	17,184
**Region of residence**	Eastern	12	13	11	12	11	15,381
	North West	0	2	12	13	9	12,055
	Northern & Yorkshire	17	18	15	13	15	19,096
	Oxford	8	8	6	6	7	8,409
	South West	17	18	16	16	17	21,188
	Thames	27	25	19	19	21	26,658
	Trent	1	2	10	10	8	9,903
	West Midlands	18	14	11	11	12	15,550
**End of follow-up (years from diagnosis)**	≤1	0	1	1	0	0	647
	1-2	1	1	1	1	1	1,028
	2-3	2	1	1	1	1	1,450
	3-4	2	2	1	1	1	1,774
	4-5	2	2	1	97	40	51,548
	5-10	8	7	8	-	5	5,921
	10-15	9	8	87	-	30	38,804
	15-20	11	78	-	-	14	17,394
	20-21	65	-	-	-	8	9,674
**Total percent**		**100**	**100**	**100**	**100**	**100**	
**Total number of women**		**14,981**	**20,190**	**41,272**	**51,797**		**128,240**
**Number of women per year**		**2140**	**4038**	**8254**	**7400**		

(continued on next page)

Age at diagnosis categories reflect eligibility for the breast cancer screening programme, i.e. 50-64 years for all years of diagnosis and 65-70 years from 2005.

For tumour size, number of positive nodes, tumour grade, ER status and breast cancer laterality, some values were unknown in the original data. Values for these characteristics have been estimated using multiple imputation, see Statistical methods for details (text S2).

HER2: human epidermal growth factor receptor 2. Data available only for period 2010-2015. Regions are based on the former cancer registry regions.

Table S3 (continued**)**

b) Not screen-detected cancers in women in age-groups eligible for screening

Characteristic		Percent of women by calendar period of diagnosis 1993-1999	2000-2004	2005-2009	2010-2015	Percent All years	Women All years
**Age at diagnosis (years)**	50-64	100	100	75	72	86	114,936
	65-70	0	0	25	28	14	18,491
**Cancer screen-detected**	Eligible: not screen-detected	100	100	100	100	100	133,427
**Tumour size (mm)**	1-20	61	58	50	50	54	72,778
	21-50	36	38	44	44	41	54,351
	>50	3	4	6	6	5	6,298
**Number of positive nodes**	0	46	53	48	58	52	69,736
	1 to 3	37	31	34	30	33	43,541
	4 to 9	12	11	12	8	10	13,854
	10 or more	5	5	6	4	5	6,296
**Tumour grade**	Low	21	19	13	12	16	21,721
	Medium	44	45	45	49	46	61,173
	High	35	36	42	39	38	50,533
**Oestrogen-receptor status**	Positive	75	77	78	82	78	104,493
	Negative	25	23	22	18	22	28,934
**HER2 status**	Negative	0	0	0	85	24	31,889
	Positive	0	0	0	15	4	5,807
	Before 2010	100	100	100	0	72	95,731
**Breast cancer laterality**	Left	52	52	52	52	52	69,039
	Right	48	48	48	48	48	64,388
**Index of multiple deprivation**	<20% (least deprived)	24	23	23	24	24	31,921
	20-39%	23	23	23	23	23	30,626
	40-59%	20	21	21	20	20	27,206
	60-79%	18	18	18	18	18	23,626
	80+% (most deprived)	15	15	15	15	15	20,048
**Region of residence**	Eastern	10	10	11	12	11	14,211
	North West	21	22	17	13	18	23,847
	Northern & Yorkshire	11	12	12	13	12	16,019
	Oxford	7	5	5	6	6	7,661
	South West	15	13	15	15	15	19,700
	Thames	18	15	21	22	19	25,877
	Trent	7	14	8	9	9	12,441
	West Midlands	11	9	11	10	10	13,671
**End of follow-up (years from diagnosis)**	≤1	3	0	1	1	1	1,880
	1-2	4	3	3	2	3	3,849
	2-3	4	3	3	2	3	4,092
	3-4	4	3	3	2	3	3,996
	4-5	3	3	3	93	28	37,767
	5-10	11	10	11	0	8	10,175
	10-15	9	9	76	0	23	30,521
	15-20	9	69	0	0	18	23,845
	20-21	53	0	0	0	13	17,302
**Total percent**		**100**	**100**	**100**	**100**	**100**	
**Total number of women**		**32,484**	**30,571**	**32,676**	**37,696**		**133,427**
**Number of women per year**		**4641**	**6114**	**6535**	**5385**		

(continued on next page)

Table S3 (continued)

c) Cancers in women who were not in age-groups where routine screening was carried out

Characteristic		Percent of women by calendar period of diagnosis				Percent	Women
		1993-1999	2000-2004	2005-2009	2010-2015	All years	All years
**Age at diagnosis (years)**	18-39	12	12	12	10	12	28,123
	40-49	33	30	41	42	36	91,308
	65-70	20	22	0	0	11	27,784
	71-79	26	26	32	33	29	72,785
	80-89	9	10	15	15	12	30,780
**Cancer screen-detected**	Not eligible for screening	100	100	100	100	100	250,780
**Tumour size (mm)**	1-20	54	52	48	50	51	127,957
	21-50	42	43	46	44	44	109,625
	>50	4	5	6	6	5	13,198
**Number of positive nodes**	0	44	52	48	60	51	128,565
	1 to 3	38	31	34	29	33	82,818
	4 to 9	13	12	12	8	11	27,524
	10 or more	5	5	6	3	5	11,873
**Tumour grade**	Low	19	17	12	13	16	39,056
	Medium	45	47	47	50	47	118,040
	High	36	36	41	37	37	93,684
**Oestrogen-receptor status**	Positive	77	81	81	84	81	202,236
	Negative	23	19	19	16	19	48,544
**HER2 status**	Negative	0	0	0	86	23	57,400
	Positive	0	0	0	14	4	9,445
	Before 2010	100	100	100	0	73	183,935
**Breast cancer laterality**	Left	52	52	51	51	52	129,286
	Right	48	48	49	49	48	121,494
**Index of multiple deprivation**	<20% (least deprived)	22	22	22	23	23	55,501
	20-39%	22	22	23	23	22	56,207
	40-59%	21	21	21	21	21	53,180
	60-79%	19	19	19	18	19	47,137
	80+% (most deprived)	16	16	15	15	15	38,755
**Region of residence**	Eastern	11	12	10	10	10	27,198
	North West	14	14	14	13	14	34,098
	Northern & Yorkshire	12	13	13	13	13	31,815
	Oxford	7	5	5	6	6	14,872
	South West	17	15	16	16	16	40,155
	Thames	22	21	23	22	22	55,076
	Trent	4	9	8	10	8	19,612
	West Midlands	13	11	11	10	11	27,954
**End of follow-up (years from diagnosis)**	<=1	5	4	1	1	4	5,734
	1-2	5	4	4	3	4	10,038
	2-3	5	4	4	3	4	10,432
	3-4	5	4	4	3	4	10,539
	4-5	4	4	3	90	27	67,172
	5-10	17	16	17	0	12	30,744
	10-15	13	14	67	0	21	53,715
	15-20	10	50	0	0	15	38,812
	20-21	36	0	0	0	9	23,594
**Total percent**		**100**	**100**	**100**	**100**	**100**	
**Total number of women**		65,889	64,065	53,981	66,845		250,780
**Number of women per year**		9413	12813	10796	9549		

Breast Cancer Mortality

Table S4: Numbers of women diagnosed with early breast cancer, crude annual breast cancer mortality rates and cumulative breast cancer mortality risks by calendar period of diagnosis, and time since diagnosis (Figure 1, top panel)

Calendar period of diagnosis	Time since diagnosis of early breast cancer (years)	No. women at risk at start of time category	No. breast cancer deaths during time category	Crude annual mortality rate at end of time category, % (95% CI)	Cumulative mortality risk at end of time category, % (95% CI)
1993-1999	0·25-2·5	113,354	7,588	3·84 (3·77 - 3·92)	7·0 (6·9 - 7·2)
	2·5-5	102,305	7,955	2·71 (2·66 - 2·75)	14·4 (14·2 - 14·6)
	5-10	90,814	8,186	1·50 (1·48 - 1·53)	22·5 (22·3 - 22·8)
	10-15	74,583	4,334	1·04 (1·02 - 1·07)	27·2 (27·0 - 27·5)
	15-20	61,799	2,552	0·80 (0·78 - 0·83)	30·5 (30·2 - 30·8)
2000-2004	0·25-2·5	114,826	5,429	2·79 (2·73 - 2·86)	4·9 (4·8 - 5·1)
	2·5-5	106,741	6,255	2·02 (1·98 - 2·06)	10·6 (10·4 - 10·7)
	5-10	97,193	6,786	1·18 (1·16 - 1·21)	17·1 (16·9 - 17·4)
	10-15	82,319	3,807	0·87 (0·83 - 0·90)	21·2 (21·0 - 21·5)
2005-2009	0·25-2·5	127,929	4,218	2·01 (1·96 - 2·06)	3·5 (3·4 - 3·6)
	2·5-5	121,268	5,341	1·52 (1·48 - 1·55)	7·7 (7·6 - 7·9)
	5-10	112,494	6,229	1·00 (0·96 - 1·04)	13·1 (12·9 - 13·3)
2010-2015	0·25-2·5	156,338	3,368	1·26 (1·22 - 1·30)	2·2 (2·2 - 2·3)
	2·5-5	150,702	4,010	0·98 (0·89 - 1·08)	4·9 (4·8 - 5·0)

Table S5: Numbers of women diagnosed with early breast cancer at ages 50-64 years, crude annual breast cancer mortality rates and cumulative breast cancer mortality risks by screening status, calendar period of diagnosis, and time since diagnosis

a) Screen-detected cancers (Figure 1, second row)

Calendar period of diagnosis	Time since diagnosis of early breast cancer (years)	No. women at risk at start of time category	No. breast cancer deaths during time category	Crude annual mortality rate at end of time category, % (95% CI)	Cumulative mortality risk at end of time category, % (95% CI)
1993-1999	0·25-2·5	14,981	301	1·43 (1·30 - 1·55)	2·2 (2·0 - 2·4)
	2·5-5	14,494	493	1·20 (1·13 - 1·28)	5·5 (5·1 - 5·8)
	5-10	13,798	658	0·87 (0·82 - 0·92)	10·1 (9·6 - 10·6)
	10-15	12,595	479	0·75 (0·70 - 0·80)	13·7 (13·1 - 14·2)
	15-20	11,259	368	0·68 (0·61 - 0·75)	16·7 (16·1 - 17·3)
2000-2004	0·25-2·5	20,190	284	0·99 (0·90 - 1·08)	1·5 (1·3 - 1·6)
	2·5-5	19,696	497	0·91 (0·85 - 0·97)	3·9 (3·6 - 4·1)
	5-10	18,915	674	0·69 (0·65 - 0·73)	7·6 (7·2 - 7·9)
	10-15	17,510	563	0·60 (0·54 - 0·66)	10·5 (10·1 - 10·9)
2005-2009	0·25-2·5	29,155	230	0·64 (0·58 - 0·70)	0·9 (0·8 - 1·0)
	2·5-5	28,684	532	0·68 (0·63 - 0·73)	2·6 (2·4 - 2·8)
	5-10	27,801	785	0·55 (0·50 - 0·61)	5·6 (5·3 - 5·8)
2010-2015	0·25-2·5	34,779	145	0·31 (0·27 - 0·35)	0·4 (0·4 - 0·5)
	2·5-5	34,423	306	0·35 (0·23 - 0·47)	1·3 (1·2 - 1·5)

(continued on next page)

Table S5 (continued)

b) Not screen-detected cancers (Figure 1, third row)

Calendar period of diagnosis	Time since diagnosis of early breast cancer (years)	No. women at risk at start of time category	No. breast cancer deaths during time category	Crude annual mortality rate at end of time category, % (95% CI)	Cumulative mortality risk at end of time category, % (95% CI)
1993-1999	0·25-2·5	32,484	2,207	4·03 (3·88 - 4·17)	7·0 (6·8 - 7·3)
	2·5-5	29,653	2,344	2·69 (2·61 - 2·77)	14·6 (14·2 - 14·9)
	5-10	26,746	2,409	1·43 (1·39 - 1·48)	22·4 (22·0 - 22·9)
	10-15	23,068	1,301	1·00 (0·96 - 1·05)	26·9 (26·4 - 27·4)
	15-20	20,163	830	0·78 (0·73 - 0·83)	30·1 (29·6 - 30·6)
2000-2004	0·25-2·5	30,571	1,474	2·90 (2·78 - 3·03)	4·9 (4·7 - 5·2)
	2·5-5	28,700	1,700	2·02 (1·94 - 2·10)	10·7 (10·4 - 11·0)
	5-10	26,589	1,873	1·14 (1·10 - 1·18)	17·1 (16·7 - 17·5)
	10-15	23,606	1,065	0·83 (0·77 - 0·89)	21·0 (20·6 - 21·5)
2005-2009	0·25-2·5	24,586	985	2·60 (2·47 - 2·74)	4·2 (4·0 - 4·4)
	2·5-5	23,324	1,298	1·88 (1·78 - 1·98)	9·5 (9·2 - 9·9)
	5-10	21,694	1,462	1·13 (1·03 - 1·22)	15·7 (15·3 - 16·2)
2010-2015	0·25-2·5	27,295	644	1·49 (1·39 - 1·58)	2·4 (2·2 - 2·6)
	2·5-5	26,394	808	1·13 (0·88 - 1·38)	5·5 (5·2 - 5·7)

Table S6: Numbers of women diagnosed with early breast cancer, crude annual breast cancer mortality rates and cumulative breast cancer mortality risks by ER status, and time since diagnosis (Figure 1, bottom panel).

ER status	Time since diagnosis of early breast cancer (years)	No. women at risk at start of time category	No. breast cancer deaths during time category	Crude annual mortality rate at end of time category, % (95% CI)	Cumulative mortality risk at end of time category, % (95% CI)
Positive	0·25-2·5	421,629	10,909	1·61 (1·51 - 1·71)	2·7 (2·5 - 2·8)
	2·5-5	403,349	15,561	1·58 (1·53 - 1·64)	6·6 (6·4 - 6·8)
	5-10	251,543	16,627	1·25 (1·22 - 1·27)	13·0 (12·8 - 13·2)
	10-15	213,257	7,736	1·03 (1·00 - 1·06)	17·8 (17·6 - 18·0)
	15-20	107,488	2,746	0·89 (0·85 - 0·94)	21·7 (21·5 - 21·8)
Negative	0·25-2·5	90,818	9,694	5·98 (5·64 - 6·33)	11·2 (10·7 - 11·6)
	2·5-5	77,667	8,000	3·24 (3·04 - 3·43)	20·7 (20·2 - 21·2)
	5-10	48,958	4,574	1·26 (1·14 - 1·38)	28·2 (27·7 - 28·8)
	10-15	40,404	1,346	0·71 (0·59 - 0·82)	31·5 (31·0 - 32·1)
	15-20	23,133	403	0·47 (0·36 - 0·57)	33·5 (32·9 - 34·0)

Breast cancer mortality (to accompany figure 1)

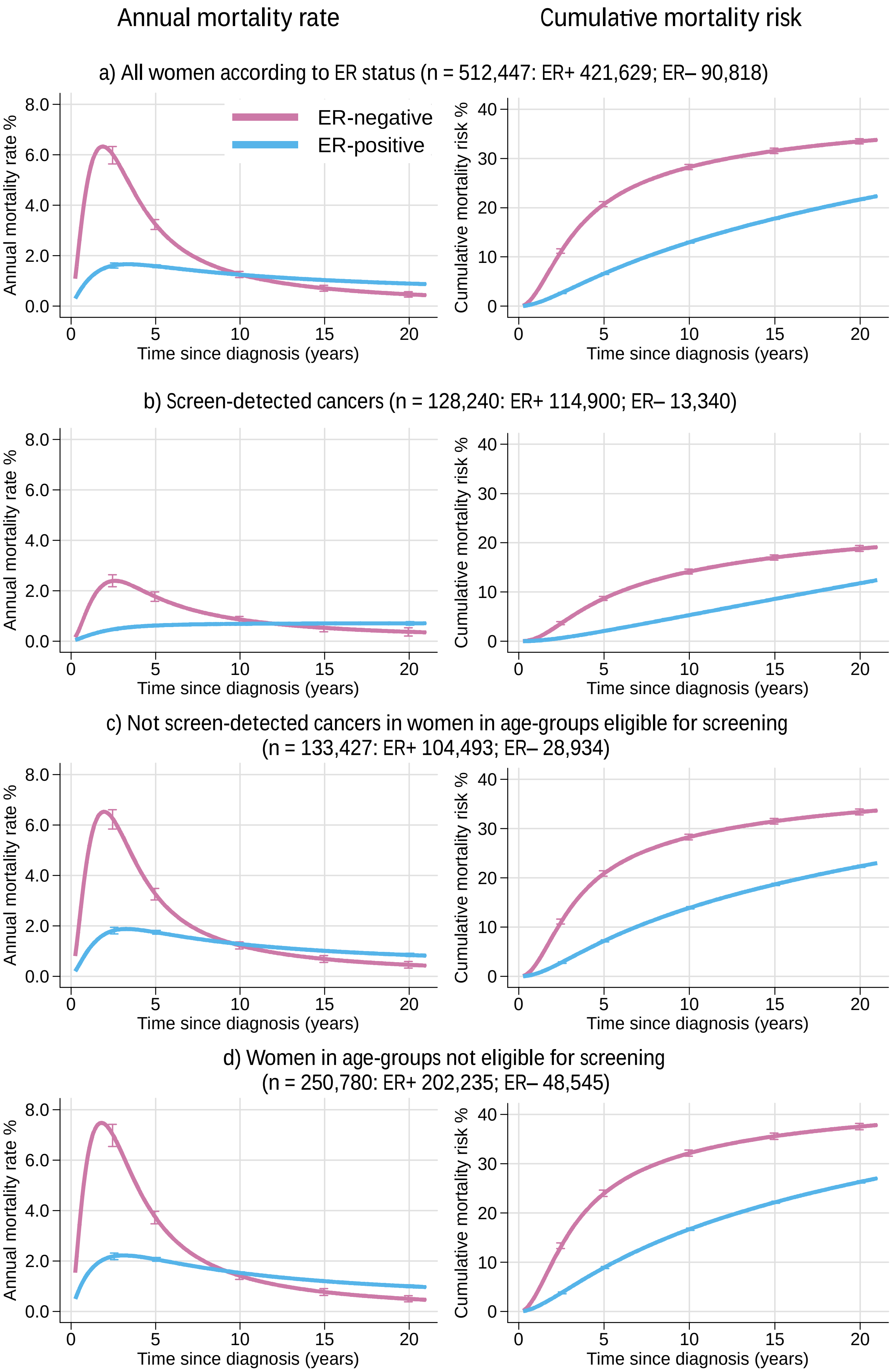

Figure S4: Crude annual breast cancer mortality rates and cumulative breast cancer mortality risks in 512,447 women with early breast cancer by time since diagnosis, according to ER status and screening status

Vertical lines are 95% confidence intervals.

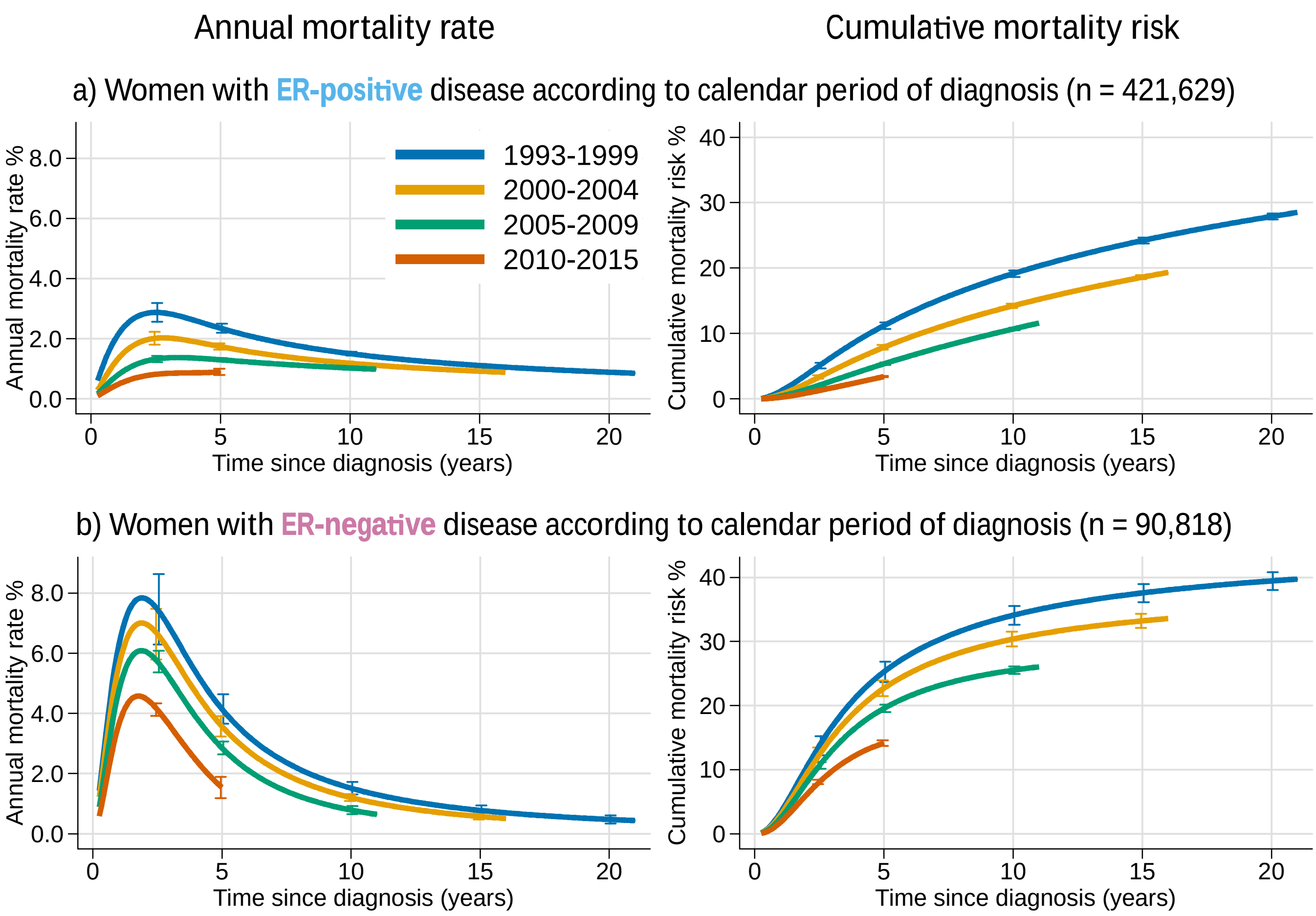

Figure S5: Crude annual breast cancer mortality rates and cumulative breast cancer mortality risks in 512,447 women with early breast cancer with ER-positive or ER-negative disease by time since diagnosis, according to calendar period of diagnosis

Vertical lines are 95% confidence intervals.

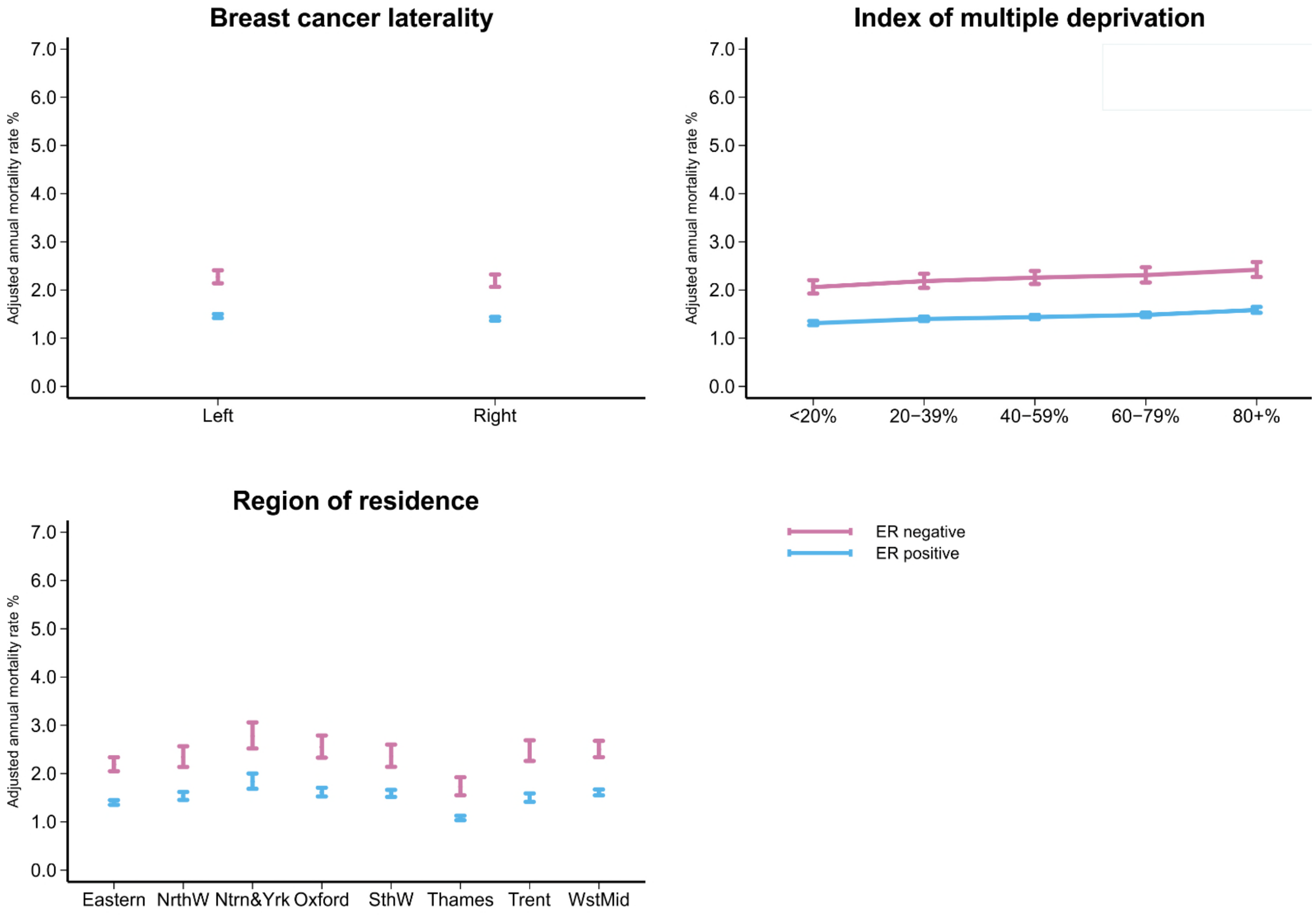

Figure S6: Adjusted annual breast cancer mortality rates in women with early breast cancer with ER-positive or ER-negative disease, according to breast cancer laterality, index of multiple deprivation and region of residence

For each characteristic, rates are adjusted for the other characteristics shown in this figure, for the characteristics shown in figure 2 and also for time since diagnosis.

Further details are in figure S7.

Vertical lines are 95% confidence intervals.

Breast cancer mortality (to accompany figure 2)

ER-positive

Characteristic	Deaths/ Women	Adjusted annual mortality rate % (95% CI)	Rate ratio
**Calendar period of diagnosis ($p=3.1x10^{-37}$)**			
1993- 1999	21,662/86,965	2.06 (1.94, 2.18)	1.00
2000- 2004	16,343/93,781	1.52 (1.46, 1.59)	0.74
2005- 2009	11,507/106,484	1.09 (1.06, 1.14)	0.53
2010- 2015	4,398/134,397	0.70 (0.67, 0.73)	0.34
**Age at diagnosis (years) ($p=3.9x10^{-76}$)**			
18- 39	4,202/20,157	1.50 (1.42, 1.58)	1.00
40- 49	9,645/73,435	1.13 (1.08, 1.18)	0.75
50- 64	18,865/177,712	1.28 (1.22, 1.34)	0.86
65- 70	7,275/64,589	1.58 (1.52, 1.64)	1.06
71- 79	9,718/60,749	1.89 (1.81, 1.97)	1.26
80- 89	4,203/24,984	2.76 (2.63, 2.90)	1.85
**Cancer screen- detected ($p=2.x10^{-115}$)**			
Eligible: screen- detected	5,923/114,900	1.01 (0.96, 1.06)	1.00
Eligible: not screen- detected	15,126/104,493	1.53 (1.46, 1.60)	1.51
Not eligible for screening	32,861/202,235	1.49 (1.44, 1.54)	1.47
**Tumour size (mm) ($p=4.x10^{-143}$)**			
1- 20	20,502/257,689	1.03 (1.00, 1.06)	1.00
21- 50	28,765/147,684	1.78 (1.73, 1.83)	1.74
>50	4,643/16,254	2.40 (2.30, 2.52)	2.34
**Number of positive nodes ($p=9.x10^{-125}$)**			
0	15,489/242,061	0.76 (0.74, 0.79)	1.00
1 to 3	20,995/127,078	1.69 (1.64, 1.75)	2.22
4 to 9	11,285/37,339	3.00 (2.90, 3.11)	3.93
10 or more	6,141/15,149	4.53 (4.35, 4.73)	5.94
**Tumour grade ($p=4.3x10^{-69}$)**			
Low	5,313/93,798	0.71 (0.68, 0.73)	1.00
Medium	27,152/223,802	1.29 (1.27, 1.32)	1.83
High	21,445/104,027	1.95 (1.87, 2.03)	2.76
**Breast cancer laterality ($p=1.3x10^{-04}$)**			
Left	27,983/215,990	1.46 (1.42, 1.50)	1.00
Right	25,929/205,638	1.40 (1.36, 1.44)	0.96
**Index of multiple deprivation ($p=3.9x10^{-27}$)**			
<20% (least deprived)	11,404/98,369	1.31 (1.27, 1.36)	1.00
20- 39%	12,061/97,503	1.40 (1.36, 1.45)	1.07
40- 59%	11,400/89,098	1.44 (1.39, 1.48)	1.10
60- 79%	10,429/76,540	1.48 (1.44, 1.53)	1.13
80+% (most deprived)	8,616/60,117	1.59 (1.53, 1.65)	1.21
**Region of residence ($p=3.7x10^{-57}$)**			
Eastern	5,762/47,477	1.40 (1.35, 1.45)	1.00
North West	7,690/57,846	1.53 (1.45, 1.62)	1.10
Northern & Yorkshire	6,390/52,807	1.84 (1.68, 2.00)	1.31
Oxford	3,010/25,492	1.61 (1.52, 1.71)	1.15
South West	8,849/68,253	1.59 (1.52, 1.66)	1.13
Thames	11,983/88,463	1.08 (1.03, 1.13)	0.77
Trent	3,847/34,691	1.50 (1.41, 1.59)	1.07
West Midlands	6,378/46,597	1.61 (1.55, 1.67)	1.15
**Time since diagnosis (years) ($p=4.6x10^{-08}$)**			
<=1	2,195/421,628	0.74 (0.68, 0.80)	1.00
1- 2	5,445/416,990	1.43 (1.33, 1.54)	1.94
2- 3	6,467/408,456	1.81 (1.71, 1.91)	2.46
3- 4	6,767/398,301	2.02 (1.92, 2.12)	2.74
4- 5	5,594/386,920	1.77 (1.70, 1.85)	2.41
5- 10	16,626/251,543	1.52 (1.48, 1.56)	2.06
10- 15	7,736/213,256	1.21 (1.16, 1.26)	1.64
15- 20	2,746/107,488	0.96 (0.90, 1.03)	1.31
20- 21	332/40,078	0.82 (0.71, 0.94)	1.11
**All ER- positive**	53,912/421,628	1.43 (1.39, 1.47)	

Axis: 0 2 4 6

ER-negative

Characteristic	Deaths/ Women	Adjusted annual mortality rate % (95% CI)	Rate ratio
**Calendar period of diagnosis ($p=6.4x10^{-15}$)**			
1993- 1999	9,331/26,389	2.94 (2.62, 3.29)	1.00
2000- 2004	6,530/21,044	2.48 (2.29, 2.68)	0.84
2005- 2009	5,221/21,444	1.88 (1.77, 2.00)	0.64
2010- 2015	2,979/21,940	1.57 (1.48, 1.66)	0.53
**Age at diagnosis (years) ($p=1.6x10^{-36}$)**			
18- 39	2,411/7,965	2.02 (1.87, 2.19)	1.00
40- 49	4,691/17,872	1.74 (1.62, 1.88)	0.86
50- 64	8,660/36,329	2.02 (1.84, 2.20)	1.00
65- 70	2,682/10,820	2.40 (2.21, 2.60)	1.18
71- 79	3,737/12,035	2.91 (2.67, 3.18)	1.44
80- 89	1,880/5,795	4.11 (3.69, 4.57)	2.03
**Cancer screen- detected ($p=4.8x10^{-26}$)**			
Eligible: screen- detected	1,762/13,339	1.61 (1.47, 1.77)	1.00
Eligible: not screen- detected	7,870/28,933	2.34 (2.16, 2.54)	1.45
Not eligible for screening	14,430/48,544	2.35 (2.18, 2.53)	1.45
**Tumour size (mm) ($p=5.2x10^{-58}$)**			
1- 20	7,138/42,849	1.63 (1.52, 1.75)	1.00
21- 50	14,217/42,575	2.68 (2.53, 2.84)	1.65
>50	2,707/5,393	3.75 (3.48, 4.05)	2.31
**Number of positive nodes ($p=2.8x10^{-85}$)**			
0	6,716/46,397	1.21 (1.12, 1.30)	1.00
1 to 3	9,049/28,585	2.66 (2.48, 2.84)	2.20
4 to 9	5,037/10,385	4.49 (4.18, 4.83)	3.72
10 or more	3,259/5,448	6.37 (5.92, 6.84)	5.28
**Tumour grade ($p=1.2x10^{-36}$)**			
Low	370/3,660	1.03 (0.85, 1.25)	1.00
Medium	4,534/21,380	2.05 (1.91, 2.21)	1.99
High	19,158/65,777	2.98 (2.85, 3.12)	2.88
**Breast cancer laterality ($p=2.9x10^{-02}$)**			
Left	12,876/47,916	2.27 (2.14, 2.41)	1.00
Right	11,186/42,901	2.19 (2.06, 2.32)	0.96
**Index of multiple deprivation ($p=3.7x10^{-09}$)**			
<20% (least deprived)	4,812/19,678	2.06 (1.93, 2.21)	1.00
20- 39%	5,020/19,562	2.19 (2.04, 2.34)	1.06
40- 59%	5,025/18,710	2.26 (2.13, 2.40)	1.09
60- 79%	4,657/16,996	2.31 (2.16, 2.47)	1.12
80+% (most deprived)	4,546/15,869	2.42 (2.27, 2.58)	1.17
**Region of residence ($p=9.2x10^{-22}$)**			
Eastern	2,413/9,312	2.19 (2.05, 2.34)	1.00
North West	3,185/12,153	2.34 (2.14, 2.57)	1.07
Northern & Yorkshire	3,702/14,122	2.78 (2.52, 3.06)	1.27
Oxford	1,441/5,449	2.55 (2.33, 2.79)	1.16
South West	3,157/12,789	2.36 (2.14, 2.60)	1.08
Thames	5,301/19,147	1.73 (1.55, 1.92)	0.79
Trent	1,868/7,264	2.47 (2.26, 2.69)	1.13
West Midlands	2,991/10,577	2.50 (2.34, 2.68)	1.14
**Time since diagnosis (years) ($p=3.1x10^{-31}$)**			
<=1	2,171/90,818	2.07 (1.91, 2.24)	1.00
1- 2	5,045/87,195	4.05 (3.77, 4.36)	1.96
2- 3	4,531/80,814	4.22 (3.93, 4.53)	2.04
3- 4	3,670/74,995	3.87 (3.59, 4.17)	1.87
4- 5	2,274/70,067	2.65 (2.44, 2.87)	1.28
5- 10	4,574/48,957	1.60 (1.47, 1.74)	0.77
10- 15	1,345/40,404	0.76 (0.67, 0.87)	0.37
15- 20	402/23,132	0.45 (0.34, 0.59)	0.22
20- 21	46/10,491	0.34 (0.20, 0.56)	0.16
**All ER- negative**	24,062/90,818	2.23 (2.11, 2.36)	

Axis: 0 2 4 6

Figure S7: Adjusted annual breast cancer mortality rates in women with early breast cancer with ER-positive or ER-negative disease according to nine characteristics, and time since diagnosis

For each characteristic, rates are adjusted for all the other characteristics shown including time since diagnosis.

ER-positive (years 0-5)

Characteristic	Deaths/ Women	Adjusted annual mortality rate % (95% CI)	Rate ratio
**Calendar period of diagnosis ($p=1.8x10^{-38}$)**			
1993- 1999	9,358/86,965	2.64 (2.43, 2.87)	1.00
2000- 2004	7,151/93,781	1.83 (1.70, 1.97)	0.69
2005- 2009	5,561/106,484	1.21 (1.15, 1.28)	0.46
2010- 2015	4,398/134,397	0.86 (0.83, 0.88)	0.32
**Age at diagnosis (years) ($p=1.7x10^{-60}$)**			
18- 39	1,952/20,157	1.51 (1.40, 1.62)	1.00
40- 49	4,211/73,435	1.11 (1.05, 1.18)	0.74
50- 64	8,209/177,712	1.40 (1.33, 1.47)	0.93
65- 70	3,587/64,589	1.73 (1.64, 1.82)	1.15
71- 79	5,422/60,749	2.01 (1.92, 2.11)	1.34
80- 89	3,086/24,984	3.05 (2.91, 3.20)	2.03
**Cancer screen- detected ($p=8.2x10^{-90}$)**			
Screen- detected	2,310/114,900	0.96 (0.90, 1.01)	1.00
Not screen- detected	24,159/306,728	1.66 (1.59, 1.73)	1.74
**Tumour size (mm) ($p=2.x10^{-127}$)**			
1- 20	8,252/257,689	1.05 (1.00, 1.10)	1.00
21- 50	15,248/147,684	1.88 (1.81, 1.96)	1.80
>50	2,969/16,254	2.68 (2.53, 2.84)	2.56
**Number of positive nodes ($p=7.x10^{-130}$)**			
0	6,401/242,061	0.75 (0.71, 0.79)	1.00
1 to 3	9,971/127,078	1.74 (1.65, 1.83)	2.32
4 to 9	6,242/37,339	3.24 (3.10, 3.39)	4.34
10 or more	3,854/15,149	5.04 (4.80, 5.30)	6.75
**Tumour grade ($p=1.9x10^{-83}$)**			
Low	1,512/93,798	0.50 (0.47, 0.53)	1.00
Medium	11,513/223,802	1.17 (1.13, 1.21)	2.34
High	13,444/104,027	2.48 (2.35, 2.61)	4.97
**Breast cancer laterality ($p=4.4x10^{-03}$)**			
Left	13,807/215,990	1.60 (1.53, 1.67)	1.00
Right	12,662/205,638	1.53 (1.47, 1.60)	0.96
**Index of multiple deprivation ($p=2.6x10^{-22}$)**			
<20% (least deprived)	5,349/98,369	1.41 (1.34, 1.48)	1.00
20- 39%	5,777/97,503	1.51 (1.44, 1.59)	1.07
40- 59%	5,607/89,098	1.57 (1.50, 1.65)	1.12
60- 79%	5,205/76,540	1.62 (1.55, 1.70)	1.15
80+% (most deprived)	4,530/60,117	1.80 (1.70, 1.90)	1.28
**Region of residence ($p=3.9x10^{-26}$)**			
Eastern	2,663/47,477	1.46 (1.39, 1.53)	1.00
North West	3,914/57,846	1.74 (1.62, 1.88)	1.19
Northern & Yorkshire	3,039/52,807	1.97 (1.73, 2.23)	1.35
Oxford	1,447/25,492	1.78 (1.63, 1.95)	1.22
South West	4,481/68,253	1.79 (1.65, 1.94)	1.23
Thames	5,935/88,463	1.18 (1.09, 1.28)	0.81
Trent	1,911/34,691	1.63 (1.51, 1.76)	1.12
West Midlands	3,075/46,597	1.73 (1.64, 1.81)	1.18
**Time since diagnosis (years) ($p=1.6x10^{-69}$)**			
<=1	2,195/421,628	0.73 (0.68, 0.78)	1.00
1- 2	5,445/416,990	1.43 (1.34, 1.52)	1.96
2- 3	6,467/408,456	1.82 (1.74, 1.91)	2.50
3- 4	6,767/398,301	2.05 (1.96, 2.14)	2.81
4- 5	5,594/386,920	1.81 (1.75, 1.88)	2.49
**All ER- positive**	26,470/421,628	1.57 (1.51, 1.63)	

ER-negative (years 0-5)

Characteristic	Deaths/ Women	Adjusted annual mortality rate % (95% CI)	Rate ratio
**Calendar period of diagnosis ($p=9.5x10^{-15}$)**			
1993- 1999	6,184/26,389	4.34 (3.81, 4.93)	1.00
2000- 2004	4,532/21,044	3.67 (3.36, 4.00)	0.85
2005- 2009	3,997/21,444	2.86 (2.67, 3.05)	0.66
2010- 2015	2,979/21,940	2.31 (2.20, 2.42)	0.53
**Age at diagnosis (years) ($p=9.2x10^{-44}$)**			
18- 39	1,708/7,965	2.84 (2.63, 3.07)	1.00
40- 49	3,235/17,872	2.44 (2.29, 2.60)	0.86
50- 64	6,038/36,329	2.87 (2.71, 3.05)	1.01
65- 70	2,023/10,820	3.45 (3.17, 3.74)	1.21
71- 79	2,980/12,035	4.10 (3.78, 4.45)	1.44
80- 89	1,707/5,795	5.90 (5.37, 6.48)	2.08
**Cancer screen- detected ($p=3.9x10^{-33}$)**			
Screen- detected	1,117/13,339	2.11 (1.94, 2.31)	1.00
Not screen- detected	16,576/77,478	3.40 (3.20, 3.60)	1.61
**Tumour size (mm) ($p=8.6x10^{-64}$)**			
1- 20	4,669/42,849	2.23 (2.07, 2.39)	1.00
21- 50	10,764/42,575	3.82 (3.60, 4.05)	1.72
>50	2,259/5,393	5.49 (5.07, 5.95)	2.47
**Number of positive nodes ($p=5.0x10^{-97}$)**			
0	4,611/46,397	1.68 (1.55, 1.81)	1.00
1 to 3	6,479/28,585	3.69 (3.47, 3.94)	2.20
4 to 9	3,920/10,385	6.33 (5.90, 6.79)	3.78
10 or more	2,682/5,448	8.93 (8.30, 9.62)	5.33
**Tumour grade ($p=4.7x10^{-48}$)**			
Low	151/3,660	1.05 (0.82, 1.34)	1.00
Medium	2,679/21,380	2.65 (2.44, 2.88)	2.54
High	14,862/65,777	4.73 (4.52, 4.95)	4.52
**Breast cancer laterality ($p=1.4x10^{-02}$)**			
Left	9,511/47,916	3.28 (3.09, 3.48)	1.00
Right	8,182/42,901	3.13 (2.95, 3.33)	0.96
**Index of multiple deprivation ($p=4.3x10^{-10}$)**			
<20% (least deprived)	3,475/19,678	2.96 (2.76, 3.17)	1.00
20- 39%	3,628/19,562	3.10 (2.89, 3.33)	1.05
40- 59%	3,680/18,710	3.24 (3.05, 3.44)	1.10
60- 79%	3,461/16,996	3.32 (3.09, 3.57)	1.12
80+% (most deprived)	3,447/15,869	3.56 (3.33, 3.79)	1.20
**Region of residence ($p=6.9x10^{-15}$)**			
Eastern	1,769/9,312	3.17 (2.97, 3.40)	1.00
North West	2,377/12,153	3.35 (3.04, 3.69)	1.06
Northern & Yorkshire	2,628/14,122	3.90 (3.48, 4.36)	1.23
Oxford	1,025/5,449	3.66 (3.27, 4.08)	1.15
South West	2,395/12,789	3.43 (3.08, 3.81)	1.08
Thames	3,837/19,147	2.47 (2.16, 2.82)	0.78
Trent	1,392/7,264	3.49 (3.20, 3.80)	1.10
West Midlands	2,268/10,577	3.70 (3.47, 3.94)	1.17
**Time since diagnosis (years) ($p=5.4x10^{-08}$)**			
<=1	2,171/90,818	1.92 (1.78, 2.07)	1.00
1- 2	5,045/87,195	3.77 (3.53, 4.03)	1.97
2- 3	4,531/80,814	3.95 (3.70, 4.21)	2.06
3- 4	3,670/74,995	3.64 (3.40, 3.89)	1.89
4- 5	2,274/70,067	2.49 (2.32, 2.68)	1.30
**All ER- negative**	17,693/90,818	3.21 (3.03, 3.40)	

Figure S8: Adjusted annual breast cancer mortality rates during 0-5 years after diagnosis in women with early breast cancer with ER-positive or ER-negative disease according to nine characteristics, and time since diagnosis

For each characteristic, rates are adjusted for all the other characteristics shown including time since diagnosis.

ER-positive (years 5-15)

Characteristic	Deaths/ Women	Annual adjusted mortality rate % (95% CI)	Rate ratio
**Calendar period of diagnosis (p=9.9x10^{-33})**			
1993- 1999	9,759/72,941	1.72 (1.65, 1.80)	1.00
2000- 2004	8,657/82,030	1.32 (1.28, 1.37)	0.77
2005- 2009	5,945/96,570	1.07 (1.03, 1.11)	0.62
**Age at diagnosis (years) (p=1.2x10^{-40})**			
18- 39	1,983/13,190	1.57 (1.49, 1.66)	1.00
40- 49	4,741/45,263	1.17 (1.13, 1.21)	0.74
50- 64	9,213/113,700	1.26 (1.22, 1.30)	0.80
65- 70	3,307/35,360	1.50 (1.44, 1.57)	0.96
71- 79	3,998/33,383	1.76 (1.69, 1.83)	1.12
80- 89	1,117/10,643	2.30 (2.15, 2.46)	1.46
**Cancer screen- detected (p=1.2x10^{-47})**			
Screen- detected	3,147/64,063	1.03 (0.98, 1.08)	1.00
Not screen- detected	21,215/187,479	1.45 (1.41, 1.48)	1.40
**Tumour size (mm) (p=8.x10^{-124})**			
1- 20	10,510/161,131	1.04 (1.01, 1.07)	1.00
21- 50	12,289/82,798	1.77 (1.72, 1.81)	1.70
>50	1,562/7,613	2.14 (2.00, 2.28)	2.06
**Number of positive nodes (p=6.x10^{-129})**			
0	7,831/142,469	0.80 (0.77, 0.82)	1.00
1 to 3	9,767/78,958	1.72 (1.66, 1.78)	2.16
4 to 9	4,610/22,284	2.86 (2.74, 2.99)	3.60
10 or more	2,153/7,831	4.07 (3.82, 4.35)	5.12
**Tumour grade (p=5.5x10^{-48})**			
Low	3,068/63,056	0.82 (0.79, 0.86)	1.00
Medium	13,849/131,453	1.46 (1.43, 1.49)	1.78
High	7,444/57,032	1.56 (1.50, 1.63)	1.90
**Breast cancer laterality (p=6.6x10^{-03})**			
Left	12,614/128,686	1.41 (1.37, 1.44)	1.00
Right	11,748/122,856	1.35 (1.32, 1.39)	0.96
**Index of multiple deprivation (p=3.0x10^{-09})**			
<20% (least deprived)	5,337/59,342	1.28 (1.24, 1.33)	1.00
20- 39%	5,581/58,503	1.37 (1.33, 1.42)	1.07
40- 59%	5,129/53,142	1.38 (1.34, 1.43)	1.08
60- 79%	4,652/45,478	1.43 (1.38, 1.48)	1.11
80+% (most deprived)	3,661/35,075	1.47 (1.41, 1.54)	1.15
**Region of residence (p=4.1x10^{-60})**			
Eastern	2,740/28,071	1.41 (1.35, 1.47)	1.00
North West	3,355/34,822	1.44 (1.36, 1.51)	1.02
Northern & Yorkshire	2,959/31,618	1.78 (1.65, 1.92)	1.26
Oxford	1,390/15,452	1.53 (1.43, 1.64)	1.09
South West	3,812/40,912	1.47 (1.41, 1.53)	1.04
Thames	5,431/52,647	1.05 (1.01, 1.09)	0.74
Trent	1,773/19,370	1.47 (1.39, 1.57)	1.05
West Midlands	2,899/28,647	1.56 (1.50, 1.64)	1.11
**Time since diagnosis (years) (p=4.5x10^{-27})**			
5- 10	16,626/251,543	1.49 (1.45, 1.53)	1.00
10- 15	7,736/213,256	1.20 (1.16, 1.24)	0.81
**All ER- positive**	24,362/251,543	1.38 (1.35, 1.41)	

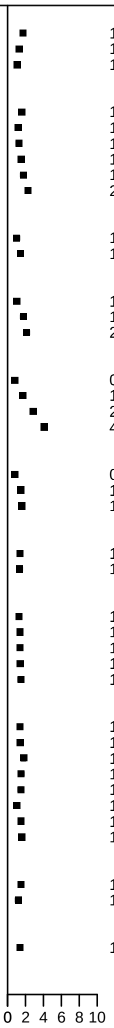

ER-negative (years 5-15)

Characteristic	Deaths/ Women	Adjusted annual mortality rate % (95% CI)	Rate ratio
**Calendar period of diagnosis (p=5.3x10^{-09})**			
1993- 1999	2,760/17,872	1.81 (1.58, 2.08)	1.00
2000- 2004	1,935/15,162	1.51 (1.36, 1.69)	0.83
2005- 2009	1,224/15,923	1.06 (0.95, 1.17)	0.58
**Age at diagnosis (years) (p=6.6x10^{-08})**			
18- 39	654/4,627	1.44 (1.27, 1.65)	1.00
40- 49	1,331/10,653	1.23 (1.12, 1.36)	0.85
50- 64	2,409/21,842	1.33 (1.21, 1.46)	0.92
65- 70	622/5,247	1.58 (1.37, 1.83)	1.10
71- 79	730/5,057	2.01 (1.76, 2.29)	1.39
80- 89	172/1,530	2.35 (1.88, 2.94)	1.63
**Cancer screen- detected (p=7.3x10^{-03})**			
Screen- detected	586/7,729	1.26 (1.10, 1.45)	1.00
Not screen- detected	5,333/41,228	1.49 (1.37, 1.63)	1.19
**Tumour size (mm) (p=3.0x10^{-24})**			
1- 20	2,254/26,164	1.17 (1.06, 1.29)	1.00
21- 50	3,239/20,844	1.78 (1.63, 1.94)	1.52
>50	426/1,949	2.15 (1.85, 2.50)	1.83
**Number of positive nodes (p=7.9x10^{-69})**			
0	1,938/27,004	0.84 (0.76, 0.93)	1.00
1 to 3	2,374/15,497	1.86 (1.68, 2.06)	2.21
4 to 9	1,052/4,566	3.01 (2.66, 3.40)	3.58
10 or more	554/1,890	4.21 (3.66, 4.84)	5.01
**Tumour grade (p=1.1x10^{-03})**			
Low	177/2,848	0.85 (0.67, 1.07)	1.00
Medium	1,664/13,301	1.61 (1.45, 1.77)	1.90
High	4,078/32,808	1.58 (1.48, 1.70)	1.87
**Breast cancer laterality (p=9.8x10^{-01})**			
Left	3,114/25,730	1.46 (1.33, 1.60)	1.00
Right	2,805/23,227	1.46 (1.33, 1.59)	1.00
**Index of multiple deprivation (p=1.0x10^{-01})**			
<20% (least deprived)	1,240/10,990	1.36 (1.22, 1.51)	1.00
20- 39%	1,293/10,727	1.48 (1.34, 1.63)	1.09
40- 59%	1,251/10,114	1.49 (1.34, 1.65)	1.10
60- 79%	1,118/8,975	1.51 (1.34, 1.69)	1.11
80+% (most deprived)	1,016/8,150	1.48 (1.32, 1.65)	1.09
**Region of residence (p=1.8x10^{-10})**			
Eastern	596/5,090	1.41 (1.25, 1.57)	1.00
North West	751/6,504	1.54 (1.34, 1.77)	1.10
Northern & Yorkshire	985/8,024	1.90 (1.64, 2.22)	1.36
Oxford	383/2,993	1.68 (1.44, 1.96)	1.19
South West	728/6,948	1.49 (1.30, 1.71)	1.06
Thames	1,347/10,024	1.15 (1.02, 1.29)	0.82
Trent	454/3,561	1.71 (1.47, 1.99)	1.22
West Midlands	672/5,810	1.52 (1.35, 1.71)	1.08
**Time since diagnosis (years) (p=4.8x10^{-32})**			
5- 10	4,574/48,957	1.82 (1.67, 1.99)	1.00
10- 15	1,345/40,404	0.84 (0.75, 0.95)	0.46
**All ER- negative**	5,920/48,957	1.46 (1.34, 1.58)	

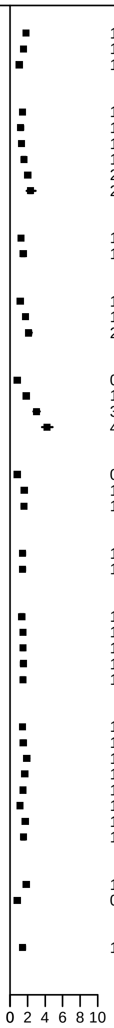

Figure S9: Adjusted annual breast cancer mortality rates during 5-15 years after diagnosis in women with early breast cancer with ER-positive or ER-negative disease according to nine characteristics, and time since diagnosis

For each characteristic, rates are adjusted for all the other characteristics shown including time since diagnosis.

ER-positive (years 15+)

Characteristic	Deaths/ Women	Adjusted annual mortality rate % (95% CI)	Rate ratio
**Calendar period of diagnosis (p=2.4x10^{-01})**			
1993- 1999	2,543/49,655	0.97 (0.91, 1.03)	1.00
2000- 2004	535/57,832	0.91 (0.83, 1.00)	0.94
**Age at diagnosis (years) (p=2.4x10^{-07})**			
18- 39	266/7,066	1.07 (0.93, 1.23)	1.00
40- 49	691/23,192	0.83 (0.76, 0.92)	0.78
50- 64	1,442/56,736	0.90 (0.84, 0.97)	0.84
65- 70	380/12,084	1.18 (1.06, 1.31)	1.10
71- 79	297/8,409	1.54 (1.36, 1.75)	1.44
**Cancer screen- detected (p=3.5x10^{-02})**			
Screen- detected	465/23,739	0.85 (0.76, 0.96)	1.00
Not screen- detected	2,613/83,748	0.98 (0.92, 1.04)	1.15
**Tumour size (mm) (p=6.2x10^{-19})**			
1- 20	1,739/75,301	0.79 (0.74, 0.85)	1.00
21- 50	1,228/29,979	1.26 (1.17, 1.37)	1.59
>50	111/2,206	1.39 (1.09, 1.78)	1.76
**Number of positive nodes (p=9.7x10^{-36})**			
0	1,256/64,176	0.65 (0.59, 0.70)	1.00
1 to 3	1,256/32,774	1.25 (1.16, 1.36)	1.94
4 to 9	432/8,201	1.85 (1.62, 2.11)	2.86
10 or more	133/2,335	2.20 (1.72, 2.82)	3.41
**Tumour grade (p=1.1x10^{-02})**			
Low	732/31,910	0.89 (0.82, 0.98)	1.00
Medium	1,790/52,891	1.17 (1.10, 1.24)	1.30
High	556/22,685	0.72 (0.63, 0.81)	0.80
**Breast cancer laterality (p=5.9x10^{-01})**			
Left	1,561/54,499	0.97 (0.90, 1.04)	1.00
Right	1,517/52,988	0.95 (0.89, 1.01)	0.98
**Index of multiple deprivation (p=2.5x10^{-02})**			
<20% (least deprived)	716/26,853	0.90 (0.83, 0.99)	1.00
20- 39%	702/25,441	0.92 (0.85, 1.01)	1.02
40- 59%	663/22,477	0.99 (0.91, 1.08)	1.10
60- 79%	571/18,701	1.00 (0.91, 1.11)	1.11
80+% (most deprived)	424/14,014	1.01 (0.90, 1.13)	1.12
**Region of residence (p=3.1x10^{-09})**			
Eastern	358/12,350	0.92 (0.82, 1.03)	1.00
North West	419/14,742	0.96 (0.84, 1.10)	1.05
Northern & Yorkshire	391/13,246	1.28 (1.09, 1.50)	1.40
Oxford	172/6,936	0.94 (0.78, 1.14)	1.03
South West	555/17,771	1.11 (0.99, 1.24)	1.21
Thames	615/22,374	0.73 (0.64, 0.83)	0.79
Trent	161/7,447	0.91 (0.76, 1.08)	0.99
West Midlands	403/12,618	1.13 (1.01, 1.26)	1.23
**Time since diagnosis (years) (p=7.0x10^{-02})**			
15- 20	2,746/107,488	0.97 (0.92, 1.03)	1.00
20- 21	332/40,078	0.87 (0.77, 0.98)	0.89
**All ER- positive**	3,078/107,488	0.96 (0.91, 1.01)	

ER-negative (years 15+)

Characteristic	Deaths/ Women	Adjusted annual mortality rate % (95% CI)	Rate ratio
**Calendar period of diagnosis (p=8.5x10^{-01})**			
1993- 1999	387/12,143	0.60 (0.43, 0.83)	1.00
2000- 2004	61/10,989	0.62 (0.43, 0.90)	1.04
**Age at diagnosis (years) (p=1.2x10^{-01})**			
18- 39	48/2,585	0.58 (0.35, 0.97)	1.00
40- 49	125/6,095	0.52 (0.36, 0.74)	0.89
50- 64	212/11,478	0.58 (0.42, 0.79)	0.99
65- 70	36/1,836	0.72 (0.43, 1.19)	1.23
71- 79	26/1,136	1.05 (0.57, 1.92)	1.80
**Cancer screen- detected (p=8.8x10^{-01})**			
Screen- detected	58/3,328	0.59 (0.36, 0.96)	1.00
Not screen- detected	390/19,804	0.60 (0.45, 0.81)	1.03
**Tumour size (mm) (p=6.4x10^{-03})**			
1- 20	214/13,491	0.52 (0.38, 0.71)	1.00
21- 50	212/8,969	0.75 (0.54, 1.05)	1.45
>50	21/671	0.85 (0.44, 1.66)	1.64
**Number of positive nodes (p=2.1x10^{-07})**			
0	166/13,422	0.42 (0.31, 0.57)	1.00
1 to 3	195/7,347	0.78 (0.55, 1.11)	1.88
4 to 9	64/1,790	1.15 (0.71, 1.85)	2.76
10 or more	22/572	1.27 (0.64, 2.53)	3.05
**Tumour grade (p=2.4x10^{-02})**			
Low	41/1,913	0.57 (0.34, 0.95)	1.00
Medium	190/6,826	0.73 (0.53, 1.01)	1.29
High	217/14,393	0.44 (0.34, 0.56)	0.77
**Breast cancer laterality (p=5.6x10^{-01})**			
Left	250/12,299	0.62 (0.46, 0.84)	1.00
Right	198/10,833	0.58 (0.42, 0.80)	0.93
**Index of multiple deprivation (p=3.8x10^{-01})**			
<20% (least deprived)	97/5,523	0.55 (0.37, 0.82)	1.00
20- 39%	98/5,108	0.61 (0.43, 0.88)	1.11
40- 59%	93/4,811	0.58 (0.39, 0.84)	1.04
60- 79%	77/3,981	0.63 (0.42, 0.95)	1.14
80+% (most deprived)	82/3,707	0.67 (0.46, 0.98)	1.21
**Region of residence (p=3.9x10^{-01})**			
Eastern	47/2,474	0.57 (0.38, 0.87)	1.00
North West	57/2,956	0.74 (0.48, 1.14)	1.28
Northern & Yorkshire	88/3,797	0.84 (0.54, 1.29)	1.46
Oxford	32/1,800	0.57 (0.33, 0.99)	0.99
South West	33/2,960	0.56 (0.28, 1.11)	0.98
Thames	117/4,593	0.47 (0.33, 0.66)	0.82
Trent	22/1,735	0.65 (0.35, 1.21)	1.13
West Midlands	50/2,814	0.63 (0.41, 0.95)	1.09
**Time since diagnosis (years) (p=1.8x10^{-01})**			
15- 20	402/23,132	0.62 (0.47, 0.82)	1.00
20- 21	46/10,491	0.47 (0.29, 0.76)	0.76
**All ER- negative**	449/23,132	0.60 (0.45, 0.80)	

Figure S10: Adjusted annual breast cancer mortality rates during 15+ years after diagnosis in women with early breast cancer with ER-positive or ER-negative disease according to nine characteristics, and time since diagnosis

For each characteristic, rates are adjusted for all the other characteristics shown including time since diagnosis.

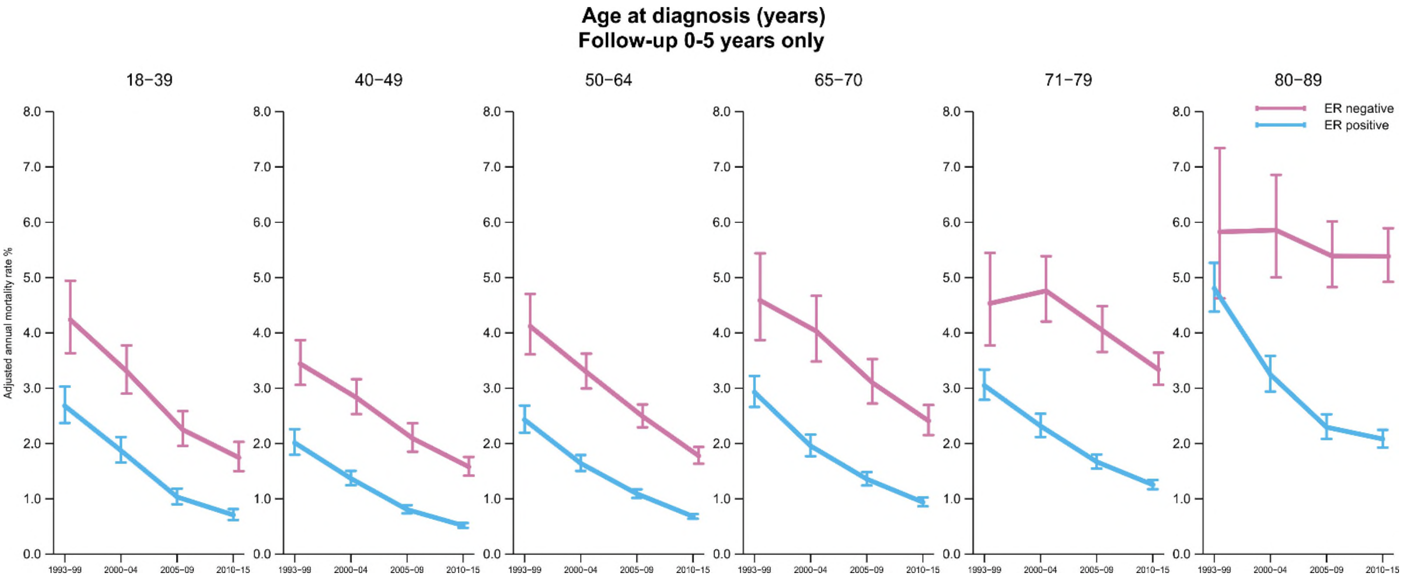

Figure S11: Adjusted annual breast cancer mortality rates during 0-5 years after diagnosis in women with early breast cancer with ER-positive or ER-negative disease by calendar period of diagnosis, according to age at diagnosis

In this figure all age-groups have the same length of follow-up.

Rates are adjusted for all the characteristics shown in figure S7.

Vertical lines are 95% confidence intervals.

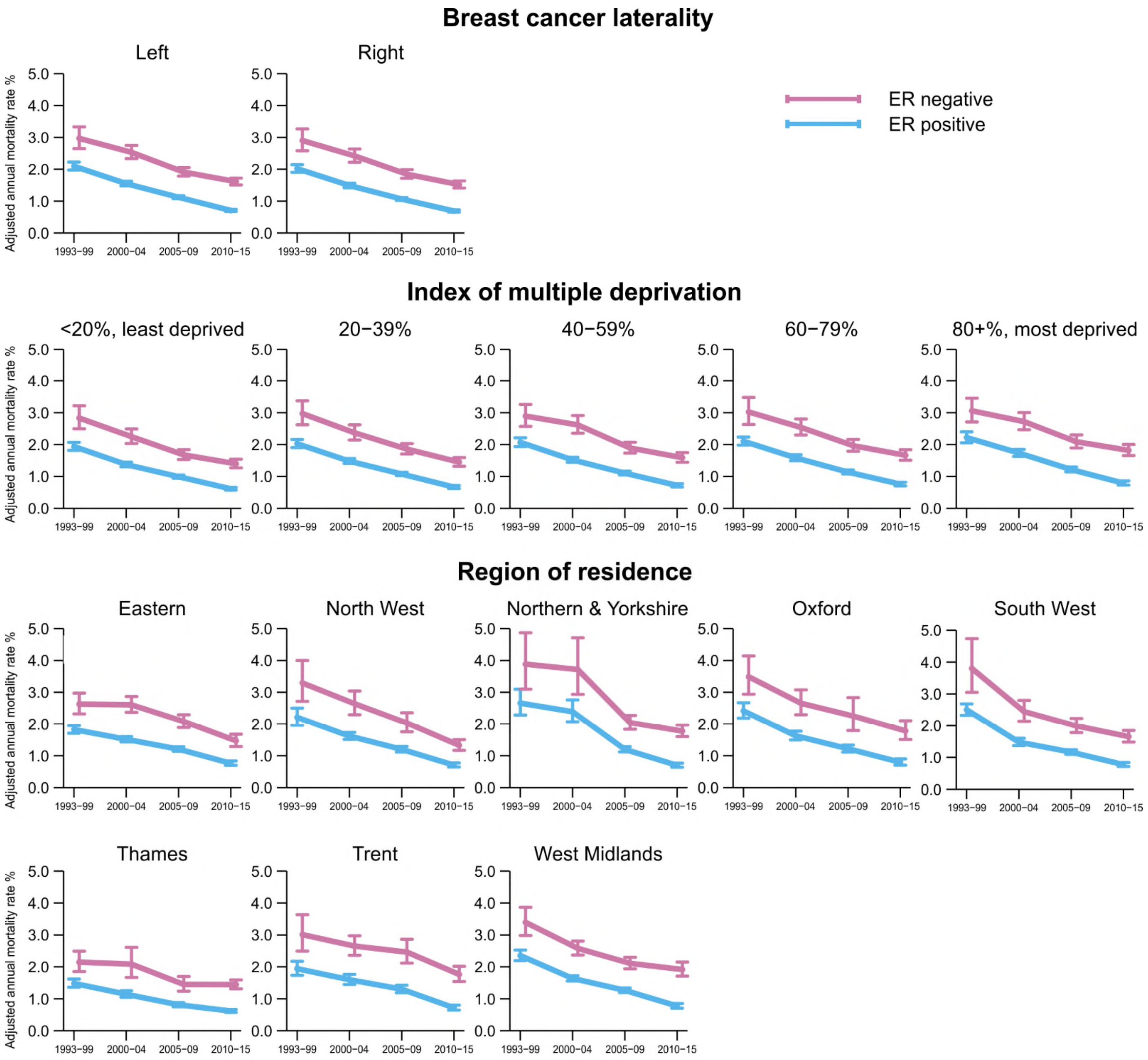

Figure S12: Adjusted annual breast cancer mortality rates in women with early breast cancer with ER-positive or ER-negative disease by calendar period of diagnosis, according to breast cancer laterality, index of multiple deprivation and region of residence

Further details are in figures S18-S20.

Vertical lines are 95% confidence intervals.

Age at diagnosis

ER-positive

Age and calendar period	Deaths/ Women	Adjusted annual mortality rate % (95% CI)	Rate ratio
**Age at diagnosis: 18- 39 years**			
1993- 1999	1,900/5,420	2.14 (1.96, 2.32)	1.00
2000- 2004	1,316/4,908	1.64 (1.51, 1.77)	0.77
2005- 2009	758/4,762	1.05 (0.96, 1.16)	0.49
2010- 2015	227/5,066	0.66 (0.57, 0.76)	0.31
**Age at diagnosis: 40- 49 years**			
1993- 1999	4,000/15,412	1.63 (1.52, 1.76)	1.00
2000- 2004	2,935/15,538	1.22 (1.16, 1.28)	0.75
2005- 2009	2,057/18,514	0.82 (0.77, 0.87)	0.50
2010- 2015	652/23,969	0.45 (0.41, 0.49)	0.28
**Age at diagnosis: 50- 64 years**			
1993- 1999	7,913/36,418	1.89 (1.78, 2.02)	1.00
2000- 2004	5,875/41,689	1.37 (1.31, 1.44)	0.72
2005- 2009	3,913/45,377	0.99 (0.95, 1.04)	0.52
2010- 2015	1,163/54,226	0.55 (0.52, 0.59)	0.29
**Age at diagnosis: 65- 70 years**			
1993- 1999	2,902/10,751	2.28 (2.13, 2.44)	1.00
2000- 2004	2,188/12,156	1.61 (1.51, 1.72)	0.71
2005- 2009	1,579/17,444	1.24 (1.17, 1.32)	0.54
2010- 2015	605/24,236	0.74 (0.68, 0.80)	0.32
**Age at diagnosis: 71- 79 years**			
1993- 1999	3,627/13,900	2.49 (2.33, 2.66)	1.00
2000- 2004	2,910/13,991	1.97 (1.85, 2.09)	0.79
2005- 2009	2,177/14,131	1.49 (1.41, 1.57)	0.60
2010- 2015	1,002/18,725	1.06 (0.99, 1.14)	0.43
**Age at diagnosis: 80- 89 years**			
1993- 1999	1,317/5,062	3.78 (3.50, 4.09)	1.00
2000- 2004	1,117/5,496	2.66 (2.46, 2.88)	0.70
2005- 2009	1,021/6,253	1.96 (1.81, 2.12)	0.52
2010- 2015	746/8,172	1.78 (1.65, 1.93)	0.47

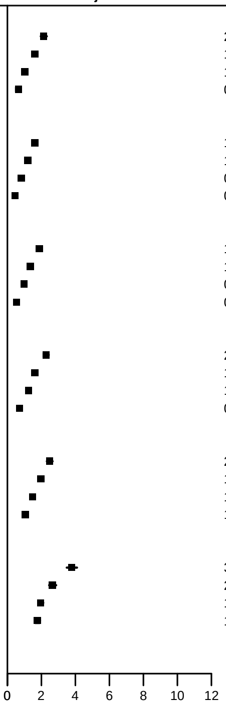

ER-negative

Age and calendar period	Deaths/ Women	Adjusted annual mortality rate % (95% CI)	Rate ratio
**Age at diagnosis: 18- 39 years**			
1993- 1999	1,042/2,431	2.87 (2.51, 3.28)	1.00
2000- 2004	727/2,093	2.28 (2.03, 2.55)	0.79
2005- 2009	443/1,789	1.52 (1.34, 1.72)	0.53
2010- 2015	197/1,650	1.22 (1.05, 1.43)	0.43
**Age at diagnosis: 40- 49 years**			
1993- 1999	2,281/6,305	2.38 (2.15, 2.63)	1.00
2000- 2004	1,127/3,774	1.95 (1.77, 2.15)	0.82
2005- 2009	858/3,854	1.41 (1.27, 1.56)	0.59
2010- 2015	423/3,938	1.10 (0.99, 1.23)	0.46
**Age at diagnosis: 50- 64 years**			
1993- 1999	3,673/11,047	2.78 (2.48, 3.12)	1.00
2000- 2004	2,514/9,071	2.22 (2.04, 2.42)	0.80
2005- 2009	1,732/8,363	1.64 (1.52, 1.77)	0.59
2010- 2015	739/7,847	1.21 (1.10, 1.32)	0.43
**Age at diagnosis: 65- 70 years**			
1993- 1999	984/2,633	3.14 (2.68, 3.67)	1.00
2000- 2004	725/2,242	2.69 (2.37, 3.05)	0.86
2005- 2009	608/2,762	2.05 (1.82, 2.32)	0.66
2010- 2015	363/3,182	1.61 (1.44, 1.81)	0.51
**Age at diagnosis: 71- 79 years**			
1993- 1999	1,072/3,087	3.17 (2.67, 3.77)	1.00
2000- 2004	1,020/2,711	3.37 (3.02, 3.76)	1.06
2005- 2009	984/2,990	2.74 (2.48, 3.01)	0.86
2010- 2015	660/3,246	2.31 (2.11, 2.53)	0.73
**Age at diagnosis: 80- 89 years**			
1993- 1999	277/883	3.86 (3.09, 4.82)	1.00
2000- 2004	414/1,151	4.02 (3.46, 4.66)	1.04
2005- 2009	593/1,684	3.65 (3.26, 4.07)	0.94
2010- 2015	595/2,075	3.73 (3.38, 4.11)	0.97

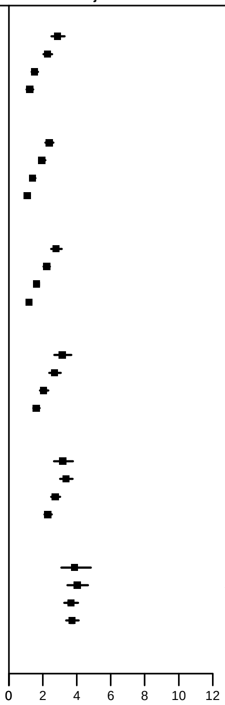

Figure S13: Adjusted annual breast cancer mortality rates in women with early breast cancer with ER-positive or ER-negative disease by calendar period of diagnosis, according to age at diagnosis (Figure 3)

For each characteristic, rates are adjusted for all the characteristics shown in figure S7.

Cancer screen-detected

ER-positive

Screening and calendar period	Deaths/ Women	Adjusted annual mortality rate % (95% CI)	Rate ratio
**Eligible: screen- detected**			
1993- 1999	1,741/12,138	1.69 (1.56, 1.84)	1.00
2000- 2004	1,698/18,063	1.19 (1.11, 1.28)	0.70
2005- 2009	1,948/37,203	0.74 (0.70, 0.78)	0.43
2010- 2015	535/47,494	0.36 (0.33, 0.39)	0.21
**Eligible: not screen- detected**			
1993- 1999	6,171/24,279	2.24 (2.06, 2.42)	1.00
2000- 2004	4,176/23,626	1.62 (1.51, 1.73)	0.72
2005- 2009	3,544/25,618	1.26 (1.19, 1.33)	0.56
2010- 2015	1,234/30,968	0.76 (0.71, 0.82)	0.34
**Not eligible for screening**			
1993- 1999	13,748/50,547	2.11 (1.99, 2.24)	1.00
2000- 2004	10,468/52,091	1.57 (1.49, 1.66)	0.75
2005- 2009	6,015/43,662	1.12 (1.07, 1.18)	0.53
2010- 2015	2,628/55,934	0.78 (0.74, 0.82)	0.37

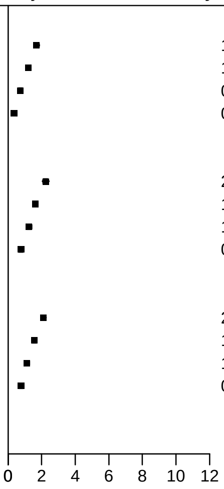

ER-negative

Screening and calendar period	Deaths/ Women	Adjusted annual mortality rate % (95% CI)	Rate ratio
**Eligible: screen- detected**			
1993- 1999	630/2,842	2.53 (2.18, 2.93)	1.00
2000- 2004	402/2,126	2.16 (1.82, 2.58)	0.86
2005- 2009	511/4,068	1.28 (1.12, 1.46)	0.51
2010- 2015	217/4,302	0.84 (0.72, 0.97)	0.33
**Eligible: not screen- detected**			
1993- 1999	3,043/8,204	3.34 (2.94, 3.80)	1.00
2000- 2004	2,112/6,944	2.64 (2.35, 2.96)	0.79
2005- 2009	1,829/7,057	2.02 (1.84, 2.22)	0.61
2010- 2015	884/6,727	1.58 (1.44, 1.73)	0.47
**Not eligible for screening**			
1993- 1999	5,658/15,342	2.93 (2.57, 3.34)	1.00
2000- 2004	4,015/11,973	2.56 (2.35, 2.79)	0.87
2005- 2009	2,879/10,318	1.98 (1.82, 2.15)	0.68
2010- 2015	1,877/10,910	1.75 (1.62, 1.90)	0.60

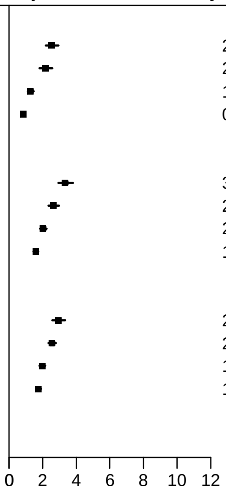

Figure S14: Adjusted annual breast cancer mortality rates in women with early breast cancer with ER-positive or ER-negative disease by calendar period of diagnosis, according to screening status (Figure 4)

For each characteristic, rates are adjusted for all the characteristics shown in figure S7.

Tumour size

ER-positive

Size and calendar period	Deaths/ Women	Adjusted annual mortality rate % (95% CI)	Rate ratio
**Tumour size: 1- 20 mm**			
1993- 1999	9,726/54,238	1.55 (1.47, 1.63)	1.00
2000- 2004	5,962/56,958	1.05 (1.00, 1.10)	0.68
2005- 2009	3,646/63,833	0.75 (0.71, 0.78)	0.48
2010- 2015	1,167/82,658	0.41 (0.38, 0.44)	0.27
**Tumour size: 21- 50 mm**			
1993- 1999	10,643/29,954	2.46 (2.30, 2.63)	1.00
2000- 2004	8,905/33,354	1.89 (1.80, 1.99)	0.77
2005- 2009	6,631/38,194	1.38 (1.33, 1.44)	0.56
2010- 2015	2,585/46,181	0.91 (0.87, 0.96)	0.37
**Tumour size: >50 mm**			
1993- 1999	1,292/2,772	3.04 (2.72, 3.38)	1.00
2000- 2004	1,476/3,468	2.72 (2.52, 2.93)	0.89
2005- 2009	1,230/4,456	1.80 (1.67, 1.94)	0.59
2010- 2015	645/5,557	1.37 (1.25, 1.49)	0.45

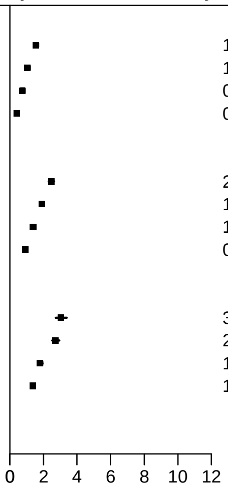

ER-negative

Size and calendar period	Deaths/ Women	Adjusted annual mortality rate % (95% CI)	Rate ratio
**Tumour size: 1- 20 mm**			
1993- 1999	3,075/12,494	2.25 (1.97, 2.58)	1.00
2000- 2004	1,965/9,852	1.81 (1.65, 1.99)	0.81
2005- 2009	1,366/9,824	1.31 (1.20, 1.43)	0.58
2010- 2015	732/10,677	0.98 (0.89, 1.07)	0.43
**Tumour size: 21- 50 mm**			
1993- 1999	5,440/12,420	3.49 (3.12, 3.91)	1.00
2000- 2004	3,855/9,978	2.94 (2.71, 3.19)	0.84
2005- 2009	3,119/10,119	2.27 (2.12, 2.44)	0.65
2010- 2015	1,801/10,056	1.90 (1.78, 2.03)	0.54
**Tumour size: >50 mm**			
1993- 1999	816/1,473	4.30 (3.80, 4.87)	1.00
2000- 2004	709/1,214	4.22 (3.71, 4.80)	0.98
2005- 2009	735/1,499	3.24 (2.90, 3.63)	0.75
2010- 2015	445/1,206	3.29 (2.90, 3.72)	0.76

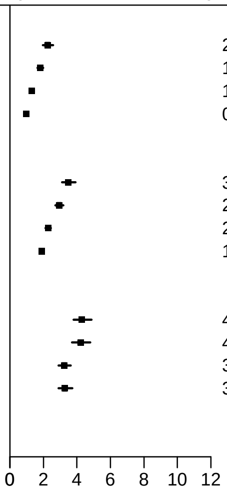

Figure S15: Adjusted annual breast cancer mortality rates in women with early breast cancer with ER-positive or ER-negative disease by calendar period of diagnosis, according to tumour size (Figure 5)

For each characteristic, rates are adjusted for all the characteristics shown in figure S7.

Number of positive nodes

ER-positive

Nodes and calendar period	Deaths/ Women	Adjusted annual mortality rate % (95% CI)	Rate ratio
**Number nodes positive: 0**			
1993- 1999	6,201/43,652	1.11 (1.05, 1.19)	1.00
2000- 2004	4,792/51,405	0.81 (0.76, 0.87)	0.73
2005- 2009	3,091/58,786	0.59 (0.56, 0.62)	0.53
2010- 2015	1,404/88,217	0.36 (0.34, 0.38)	0.32
**Number nodes positive: 1 to 3**			
1993- 1999	9,162/30,443	2.49 (2.32, 2.67)	1.00
2000- 2004	6,048/28,548	1.82 (1.73, 1.92)	0.73
2005- 2009	4,291/33,228	1.27 (1.22, 1.33)	0.51
2010- 2015	1,493/34,857	0.76 (0.72, 0.81)	0.31
**Number nodes positive: 4 to 9**			
1993- 1999	4,521/9,684	4.33 (4.05, 4.63)	1.00
2000- 2004	3,447/9,720	3.14 (2.97, 3.32)	0.73
2005- 2009	2,472/10,016	2.30 (2.17, 2.43)	0.53
2010- 2015	842/7,918	1.59 (1.46, 1.73)	0.37
**Number nodes positive: 10 or more**			
1993- 1999	1,776/3,184	5.80 (5.24, 6.41)	1.00
2000- 2004	2,055/4,106	4.89 (4.56, 5.25)	0.84
2005- 2009	1,651/4,453	3.61 (3.36, 3.87)	0.62
2010- 2015	657/3,404	2.81 (2.57, 3.08)	0.49

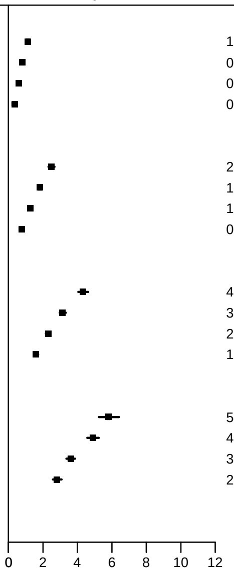

ER-negative

Nodes and calendar period	Deaths/ Women	Adjusted annual mortality rate % (95% CI)	Rate ratio
**Number nodes positive: 0**			
1993- 1999	1,966/10,120	1.56 (1.35, 1.81)	1.00
2000- 2004	2,178/11,689	1.32 (1.19, 1.47)	0.85
2005- 2009	1,482/10,906	1.06 (0.98, 1.15)	0.68
2010- 2015	1,088/13,681	0.88 (0.81, 0.94)	0.56
**Number nodes positive: 1 to 3**			
1993- 1999	4,093/10,725	3.54 (3.12, 4.01)	1.00
2000- 2004	2,283/5,909	3.04 (2.80, 3.31)	0.86
2005- 2009	1,713/6,315	2.21 (2.03, 2.41)	0.63
2010- 2015	959/5,636	1.78 (1.63, 1.94)	0.50
**Number nodes positive: 4 to 9**			
1993- 1999	2,126/3,813	6.08 (5.36, 6.91)	1.00
2000- 2004	1,285/2,313	4.90 (4.41, 5.44)	0.81
2005- 2009	1,115/2,575	3.79 (3.43, 4.20)	0.62
2010- 2015	509/1,683	3.10 (2.76, 3.47)	0.51
**Number nodes positive: 10 or more**			
1993- 1999	1,144/1,729	8.20 (7.21, 9.32)	1.00
2000- 2004	782/1,131	7.21 (6.36, 8.17)	0.88
2005- 2009	910/1,647	5.32 (4.79, 5.91)	0.65
2010- 2015	421/939	4.90 (4.34, 5.54)	0.60

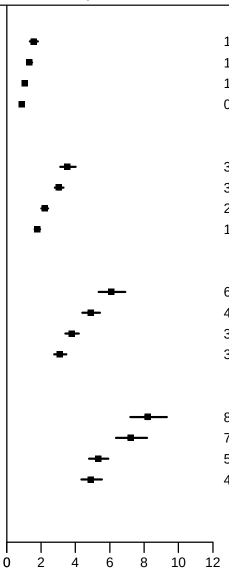

Figure S16: Adjusted annual breast cancer mortality rates in women with early breast cancer with ER-positive or ER-negative disease by calendar period of diagnosis, according to number of positive nodes (Figure 6)

For each characteristic, rates are adjusted for all the characteristics shown in figure S7.

Tumour grade

ER-positive

Grade and calendar period	Deaths/ Women	Adjusted annual mortality rate % (95% CI)	Rate ratio
**Tumour grade: Low**			
1993- 1999	2,943/23,052	1.13 (1.07, 1.19)	1.00
2000- 2004	1,558/22,709	0.72 (0.68, 0.76)	0.64
2005- 2009	661/21,689	0.44 (0.41, 0.48)	0.39
2010- 2015	150/26,347	0.19 (0.16, 0.22)	0.17
**Tumour grade: Medium**			
1993- 1999	11,013/42,103	1.91 (1.82, 2.01)	1.00
2000- 2004	8,542/48,629	1.40 (1.35, 1.45)	0.73
2005- 2009	5,716/57,560	0.96 (0.93, 0.99)	0.50
2010- 2015	1,880/75,509	0.51 (0.48, 0.54)	0.27
**Tumour grade: High**			
1993- 1999	7,705/21,809	2.56 (2.34, 2.81)	1.00
2000- 2004	6,243/22,442	2.01 (1.87, 2.16)	0.79
2005- 2009	5,129/27,234	1.56 (1.47, 1.65)	0.61
2010- 2015	2,367/32,540	1.23 (1.17, 1.29)	0.48

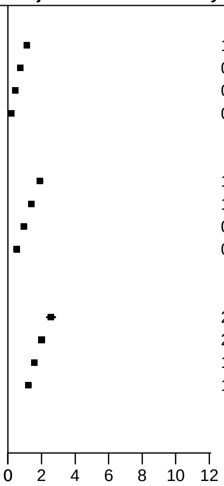

ER-negative

Grade and calendar period	Deaths/ Women	Adjusted annual mortality rate % (95% CI)	Rate ratio
**Tumour grade: Low**			
1993- 1999	276/2,204	1.37 (1.08, 1.75)	1.00
2000- 2004	64/717	1.15 (0.80, 1.66)	0.84
2005- 2009	14/346	0.59 (0.30, 1.17)	0.43
2010- 2015	15/390	0.82 (0.49, 1.39)	0.60
**Tumour grade: Medium**			
1993- 1999	2,451/8,257	2.74 (2.39, 3.15)	1.00
2000- 2004	1,086/4,732	2.35 (2.08, 2.64)	0.86
2005- 2009	621/4,058	1.59 (1.42, 1.77)	0.58
2010- 2015	374/4,332	1.32 (1.17, 1.48)	0.48
**Tumour grade: High**			
1993- 1999	6,603/15,926	3.87 (3.49, 4.29)	1.00
2000- 2004	5,379/15,594	3.27 (3.05, 3.51)	0.84
2005- 2009	4,585/17,039	2.53 (2.39, 2.68)	0.65
2010- 2015	2,589/17,217	2.11 (2.00, 2.22)	0.54

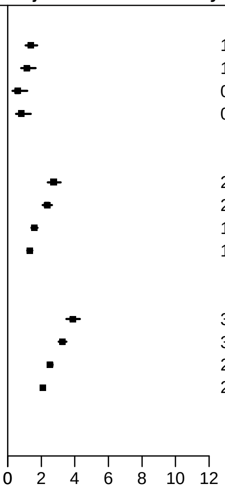

Figure S17: Adjusted annual breast cancer mortality rates in women with early breast cancer with ER-positive or ER-negative disease by calendar period of diagnosis, according to tumour grade (Figure 7)

For each characteristic, rates are adjusted for all the characteristics shown in figure S7.

Breast cancer laterality

ER-positive

Laterality and calendar period	Deaths/ Women	Adjusted annual mortality rate % (95% CI)	Rate ratio
**Left**			
1993- 1999	11,138/44,240	2.10 (1.97, 2.23)	1.00
2000- 2004	8,498/48,184	1.55 (1.48, 1.62)	0.74
2005- 2009	6,059/54,802	1.12 (1.08, 1.17)	0.53
2010- 2015	2,286/68,762	0.71 (0.67, 0.74)	0.34
**Right**			
1993- 1999	10,523/42,724	2.02 (1.90, 2.14)	1.00
2000- 2004	7,845/45,596	1.49 (1.42, 1.56)	0.74
2005- 2009	5,447/51,682	1.07 (1.02, 1.11)	0.53
2010- 2015	2,112/65,634	0.69 (0.66, 0.73)	0.34

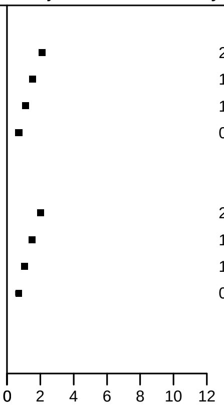

ER-negative

Laterality and calendar period	Deaths/ Women	Adjusted annual mortality rate % (95% CI)	Rate ratio
**Left**			
1993- 1999	5,131/14,467	2.97 (2.65, 3.33)	1.00
2000- 2004	3,428/10,908	2.53 (2.33, 2.75)	0.85
2005- 2009	2,712/11,027	1.91 (1.79, 2.05)	0.64
2010- 2015	1,604/11,513	1.61 (1.51, 1.72)	0.54
**Right**			
1993- 1999	4,200/11,921	2.90 (2.58, 3.27)	1.00
2000- 2004	3,101/10,136	2.42 (2.22, 2.64)	0.83
2005- 2009	2,509/10,416	1.85 (1.72, 1.99)	0.64
2010- 2015	1,375/10,427	1.52 (1.41, 1.63)	0.52

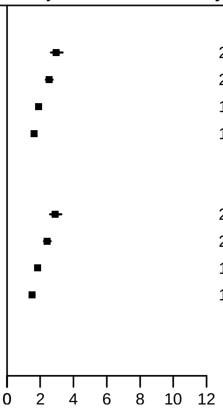

Figure S18: Adjusted annual breast cancer mortality rates in women with early breast cancer with ER-positive or ER-negative disease by calendar period of diagnosis, according to breast cancer laterality (Figure S12)

For each characteristic, rates are adjusted for all the characteristics shown in figure S7.

Index of multiple deprivation

ER-positive

Multiple deprivation index and calendar period	Deaths/ Women	Adjusted annual mortality rate % (95% CI)	Rate ratio
**<20%, least deprived**			
1993- 1999	4,587/19,649	1.94 (1.82, 2.07)	1.00
2000- 2004	3,453/21,573	1.37 (1.30, 1.45)	0.71
2005- 2009	2,438/25,011	0.99 (0.94, 1.05)	0.51
2010- 2015	925/32,134	0.61 (0.57, 0.66)	0.32
**20- 39%**			
1993- 1999	4,879/19,962	2.03 (1.91, 2.16)	1.00
2000- 2004	3,638/21,565	1.48 (1.41, 1.56)	0.73
2005- 2009	2,578/24,629	1.08 (1.02, 1.13)	0.53
2010- 2015	965/31,345	0.67 (0.62, 0.71)	0.33
**40- 59%**			
1993- 1999	4,492/18,099	2.07 (1.94, 2.21)	1.00
2000- 2004	3,482/19,955	1.52 (1.44, 1.60)	0.73
2005- 2009	2,479/22,672	1.10 (1.05, 1.16)	0.53
2010- 2015	945/28,369	0.72 (0.67, 0.77)	0.35
**60- 79%**			
1993- 1999	4,292/16,524	2.11 (1.98, 2.24)	1.00
2000- 2004	3,120/17,029	1.58 (1.49, 1.68)	0.75
2005- 2009	2,166/19,062	1.14 (1.08, 1.20)	0.54
2010- 2015	850/23,924	0.76 (0.70, 0.82)	0.36
**80+%, most deprived**			
1993- 1999	3,411/12,727	2.22 (2.06, 2.40)	1.00
2000- 2004	2,649/13,657	1.73 (1.63, 1.85)	0.78
2005- 2009	1,844/15,108	1.22 (1.14, 1.29)	0.55
2010- 2015	710/18,623	0.79 (0.73, 0.86)	0.36

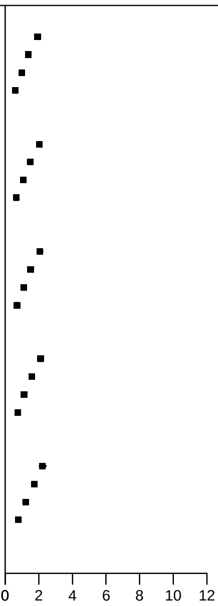

ER-negative

Multiple deprivation index and calendar period	Deaths/ Women	Adjusted annual mortality rate % (95% CI)	Rate ratio
**<20%, least deprived**			
1993- 1999	1,944/5,736	2.83 (2.50, 3.22)	1.00
2000- 2004	1,271/4,460	2.25 (2.03, 2.49)	0.79
2005- 2009	999/4,589	1.68 (1.53, 1.84)	0.59
2010- 2015	596/4,892	1.40 (1.27, 1.54)	0.49
**20- 39%**			
1993- 1999	1,904/5,426	2.97 (2.62, 3.37)	1.00
2000- 2004	1,384/4,639	2.37 (2.14, 2.62)	0.80
2005- 2009	1,137/4,730	1.86 (1.70, 2.03)	0.62
2010- 2015	593/4,766	1.45 (1.32, 1.59)	0.49
**40- 59%**			
1993- 1999	2,014/5,754	2.89 (2.57, 3.26)	1.00
2000- 2004	1,352/4,187	2.62 (2.36, 2.91)	0.91
2005- 2009	1,049/4,314	1.90 (1.73, 2.07)	0.65
2010- 2015	609/4,455	1.59 (1.45, 1.75)	0.55
**60- 79%**			
1993- 1999	1,685/4,623	3.02 (2.63, 3.48)	1.00
2000- 2004	1,316/4,101	2.54 (2.30, 2.80)	0.84
2005- 2009	1,071/4,210	1.97 (1.79, 2.16)	0.65
2010- 2015	583/4,060	1.66 (1.51, 1.84)	0.55
**80+%, most deprived**			
1993- 1999	1,781/4,849	3.06 (2.71, 3.46)	1.00
2000- 2004	1,204/3,655	2.72 (2.47, 3.00)	0.89
2005- 2009	963/3,599	2.09 (1.89, 2.30)	0.68
2010- 2015	597/3,765	1.82 (1.65, 2.00)	0.59

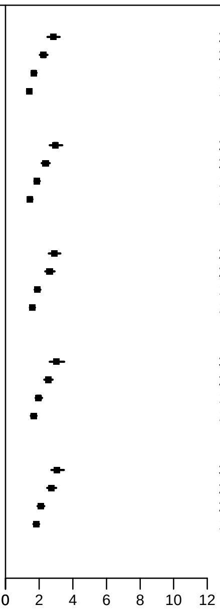

Figure S19: Adjusted annual breast cancer mortality rates in women with early breast cancer with ER-positive or ER-negative disease by calendar period of diagnosis, according to index of multiple deprivation (Figure S12)

For each characteristic, rates are adjusted for all the characteristics shown in figure S7.

Region of residence

ER-positive

Region of residence and calendar period	Deaths/ Women	Adjusted annual mortality rate % (95% CI)	Rate ratio
**Eastern**			
1993- 1999	2,281/9,481	1.82 (1.71, 1.95)	1.00
2000- 2004	1,772/10,527	1.52 (1.44, 1.60)	0.83
2005- 2009	1,174/11,474	1.21 (1.14, 1.28)	0.66
2010- 2015	533/15,994	0.76 (0.70, 0.83)	0.42
**North West**			
1993- 1999	3,396/13,108	2.21 (1.96, 2.50)	1.00
2000- 2004	2,188/12,776	1.62 (1.51, 1.73)	0.73
2005- 2009	1,562/14,491	1.20 (1.12, 1.29)	0.54
2010- 2015	543/17,469	0.71 (0.65, 0.77)	0.32
**Northern & Yorkshire**			
1993- 1999	2,462/9,824	2.66 (2.28, 3.10)	1.00
2000- 2004	2,044/12,182	2.39 (2.07, 2.76)	0.90
2005- 2009	1,361/13,696	1.20 (1.12, 1.28)	0.45
2010- 2015	522/17,104	0.70 (0.64, 0.77)	0.26
**Oxford**			
1993- 1999	1,221/5,425	2.42 (2.19, 2.67)	1.00
2000- 2004	843/5,442	1.63 (1.49, 1.77)	0.67
2005- 2009	668/6,481	1.22 (1.11, 1.34)	0.50
2010- 2015	277/8,143	0.80 (0.70, 0.90)	0.33
**South West**			
1993- 1999	4,252/16,764	2.50 (2.33, 2.69)	1.00
2000- 2004	2,181/13,613	1.48 (1.37, 1.60)	0.59
2005- 2009	1,693/16,541	1.17 (1.10, 1.24)	0.47
2010- 2015	723/21,333	0.77 (0.72, 0.84)	0.31
**Thames**			
1993- 1999	4,214/17,244	1.48 (1.36, 1.62)	1.00
2000- 2004	4,083/20,839	1.14 (1.05, 1.25)	0.77
2005- 2009	2,735/22,569	0.81 (0.75, 0.88)	0.55
2010- 2015	948/27,808	0.62 (0.57, 0.67)	0.42
**Trent**			
1993- 1999	1,047/4,103	1.94 (1.74, 2.18)	1.00
2000- 2004	1,416/8,054	1.60 (1.45, 1.76)	0.82
2005- 2009	1,005/9,703	1.30 (1.19, 1.42)	0.67
2010- 2015	377/12,830	0.72 (0.64, 0.80)	0.37
**West Midlands**			
1993- 1999	2,785/11,013	2.35 (2.19, 2.53)	1.00
2000- 2004	1,813/10,345	1.64 (1.56, 1.72)	0.70
2005- 2009	1,307/11,525	1.27 (1.20, 1.34)	0.54
2010- 2015	472/13,713	0.77 (0.70, 0.85)	0.33

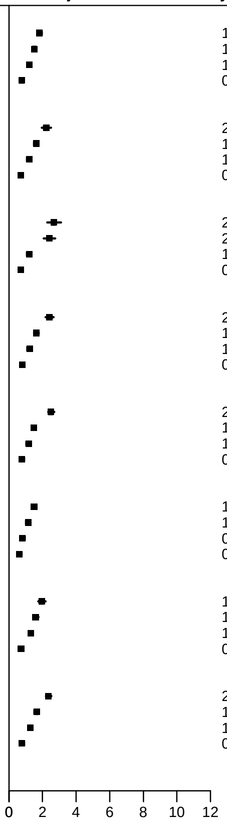

ER-negative

Region of residence and calendar period	Deaths/ Women	Adjusted annual mortality rate % (95% CI)	Rate ratio
**Eastern**			
1993- 1999	953/2,713	2.63 (2.32, 2.97)	1.00
2000- 2004	704/2,206	2.61 (2.37, 2.87)	0.99
2005- 2009	494/2,148	2.08 (1.89, 2.29)	0.79
2010- 2015	261/2,243	1.47 (1.29, 1.67)	0.56
**North West**			
1993- 1999	1,172/3,161	3.29 (2.71, 4.00)	1.00
2000- 2004	982/3,159	2.64 (2.29, 3.04)	0.80
2005- 2009	709/3,022	2.03 (1.75, 2.36)	0.62
2010- 2015	320/2,810	1.33 (1.17, 1.50)	0.40
**Northern & Yorkshire**			
1993- 1999	1,531/4,270	3.88 (3.10, 4.87)	1.00
2000- 2004	948/3,338	3.72 (2.93, 4.71)	0.96
2005- 2009	767/3,342	2.04 (1.84, 2.27)	0.53
2010- 2015	454/3,170	1.77 (1.60, 1.97)	0.46
**Oxford**			
1993- 1999	762/2,350	3.49 (2.94, 4.14)	1.00
2000- 2004	352/1,200	2.66 (2.29, 3.08)	0.76
2005- 2009	158/667	2.25 (1.79, 2.83)	0.65
2010- 2015	167/1,230	1.78 (1.51, 2.10)	0.51
**South West**			
1993- 1999	705/1,947	3.80 (3.05, 4.74)	1.00
2000- 2004	1,174/3,975	2.45 (2.14, 2.80)	0.64
2005- 2009	846/3,680	1.99 (1.78, 2.23)	0.52
2010- 2015	429/3,186	1.65 (1.48, 1.85)	0.44
**Thames**			
1993- 1999	2,544/7,478	2.15 (1.85, 2.49)	1.00
2000- 2004	805/2,253	2.09 (1.67, 2.61)	0.97
2005- 2009	1,264/4,661	1.45 (1.24, 1.70)	0.68
2010- 2015	688/4,755	1.44 (1.31, 1.59)	0.67
**Trent**			
1993- 1999	404/1,070	3.01 (2.49, 3.63)	1.00
2000- 2004	774/2,400	2.65 (2.36, 2.97)	0.88
2005- 2009	388/1,514	2.46 (2.12, 2.86)	0.82
2010- 2015	301/2,278	1.76 (1.54, 2.02)	0.59
**West Midlands**			
1993- 1999	1,257/3,395	3.40 (2.98, 3.87)	1.00
2000- 2004	787/2,509	2.58 (2.37, 2.81)	0.76
2005- 2009	590/2,407	2.11 (1.94, 2.30)	0.62
2010- 2015	355/2,264	1.92 (1.71, 2.15)	0.56

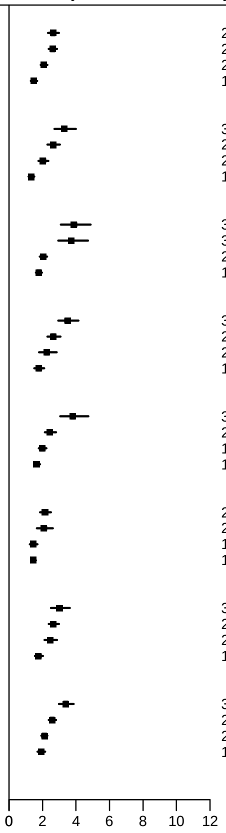

Figure S20: Adjusted annual breast cancer mortality rates in women with early breast cancer with ER-positive or ER-negative disease by calendar period of diagnosis, according to region of residence (Figure S12)

For each characteristic, rates are adjusted for all the characteristics shown in figure S7.

Table S7: Tests for interactions in adjusted annual breast cancer mortality rates between pairs of characteristics in women with early breast cancer with ER-positive or ER-negative disease

Factors	P value	
	ER-positive	ER-negative
Time since diagnosis x calendar period of diagnosis	$1.2x10^{-20}$	$5.4x10^{-03}$
Time since diagnosis x age at diagnosis	$4.1x10^{-48}$	$8.0x10^{-14}$
Time since diagnosis x cancer screen-detected	$1.2x10^{-54}$	$2.6x10^{-11}$
Time since diagnosis x tumour size	$1.7x10^{-50}$	$5.7x10^{-24}$
Time since diagnosis x number of positive nodes	$2.8x10^{-44}$	$1.6x10^{-02}$
Time since diagnosis x tumour grade	$0.0x10^{+00}$	$1.5x10^{-64}$
Time since diagnosis x breast cancer laterality	$6.4x10^{-01}$	$9.4x10^{-01}$
Time since diagnosis x index of multiple deprivation	$3.2x10^{-03}$	$9.1x10^{-01}$
Time since diagnosis x region of residence	$4.9x10^{-03}$	$1.0x10^{+00}$
Calendar period of diagnosis x age at diagnosis	$3.1x10^{-23}$	$7.6x10^{-18}$
Calendar period of diagnosis x cancer screen-detected	$7.1x10^{-17}$	$3.2x10^{-04}$
Calendar period of diagnosis x tumour size	$8.3x10^{-17}$	$2.1x10^{-06}$
Calendar period of diagnosis x number of positive nodes	$6.5x10^{-07}$	$5.5x10^{-01}$
Calendar period of diagnosis x tumour grade	$2.3x10^{-42}$	$2.4x10^{-01}$
Calendar period of diagnosis x breast cancer laterality	$9.3x10^{-01}$	$8.8x10^{-01}$
Calendar period of diagnosis x index of multiple deprivation	$4.2x10^{-01}$	$3.2x10^{-01}$
Calendar period of diagnosis x region of residence	$7.8x10^{-18}$	$3.9x10^{-05}$
Age at diagnosis x cancer screen-detected	$7.3x10^{-06}$	$2.3x10^{-01}$
Age at diagnosis x tumour size	$8.5x10^{-07}$	$8.1x10^{-01}$
Age at diagnosis x number of positive nodes	$4.3x10^{-01}$	$9.7x10^{-01}$
Age at diagnosis x tumour grade	$3.2x10^{-36}$	$1.4x10^{-04}$
Age at diagnosis x breast cancer laterality	$8.1x10^{-01}$	$8.3x10^{-01}$
Age at diagnosis x index of multiple deprivation	$8.0x10^{-01}$	$9.9x10^{-01}$
Age at diagnosis x region of residence	$2.2x10^{-07}$	$9.1x10^{-01}$
Cancer screen-detected x tumour size	$2.8x10^{-02}$	$2.2x10^{-01}$
Cancer screen-detected x number of positive nodes	$2.0x10^{-02}$	$6.4x10^{-01}$
Cancer screen-detected x tumour grade	$2.2x10^{-08}$	$3.5x10^{-01}$
Cancer screen-detected x breast cancer laterality	$2.8x10^{-01}$	$3.9x10^{-01}$
Cancer screen-detected x index of multiple deprivation	$9.1x10^{-01}$	$9.0x10^{-01}$
Cancer screen-detected x region of residence	$8.9x10^{-02}$	$5.2x10^{-01}$
Tumour size x number of positive nodes	$1.4x10^{-05}$	$1.4x10^{-03}$
Tumour size x tumour grade	$1.1x10^{-08}$	$2.3x10^{-01}$
Tumour size x breast cancer laterality	$6.8x10^{-01}$	$8.2x10^{-01}$
Tumour size x index of multiple deprivation	$6.4x10^{-01}$	$7.9x10^{-01}$
Tumour size x region of residence	$9.2x10^{-01}$	$8.1x10^{-01}$
Number of positive nodes x tumour grade	$5.4x10^{-03}$	$9.3x10^{-01}$
Number of positive nodes x breast cancer laterality	$8.2x10^{-01}$	$7.9x10^{-01}$
Number of positive nodes x index of multiple deprivation	$9.8x10^{-01}$	$9.9x10^{-01}$
Number of positive nodes x region of residence	$8.2x10^{-05}$	$3.9x10^{-01}$
Tumour grade x breast cancer laterality	$9.9x10^{-01}$	$9.4x10^{-01}$
Tumour grade x index of multiple deprivation	$1.9x10^{-01}$	$9.1x10^{-01}$
Tumour grade x region of residence	$7.4x10^{-02}$	$5.7x10^{-01}$
Breast cancer laterality x index of multiple deprivation	$5.5x10^{-01}$	$8.6x10^{-01}$
Breast cancer laterality x region of residence	$6.7x10^{-01}$	$8.0x10^{-01}$
Index of multiple deprivation x region of residence	$9.8x10^{-01}$	$1.0x10^{+00}$

All factors treated as categorical.

ER-positive

Characteristic	Deaths/ Women	Adjusted annual mortality rate % (95% CI)	Rate ratio
**Age at diagnosis (years) ($p=1.x10^{-123}$)**			
18- 39	227/5,066	0.62 (0.54, 0.72)	1.00
40- 49	652/23,969	0.46 (0.43, 0.50)	0.74
50- 64	1,163/54,226	0.63 (0.59, 0.67)	1.01
65- 70	605/24,236	0.87 (0.80, 0.95)	1.39
71- 79	1,002/18,725	1.12 (1.05, 1.20)	1.80
80- 89	746/8,172	1.84 (1.70, 1.99)	2.95
**Cancer screen- detected ($p=3.5x10^{-31}$)**			
Screen detected	535/47,494	0.46 (0.42, 0.51)	1.00
Not screen- detected	3,863/86,902	0.86 (0.83, 0.89)	1.88
**Tumour size (mm) ($p=1.4x10^{-76}$)**			
1- 20	1,167/82,658	0.49 (0.46, 0.53)	1.00
21- 50	2,585/46,181	0.93 (0.89, 0.97)	1.88
>50	645/5,557	1.37 (1.26, 1.50)	2.78
**Number of positive nodes ($p=1.x10^{-235}$)**			
0	1,404/88,217	0.45 (0.42, 0.47)	1.00
1 to 3	1,493/34,857	0.87 (0.82, 0.92)	1.94
4 to 9	842/7,918	1.70 (1.57, 1.84)	3.79
10 or more	657/3,404	2.96 (2.70, 3.24)	6.60
**Tumour grade ($p=3.x10^{-167}$)**			
Low	150/26,347	0.21 (0.18, 0.25)	1.00
Medium	1,880/75,509	0.53 (0.51, 0.56)	2.54
High	2,367/32,540	1.30 (1.24, 1.36)	6.22
**HER2 status ($p=3.4x10^{-03}$)**			
Negative	3,761/119,913	0.81 (0.78, 0.84)	1.00
Positive	636/14,483	0.69 (0.63, 0.76)	0.86
**Breast cancer laterality ($p=4.4x10^{-01}$)**			
Left	2,286/68,762	0.80 (0.76, 0.83)	1.00
Right	2,112/65,634	0.78 (0.74, 0.81)	0.98
**Index of multiple deprivation ($p=6.5x10^{-09}$)**			
<20% (least deprived)	925/32,134	0.69 (0.64, 0.74)	1.00
20- 39%	965/31,345	0.75 (0.70, 0.80)	1.09
40- 59%	945/28,369	0.80 (0.75, 0.86)	1.16
60- 79%	850/23,924	0.85 (0.79, 0.91)	1.23
80+% (most deprived)	710/18,623	0.91 (0.84, 0.98)	1.31
**Region of residence ($p=4.4x10^{-05}$)**			
Eastern	533/15,994	0.83 (0.76, 0.90)	1.00
North West	543/17,469	0.79 (0.72, 0.86)	0.95
Northern & Yorkshire	522/17,104	0.78 (0.72, 0.85)	0.95
Oxford	277/8,143	0.90 (0.80, 1.02)	1.09
South West	723/21,333	0.85 (0.79, 0.92)	1.03
Thames	948/27,808	0.68 (0.63, 0.73)	0.82
Trent	377/12,830	0.80 (0.72, 0.89)	0.97
West Midlands	472/13,713	0.84 (0.77, 0.93)	1.02
**Time since diagnosis (years) ($p=2.1x10^{-77}$)**			
<=1	279/134,397	0.29 (0.26, 0.33)	1.00
1- 2	817/133,653	0.66 (0.61, 0.71)	2.26
2- 3	1,089/132,109	0.92 (0.87, 0.98)	3.15
3- 4	1,165/130,048	1.04 (0.98, 1.11)	3.54
4- 5	1,046/127,489	0.98 (0.92, 1.05)	3.35
**All ER- positive**	4,398/134,397	0.79 (0.76, 0.81)	

ER-negative

Characteristic	Deaths/ Women	Adjusted annual mortality rate % (95% CI)	Rate ratio
**Age at diagnosis (years) ($p=4.5x10^{-67}$)**			
18- 39	197/1,650	1.70 (1.45, 2.00)	1.00
40- 49	423/3,938	1.54 (1.37, 1.74)	0.91
50- 64	739/7,847	1.81 (1.65, 1.99)	1.06
65- 70	363/3,182	2.43 (2.15, 2.74)	1.43
71- 79	660/3,246	3.23 (2.94, 3.56)	1.90
80- 89	595/2,075	4.96 (4.49, 5.48)	2.91
**Cancer screen- detected ($p=5.5x10^{-13}$)**			
Screen detected	217/4,302	1.40 (1.19, 1.63)	1.00
Not screen- detected	2,761/17,638	2.53 (2.36, 2.70)	1.81
**Tumour size (mm) ($p=5.6x10^{-50}$)**			
1- 20	732/10,677	1.46 (1.33, 1.61)	1.00
21- 50	1,801/10,056	2.71 (2.50, 2.92)	1.85
>50	445/1,206	4.61 (4.04, 5.25)	3.16
**Number of positive nodes ($p=8.x10^{-134}$)**			
0	1,088/13,681	1.38 (1.27, 1.50)	1.00
1 to 3	959/5,636	2.73 (2.49, 2.99)	1.97
4 to 9	509/1,683	4.66 (4.13, 5.25)	3.37
10 or more	421/939	7.31 (6.43, 8.31)	5.29
**Tumour grade ($p=7.0x10^{-19}$)**			
Low	15/390	1.16 (0.69, 1.96)	1.00
Medium	374/4,332	1.92 (1.72, 2.15)	1.66
High	2,589/17,217	3.19 (3.04, 3.35)	2.75
**HER2 status ($p=3.7x10^{-21}$)**			
Negative	2,346/15,960	2.48 (2.33, 2.65)	1.00
Positive	633/5,979	1.45 (1.31, 1.61)	0.59
**Breast cancer laterality ($p=1.1x10^{-01}$)**			
Left	1,604/11,513	2.37 (2.20, 2.54)	1.00
Right	1,375/10,427	2.22 (2.06, 2.40)	0.94
**Index of multiple deprivation ($p=2.3x10^{-06}$)**			
<20% (least deprived)	596/4,892	2.09 (1.88, 2.31)	1.00
20- 39%	593/4,766	2.13 (1.93, 2.35)	1.02
40- 59%	609/4,455	2.30 (2.09, 2.54)	1.10
60- 79%	583/4,060	2.43 (2.20, 2.69)	1.17
80+% (most deprived)	597/3,765	2.73 (2.47, 3.02)	1.31
**Region of residence ($p=1.0x10^{-06}$)**			
Eastern	261/2,243	2.14 (1.87, 2.44)	1.00
North West	320/2,810	1.88 (1.65, 2.14)	0.88
Northern & Yorkshire	454/3,170	2.54 (2.28, 2.83)	1.19
Oxford	167/1,230	2.62 (2.21, 3.11)	1.23
South West	429/3,186	2.43 (2.17, 2.72)	1.14
Thames	688/4,755	2.05 (1.85, 2.27)	0.96
Trent	301/2,278	2.52 (2.20, 2.88)	1.18
West Midlands	355/2,264	2.78 (2.47, 3.13)	1.30
**Time since diagnosis (years) ($p=5.1x10^{-01}$)**			
<=1	353/21,940	1.36 (1.21, 1.53)	1.00
1- 2	906/21,376	2.94 (2.70, 3.20)	2.16
2- 3	787/20,225	2.94 (2.69, 3.22)	2.16
3- 4	621/19,164	2.57 (2.33, 2.84)	1.89
4- 5	310/18,265	1.39 (1.22, 1.59)	1.02
**All ER- negative**	2,979/21,940	2.30 (2.16, 2.45)	

Figure S21: Adjusted annual breast cancer mortality rates in women diagnosed with early breast cancer during 2010-2015, with ER-positive or ER-negative disease according to nine characteristics, and time since diagnosis

For each characteristic, rates are adjusted for all the other characteristics shown including time since diagnosis.

HER2: human epidermal growth factor receptor 2. Data available only for period 2010-2015

Tests for interactions in adjusted breast cancer mortality rates are shown in table S7 for all women diagnosed with early breast cancer during 1993-2015 and in table S8 for just the women diagnosed during 2010-2015.

Table S8: Tests for interactions in adjusted annual breast cancer mortality rates between pairs of characteristics in women diagnosed with early breast cancer during 2010-2015, with ER-positive or ER-negative disease

Factors	P value	
	ER-positive	ER-negative
Time since diagnosis x age at diagnosis	$3.6x10^{-03}$	$5.6x10^{-03}$
Time since diagnosis x cancer screen-detected	$2.1x10^{-01}$	$4.3x10^{-03}$
Time since diagnosis x tumour size	$2.6x10^{-01}$	$2.3x10^{-07}$
Time since diagnosis x number of positive nodes	$1.0x10^{-01}$	$3.9x10^{-01}$
Time since diagnosis x tumour grade	$4.7x10^{-12}$	$4.2x10^{-05}$
Time since diagnosis x HER2 status	$4.9x10^{-01}$	$2.8x10^{-01}$
Time since diagnosis x breast cancer laterality	$5.7x10^{-01}$	$6.4x10^{-01}$
Time since diagnosis x index of multiple deprivation	$7.9x10^{-01}$	$9.8x10^{-01}$
Time since diagnosis x region of residence	$2.2x10^{-01}$	$3.5x10^{-01}$
Age at diagnosis x cancer screen-detected	$5.9x10^{-01}$	$8.0x10^{-02}$
Age at diagnosis x tumour size	$4.8x10^{-01}$	$7.2x10^{-01}$
Age at diagnosis x number of positive nodes	$4.0x10^{-01}$	$1.0x10^{+00}$
Age at diagnosis x tumour grade	$4.6x10^{-05}$	$6.7x10^{-01}$
Age at diagnosis x HER2 status	$9.2x10^{-05}$	$1.1x10^{-01}$
Age at diagnosis x breast cancer laterality	$1.4x10^{-01}$	$3.0x10^{-01}$
Age at diagnosis x index of multiple deprivation	$5.5x10^{-01}$	$5.6x10^{-01}$
Age at diagnosis x region of residence	$9.1x10^{-01}$	$7.3x10^{-01}$
Cancer screen-detected x tumour size	$6.6x10^{-01}$	$1.3x10^{-01}$
Cancer screen-detected x number of positive nodes	$1.0x10^{-01}$	$8.3x10^{-01}$
Cancer screen-detected x tumour grade	$8.1x10^{-02}$	$7.0x10^{-01}$
Cancer screen-detected x HER2 status	$7.9x10^{-02}$	$5.5x10^{-01}$
Cancer screen-detected x breast cancer laterality	$9.3x10^{-01}$	$7.5x10^{-03}$
Cancer screen-detected x index of multiple deprivation	$9.7x10^{-01}$	$3.2x10^{-01}$
Cancer screen-detected x region of residence	$5.1x10^{-01}$	$4.1x10^{-01}$
Tumour size x number of positive nodes	$6.8x10^{-02}$	$1.7x10^{-01}$
Tumour size x tumour grade	$1.0x10^{-02}$	$8.3x10^{-01}$
Tumour size x HER2 status	$3.5x10^{-01}$	$6.0x10^{-01}$
Tumour size x breast cancer laterality	$8.6x10^{-01}$	$9.9x10^{-01}$
Tumour size x index of multiple deprivation	$9.4x10^{-01}$	$9.7x10^{-01}$
Tumour size x region of residence	$8.7x10^{-01}$	$4.6x10^{-01}$
Number of positive nodes x tumour grade	$1.9x10^{-06}$	$6.9x10^{-01}$
Number of positive nodes x HER2 status	$4.2x10^{-01}$	$9.3x10^{-01}$
Number of positive nodes x breast cancer laterality	$1.0x10^{+00}$	$8.9x10^{-01}$
Number of positive nodes x index of multiple deprivation	$2.8x10^{-01}$	$9.9x10^{-01}$
Number of positive nodes x region of residence	$1.7x10^{-02}$	$9.3x10^{-01}$
Tumour grade x HER2 status	$1.9x10^{-07}$	$9.4x10^{-01}$
Tumour grade x breast cancer laterality	$5.2x10^{-01}$	$5.3x10^{-01}$
Tumour grade x index of multiple deprivation	$2.6x10^{-02}$	$7.5x10^{-01}$
Tumour grade x region of residence	$1.2x10^{-02}$	$5.1x10^{-01}$
HER2 status x breast cancer laterality	$9.7x10^{-01}$	$5.8x10^{-01}$
HER2 status x index of multiple deprivation	$5.8x10^{-01}$	$5.0x10^{-02}$
HER2 status x region of residence	$5.2x10^{-01}$	$5.3x10^{-01}$
Breast cancer laterality x index of multiple deprivation	$9.8x10^{-01}$	$3.1x10^{-01}$
Breast cancer laterality x region of residence	$9.7x10^{-01}$	$3.0x10^{-01}$
Index of multiple deprivation x region of residence	$8.5x10^{-01}$	$8.2x10^{-01}$

All factors treated as categorical.

HER2: human epidermal growth factor receptor 2. Data available only for period 2010-2015.

Table S9: Numbers of women diagnosed with early breast cancer during 2010-2015 and five-year cumulative breast cancer mortality risks by categories of tumour grade, size and number of positive nodes in women with ER-positive or ER-negative disease. Tables are split by HER2 status, age and screening status

a) HER2-negative and aged <50 years at diagnosis (Figure 8, top left panel)

Tumour characteristic combination			ER-positive			ER-negative		
**Grade**	**Tumour size, mm**	**No. positive nodes**	**Deaths/ Women**	**Person-years at risk**	**5-year cumulative mortality risk, % (95% CI)**	**Deaths/ Women**	**Person-years at risk**	**5-year cumulative mortality risk, % (95% CI)**
Low	1-20	0	3/2669	12,644·7	0·1 (0·0 - 0·4)	0/35	167.2	-
		1-3	2/583	2750·0	0·4 (0·1 - 1·8)	0/4	16.9	-
		4-9	1/47	217·4	2·4 (0·3 - 15·2)	0/0	-	-
		10+	0/12	58·0	-	0/1	5.0	-
	21-50	0	2/460	2175·5	0·5 (0·1 - 2·0)	0/12	58.7	-
		1-3	2/330	1561·6	0·6 (0·1 - 3·0)	0/1	4.6	-
		4-9	0/60	283·8	0·4 (0·0 - 0·7)	0/0	-	-
		10+	1/20	92·9	-	0/0	-	-
	50+	0	0/25	118·9	-	0/3	13.3	-
		1-3	0/28	130·6	-	0/1	5.9	-
		4-9	0/16	77·8	-	0/0	-	-
		10+	0/6	28·4	-	0/0	-	-
Medium	1-20	0	36/4930	23,286·3	0·7 (0·5 - 1·0)	9/205	953.1	4·3 (2·1 - 8·8)
		1-3	25/1995	9393·6	1·3 (0·8 - 2·0)	4/69	314.0	6·5 (2·4 - 17·1)
		4-9	13/266	1242·6	4·8 (2·7 - 8·3)	1/9	37.8	-
		10+	3/70	325·8	4·5 (1·4 - 14·4)	2/4	11.6	-
	21-50	0	28/2262	10,649·4	1·3 (0·8 - 1·9)	10/105	474.5	9·7 (5·3 - 17·4)
		1-3	56/2278	10,697·3	2·5 (1·9 - 3·2)	8/62	269.1	13·7 (7·0 - 25·8)
		4-9	35/619	2868·0	5·7 (4·1 - 7·9)	4/20	83.6	-
		10+	18/198	889·3	9·1 (5·8 - 14·1)	3/8	27.2	-
	50+	0	2/203	955·6	1·2 (0·3 - 4·8)	0/4	20.4	-
		1-3	10/429	2011·2	2·3 (1·1 - 4·5)	1/8	36.6	-
		4-9	16/208	953·2	7·5 (4·6 - 12·0)	2/5	18.5	-
		10+	13/104	462·2	12·5 (7·6 - 20·2)	2/5	14.5	-
High	1-20	0	40/1817	8509·6	2·2 (1·6 - 3·1)	51/1085	5008.3	4·8 (3·6 - 6·3)
		1-3	39/890	4146·6	4·3 (3·1 - 6·0)	34/336	1508.1	10·1 (7·3 - 14·0)
		4-9	17/145	653·2	11·6 (7·3 - 18·2)	13/60	241.5	22·0 (14·1 - 33·5)
		10+	9/45	188·9	19·6 (11·0 - 33·6)	9/26	100.8	-
	21-50	0	85/1618	7467·6	5·3 (4·2 - 6·6)	105/1075	4822.3	9·8 (8·2 - 11·7)
		1-3	113/1412	6441·8	8·0 (6·7 - 9·6)	106/547	2324.2	19·5 (16·7 - 22·7)
		4-9	69/455	2004·6	15·1 (12·3 - 18·6)	46/140	535.7	33·3 (27·4 - 40·0)
		10+	37/168	710·7	22·0 (17·0 - 28·2)	26/51	161.5	53·5 (45·9 - 61·6)
	50+	0	3/57	260·7	5·6 (1·6 - 18·6)	9/49	204.0	18·5 (10·6 - 31·3)
		1-3	20/174	770·7	11·6 (7·5 - 17·7)	23/75	284.9	32·4 (24·5 - 42·1)
		4-9	24/136	580·9	18·0 (12·8 - 24·8)	13/33	107.9	-
		10+	34/87	323·6	39·5 (33·0 - 46·9)	17/32	93.5	-
No. with estimated risk			24,715			3859		
No. in categories with fewer than 40 women, for whom risks were not estimated			107			211		
**Total number of women**			**24,822**			**4070**		

b) HER2-negative, aged 50-70 years at diagnosis and screen-detected (Figure 8, second row, left-hand side)

Tumour characteristic combination			ER-positive			ER-negative		
Grade	Tumour size, mm	No. positive nodes	Deaths/ Women	Person-years at risk	5-year cumulative mortality risk, % (95% CI)	Deaths/ Women	Person-years at risk	5-year cumulative mortality risk, % (95% CI)
Low	1-20	0	25/10,379	48,823·1	0·2 (0·2 - 0·4)	0/97	459·6	0·1 (0·0 - 0·2)
		1-3	4/1289	6069·0	0·3 (0·1 - 0·9)	0/11	54·2	-
		4-9	1/87	408·3	1·3 (0·2 - 8·6)	0/0	-	-
		10+	0/18	84·4	-	0/0	-	-
	21-50	0	4/703	3301·7	0·6 (0·2 - 1·6)	0/13	61·9	-
		1-3	2/311	1457·3	0·7 (0·2 - 2·8)	0/2	7·2	-
		4-9	0/49	231·3	0·0 (0·0 - 0·2)	0/0	-	-
		10+	0/9	40·9	-	0/1	5·2	-
	50+	0	0/25	116·6	-	0/0	-	-
		1-3	0/22	100·7	-	0/0	-	-
		4-9	0/8	39·3	-	0/0	-	-
		10+	0/1	6·3	-	0/0	-	-
Medium	1-20	0	80/15,533	73,006·3	0·5 (0·4 - 0·7)	16/655	3048·2	2·5 (1·5 - 4·1)
		1-3	33/3152	14,760·8	1·1 (0·8 - 1·5)	4/97	453·9	4·2 (1·4 - 11·9)
		4-9	6/318	1479·1	1·9 (0·8 - 4·6)	3/13	56·9	-
		10+	5/95	434·2	5·5 (2·2 - 13·5)	0/3	13·2	-
	21-50	0	31/3109	14,583·4	1·0 (0·7 - 1·4)	3/91	416·7	3·5 (1·1 - 11·3)
		1-3	31/1680	7847·5	1·9 (1·3 - 2·7)	5/52	228·7	10·7 (4·5 - 24·3)
		4-9	19/368	1701·3	5·2 (3·3 - 8·0)	2/8	34·4	-
		10+	17/157	705·9	10·8 (7·1 - 16·3)	3/7	27·9	-
	50+	0	4/169	789·3	2·5 (1·0 - 6·4)	0/2	7·1	-
		1-3	4/183	843·8	2·2 (0·8 - 6·1)	0/4	15·6	-
		4-9	4/71	329·3	5·6 (2·2 - 14·0)	0/1	3·0	-
		10+	5/63	284·9	8·2 (3·7 - 17·7)	1/3	9·0	-
High	1-20	0	51/2912	13,597·3	1·8 (1·3 - 2·4)	40/1199	5553·0	3·4 (2·5 - 4·7)
		1-3	20/706	3270·3	2·9 (1·7 - 4·7)	17/209	932·4	8·2 (5·1 - 13·0)
		4-9	9/111	502·3	7·8 (4·1 - 14·7)	5/36	156·0	-
		10+	5/36	156·1	-	1/11	49·1	-
	21-50	0	29/932	4327·4	3·1 (2·1 - 4·5)	30/339	1532·4	9·0 (6·5 - 12·4)
		1-3	24/606	2792·0	4·1 (2·7 - 6·1)	21/147	633·6	14·5 (9·9 - 21·1)
		4-9	25/189	845·4	13·1 (9·2 - 18·4)	11/44	181·6	25·1 (16·1 - 37·7)
		10+	5/78	350·4	6·9 (2·8 - 16·6)	6/17	61·8	-
	50+	0	0/23	109·4	-	0/9	36·9	-
		1-3	5/49	214·4	11·3 (4·9 - 24·7)	2/16	70·5	-
		4-9	5/24	106·0	-	4/9	37·2	-
		10+	3/17	71·3	-	3/6	18·5	-
No. with estimated risk			43,299			2930		
No. in categories with fewer than 40 women, for whom risks were not estimated			183			172		
**Total number of women**			**43,482**			**3102**		

c) HER2-negative, aged 50-70 years at diagnosis and not screen-detected (Figure 8, third row, left-hand side)

Tumour characteristic combination			ER-positive			ER-negative		
Grade	Tumour size, mm	No. positive nodes	Deaths/ Women	Person-years at risk	5-year cumulative mortality risk, % (95% CI)	Deaths/ Women	Person-years at risk	5-year cumulative mortality risk, % (95% CI)
Low	1-20	0	7/2804	13,151·2	0·3 (0·1 - 0·5)	2/42	195·4	5·0 (1·4 - 17·5)
		1-3	4/590	2758·9	0·6 (0·2 - 1·9)	0/7	32·1	-
		4-9	1/52	239·6	2·2 (0·3 - 13·5)	0/0	-	-
		10+	0/10	45·3	-	0/0	-	-
	21-50	0	2/523	2450·1	0·3 (0·1 - 1·8)	0/24	111·9	-
		1-3	4/314	1473·3	1·2 (0·4 - 3·6)	1/5	25·0	-
		4-9	1/56	260·7	2·1 (0·3 - 16·1)	-	1·3	-
		10+	2/17	77·2	-	-	0·6	-
	50+	0	0/30	142·9	-	0/3	15·8	-
		1-3	2/37	169·2	-	-	0·4	-
		4-9	0/8	39·1	-	1/1	2·8	-
		10+	0/6	28·0	-	0/0	-	-
Medium	1-20	0	52/5828	27,344·8	0·9 (0·7 - 1·2)	12/360	1657·7	3·5 (1·9 - 6·5)
		1-3	40/2029	9472·2	2·0 (1·4 - 2·8)	8/92	411·1	8·9 (4·3 - 18·0)
		4-9	13/269	1249·8	5·0 (2·8 - 8·7)	4/16	69·0	-
		10+	9/83	378·2	10·6 (5·7 - 19·3)	0/4	18·9	-
	21-50	0	78/3049	14,210·8	2·6 (2·0 - 3·3)	20/167	727·3	12·5 (8·3 - 18·5)
		1-3	114/2417	11,150·1	4·7 (3·9 - 5·7)	11/94	408·0	12·5 (6·9 - 21·9)
		4-9	58/661	2979·2	8·8 (6·8 - 11·3)	7/36	151·3	-
		10+	39/275	1210·7	14·3 (10·8 - 18·8)	4/13	52·0	-
	50+	0	5/254	1187·1	1·9 (0·7 - 5·0)	0/11	51·7	-
		1-3	22/420	1923·9	5·3 (3·4 - 8·0)	3/14	56·1	-
		4-9	20/202	906·7	10·1 (6·7 - 14·9)	3/9	36·0	-
		10+	43/189	798·4	22·6 (18·2 - 27·9)	2/4	10·3	-
High	1-20	0	52/1785	8305·3	2·9 (2·2 - 3·9)	77/1159	5226·9	6·8 (5·5 - 8·4)
		1-3	43/728	3325·8	6·0 (4·4 - 8·2)	56/340	1456·2	16·8 (13·3 - 21·0)
		4-9	16/139	619·6	11·8 (7·5 - 18·5)	14/63	257·5	23·1 (15·1 - 34·3)
		10+	7/43	188·0	16·0 (8·1 - 30·3)	8/30	121·1	-
	21-50	0	119/1786	8139·0	6·7 (5·6 - 8·0)	142/1212	5288·2	12·0 (10·3 - 13·8)
		1-3	122/1488	6721·6	8·2 (6·9 - 9·8)	126/588	2414·4	21·9 (19·0 - 25·1)
		4-9	60/446	1962·8	13·4 (10·6 - 16·9)	58/170	621·4	36·0 (30·6 - 42·1)
		10+	47/190	769·1	25·0 (20·1 - 30·7)	46/100	334·0	47·7 (41·9 - 54·0)
	50+	0	6/69	316·5	8·6 (3·8 - 18·9)	20/63	215·9	35·0 (27·0 - 44·4)
		1-3	19/142	616·8	13·7 (9·0 - 20·5)	21/72	266·2	31·2 (23·3 - 41·0)
		4-9	19/88	363·2	22·6 (15·6 - 32·0)	12/37	135·6	-
		10+	27/81	307·4	34·2 (27·3 - 42·3)	28/41	89·5	77·7 (73·9 - 81·3)
No. with estimated risk			27,000			4563		
No. in categories with fewer than 40 women, for whom risks were not estimated			108			214		
**Total number of women**			**27,108**			**4777**		

d) HER2-negative and aged 71+ years at diagnosis (Figure 8, bottom left panel)

Tumour characteristic combination			ER-positive			ER-negative		
**Grade**	**Tumour size, mm**	**No. positive nodes**	**Deaths/ Women**	**Person-years at risk**	**5-year cumulative mortality risk, % (95% CI)**	**Deaths/ Women**	**Person-years at risk**	**5-year cumulative mortality risk, % (95% CI)**
Low	1-20	0	26/2539	11,600·1	1·1 (0·7 - 1·6)	2/40	177·7	5·4 (1·3 - 21·4)
		1-3	7/442	1986·0	1·7 (0·8 - 4·0)	1/7	27·1	-
		4-9	1/31	138·4	-	0/2	8·8	-
		10+	0/11	48·8	-	0/0	-	-
	21-50	0	8/666	3009·2	1·3 (0·6 - 2·7)	2/22	94·9	-
		1-3	12/288	1278·7	4·5 (2·5 - 7·8)	2/7	21·5	-
		4-9	6/45	191·5	12·9 (6·2 - 26·0)	2/3	7·9	-
		10+	2/11	46·8	-	0/1	4·8	-
	50+	0	0/23	100·6	-	0/4	11·9	-
		1-3	1/25	108·2	-	0/0	-	-
		4-9	1/13	48·0	-	0/0	-	-
		10+	4/6	15·2	-	0/0	-	-
Medium	1-20	0	90/5928	27,016·1	1·6 (1·3 - 2·0)	14/331	1455·5	4·3 (2·5 - 7·5)
		1-3	52/1476	6627·6	3·7 (2·8 - 4·9)	9/82	352·3	10·9 (5·5 - 20·9)
		4-9	16/182	801·5	8·9 (5·4 - 14·6)	4/16	57·6	-
		10+	8/68	274·7	13·5 (7·1 - 25·1)	2/6	15·2	-
	21-50	0	120/3702	16,536·2	3·4 (2·8 - 4·1)	31/250	1031·8	13·2 (9·6 - 18·0)
		1-3	161/2140	9334·2	7·9 (6·8 - 9·1)	34/127	488·5	28·3 (22·4 - 35·4)
		4-9	80/568	2404·8	14·5 (12·0 - 17·5)	17/47	163·7	39·5 (30·7 - 49·8)
		10+	59/242	973·0	25·2 (20·9 - 30·1)	9/20	55·1	-
	50+	0	13/253	1091·0	5·3 (3·0 - 9·2)	7/23	83·6	-
		1-3	31/283	1225·6	11·3 (8·1 - 15·6)	5/17	63·1	-
		4-9	32/140	578·8	22·8 (17·6 - 29·3)	3/8	21·1	-
		10+	42/129	476·0	34·3 (28·6 - 40·8)	12/18	46·7	-
High	1-20	0	68/1250	5539·5	5·7 (4·4 - 7·2)	87/729	3016·3	12·8 (10·5 - 15·5)
		1-3	44/435	1886·9	10·4 (7·8 - 13·9)	39/197	765·6	21·6 (16·8 - 27·5)
		4-9	14/81	327·4	17·8 (11·0 - 28·0)	16/46	156·1	37·8 (28·4 - 49·1)
		10+	6/27	107·7	-	11/20	56·0	-
	21-50	0	139/1487	6384·6	9·9 (8·5 - 11·5)	193/917	3408·6	23·6 (21·2 - 26·2)
		1-3	185/1084	4392·2	18·2 (16·1 - 20·5)	168/504	1674·1	37·9 (34·8 - 41·2)
		4-9	90/373	1461·7	25·4 (21·7 - 29·6)	97/193	572·6	55·1 (51·4 - 58·8)
		10+	55/148	542·1	38·2 (32·7 - 44·2)	65/101	254·2	70·5 (67·6 - 73·5)
	50+	0	11/90	376·4	13·2 (7·9 - 21·9)	37/93	268·8	48·1 (42·1 - 54·5)
		1-3	35/148	563·8	25·4 (19·8 - 32·4)	43/86	227·1	59·0 (53·4 - 64·7)
		4-9	41/100	331·5	44·5 (38·6 - 50·8)	32/48	107·0	76·0 (72·4 - 79·4)
		10+	30/68	233·2	45·4 (37·9 - 53·5)	37/48	89·3	85·7 (84·0 - 87·2)
No. with estimated risk			24,355			3839		
No. in categories with fewer than 40 women, for whom risks were not estimated			147			174		
**Total number of women**			**24,502**			**4013**		

e) HER2-positive and aged <50 years at diagnosis (Figure 8, top right panel)

Tumour characteristic combination			ER-positive			ER-negative		
**Grade**	**Tumour size, mm**	**No. positive nodes**	**Deaths/ Women**	**Person-years at risk**	**5-year cumulative mortality risk, % (95% CI)**	**Deaths/ Women**	**Person-years at risk**	**5-year cumulative mortality risk, % (95% CI)**
Low	1-20	0	0/74	353·1	0·0 (0·0 - 0·2)	0/1	4·9	-
		1-3	0/19	91·9	-	0/0	-	-
		4-9	0/3	14·5	-	0/0	-	-
		10+	0/1	6·9	-	0/0	-	-
	21-50	0	0/18	83·1	-	0/1	2·5	-
		1-3	0/9	40·2	-	0/2	6·2	-
		4-9	0/4	18·5	-	0/0	-	-
		10+	0/0	-	-	0/0	-	-
	50+	0	0/0	-	-	0/0	-	-
		1-3	0/2	10·8	-	0/0	-	-
		4-9	0/0	-	-	0/0	-	-
		10+	0/0	-	-	0/0	-	-
Medium	1-20	0	3/601	2847·3	0·5 (0·1 - 1·9)	1/104	486·2	1·4 (0·2 - 8·3)
		1-3	1/260	1227·7	0·4 (0·0 - 5·3)	3/39	184·9	-
		4-9	2/47	220·2	3·5 (0·7 - 17·3)	0/6	25·7	-
		10+	0/16	76·4	-	0/3	14·8	-
	21-50	0	3/236	1117·7	1·2 (0·3 - 4·4)	1/29	137·6	-
		1-3	4/261	1227·2	1·5 (0·4 - 4·8)	2/45	208·8	5·0 (1·2 - 20·0)
		4-9	4/87	407·4	4·0 (1·3 - 12·0)	2/14	64·8	-
		10+	4/32	142·4	-	0/4	16·2	-
	50+	0	1/14	63·2	-	0/2	9·9	-
		1-3	1/39	178·0	-	0/7	30·8	-
		4-9	1/24	114·3	-	0/4	19·2	-
		10+	4/17	73·5	-	0/1	3·9	-
High	1-20	0	10/622	2935·3	1·5 (0·8 - 3·1)	10/337	1572·5	3·1 (1·6 - 6·1)
		1-3	6/333	1570·6	1·9 (0·7 - 5·0)	13/174	799·4	7·3 (4·0 - 13·1)
		4-9	2/63	296·3	3·3 (0·6 - 18·3)	2/38	170·8	-
		10+	3/17	73·9	-	2/17	76·1	-
	21-50	0	13/485	2280·8	2·8 (1·5 - 4·9)	15/253	1173·2	5·9 (3·4 - 10·1)
		1-3	21/519	2413·1	4·1 (2·5 - 6·8)	24/214	962·7	11·0 (7·2 - 16·6)
		4-9	15/186	846·6	7·9 (4·5 - 13·6)	15/95	411·3	15·6 (9·8 - 24·3)
		10+	11/82	365·4	13·2 (7·1 - 23·8)	13/52	216·1	24·9 (16·3 - 36·8)
	50+	0	0/16	76·4	-	1/8	33·8	-
		1-3	3/58	266·7	5·0 (1·2 - 19·5)	5/32	137·4	-
		4-9	5/43	192·1	10·7 (3·9 - 27·2)	6/18	71·1	-
		10+	6/24	98·8	-	8/21	77·1	-
No. with estimated risk			3957			1274		
No. in categories with fewer than 40 women, for whom risks were not estimated			255			248		
**Total number of women**			**4212**			**1522**		

f) HER2-positive, aged 50-70 years at diagnosis and screen-detected (Figure 8, second row, right-hand side)

Tumour characteristic combination			ER-positive			ER-negative		
**Grade**	**Tumour size, mm**	**No. positive nodes**	**Deaths/ Women**	**Person-years at risk**	**5-year cumulative mortality risk, % (95% CI)**	**Deaths/ Women**	**Person-years at risk**	**5-year cumulative mortality risk, % (95% CI)**
Low	1-20	0	1/213	990·3	0·7 (0·1 - 4·1)	0/13	61·9	-
		1-3	0/34	161·0	-	0/0	-	-
		4-9	0/1	6·7	-	0/0	-	-
		10+	0/2	8·1	-	0/0	-	-
	21-50	0	0/25	117·3	-	0/0	-	-
		1-3	0/10	45·6	-	0/0	-	-
		4-9	0/4	17·2	-	0/0	-	-
		10+	0/0	-	-	0/0	-	-
	50+	0	0/0	-	-	0/0	-	-
		1-3	0/0	-	-	0/0	-	-
		4-9	0/1	5·7	-	0/0	-	-
		10+	0/0	-	-	0/0	-	-
Medium	1-20	0	14/1245	5841·7	1·1 (0·7 - 1·9)	3/203	950·2	1·4 (0·4 - 4·9)
		1-3	4/272	1274·0	1·4 (0·5 - 4·0)	1/48	225·2	1·6 (0·1 - 23·0)
		4-9	1/35	162·1	-	0/4	16·2	-
		10+	2/15	66·9	-	0/1	2·5	-
	21-50	0	1/242	1132·3	0·6 (0·1 - 3·5)	0/26	121·9	-
		1-3	4/143	667·0	2·5 (0·8 - 7·2)	2/19	83·4	-
		4-9	3/37	172·2	-	0/4	15·5	-
		10+	1/25	118·1	-	0/1	3·3	-
	50+	0	0/11	50·5	-	0/3	15·8	-
		1-3	1/9	43·8	-	1/3	9·1	-
		4-9	0/6	28·0	-	1/2	7·8	-
		10+	0/1	6·6	-	0/0	-	-
High	1-20	0	13/840	3929·5	1·6 (0·9 - 2·8)	10/460	2138·7	2·1 (1·1 - 4·2)
		1-3	4/208	966·5	1·9 (0·6 - 5·9)	2/112	522·0	1·9 (0·3 - 11·9)
		4-9	2/33	152·3	-	1/20	94·1	-
		10+	1/15	68·3	-	0/8	38·6	-
	21-50	0	8/272	1253·8	2·9 (1·4 - 5·9)	6/138	635·7	4·5 (2·0 - 10·1)
		1-3	5/193	901·3	2·7 (1·0 - 7·3)	6/76	350·1	7·3 (3·1 - 17·0)
		4-9	5/62	282·1	7·5 (3·0 - 18·6)	2/26	116·4	-
		10+	2/24	106·6	-	2/9	39·1	-
	50+	0	1/9	38·4	-	0/7	31·8	-
		1-3	0/14	65·0	-	0/7	31·7	-
		4-9	0/6	27·9	-	0/3	15·3	-
		10+	1/6	24·0	-	0/4	15·9	-
No. with estimated risk			3690			1037		
No. in categories with fewer than 40 women, for whom risks were not estimated			323			160		
**Total number of women**			**4013**			**1197**		

g) HER2-positive, aged 50-70 years at diagnosis and not screen-detected (Figure 8, third row, right-hand side)

Tumour characteristic combination			ER-positive			ER-negative		
**Grade**	**Tumour size, mm**	**No. positive nodes**	**Deaths/ Women**	**Person-years at risk**	**5-year cumulative mortality risk, % (95% CI)**	**Deaths/ Women**	**Person-years at risk**	**5-year cumulative mortality risk, % (95% CI)**
Low	1-20	0	0/75	353·9	0·0 (0·0 - 0·1)	1/9	39·5	-
		1-3	0/19	90·2	-	0/1	4·6	-
		4-9	0/1	4·8	-	0/0	-	-
		10+	0/0	-	-	0/0	-	-
	21-50	0	0/11	50·7	-	0/1	4·8	-
		1-3	1/9	38·6	-	0/1	2·5	-
		4-9	1/5	21·3	-	0/0	-	-
		10+	0/0	-	-	0/0	-	-
	50+	0	0/1	5·5	-	0/0	-	-
		1-3	0/1	6·7	-	0/0	-	-
		4-9	0/0	-	-	0/0	-	-
		10+	0/0	-	-	0/0	-	-
Medium	1-20	0	5/545	2546·3	0·9 (0·3 - 2·6)	5/145	665·0	3·4 (1·3 - 8·7)
		1-3	3/205	955·8	1·3 (0·3 - 6·6)	1/45	208·3	2·4 (0·2 - 24·2)
		4-9	2/34	154·4	-	0/8	34·9	-
		10+	6/17	76·2	-	0/2	8·9	-
	21-50	0	6/322	1501·6	1·7 (0·7 - 4·4)	2/52	236·0	4·1 (0·9 - 17·5)
		1-3	13/231	1058·2	5·6 (3·1 - 9·8)	1/39	180·2	-
		4-9	5/77	348·7	6·9 (2·8 - 16·4)	5/18	68·1	-
		10+	8/43	184·7	17·6 (9·3 - 31·8)	3/7	27·8	-
	50+	0	2/13	53·7	-	0/2	8·1	-
		1-3	0/26	121·2	-	3/8	34·2	-
		4-9	1/19	87·5	-	0/2	9·6	-
		10+	2/12	48·3	-	1/6	21·5	-
High	1-20	0	12/549	2566·5	2·3 (1·3 - 4·1)	15/410	1887·1	3·8 (2·2 - 6·6)
		1-3	6/237	1105·4	2·5 (0·9 - 6·8)	13/180	812·8	7·1 (3·9 - 12·9)
		4-9	5/59	270·8	7·8 (2·9 - 20·0)	6/45	199·1	13·5 (6·1 - 28·2)
		10+	3/23	104·0	-	3/21	88·1	-
	21-50	0	18/543	2508·3	3·4 (2·0 - 5·6)	32/381	1692·1	8·5 (5·9 - 12·0)
		1-3	33/413	1866·0	8·0 (5·6 - 11·2)	33/289	1261·4	11·7 (8·3 - 16·4)
		4-9	16/169	758·6	9·5 (5·7 - 15·5)	26/117	486·0	22·3 (16·3 - 30·1)
		10+	18/82	340·6	22·2 (15·5 - 31·4)	19/69	272·2	27·6 (19·7 - 37·8)
	50+	0	1/20	91·0	-	0/16	72·6	-
		1-3	3/35	152·5	-	7/35	147·0	-
		4-9	4/30	138·6	-	7/22	81·4	-
		10+	8/30	116·5	-	11/20	63·0	-
No. with estimated risk			3550			1733		
No. in categories with fewer than 40 women, for whom risks were not estimated			306			218		
**Total number of women**			**3856**			**1951**		

h) HER2-positive and aged 71+ years at diagnosis (Figure 8, bottom right panel)

Tumour characteristic combination			ER-positive			ER-negative		
**Grade**	**Tumour size, mm**	**No. positive nodes**	**Deaths/ Women**	**Person-years at risk**	**5-year cumulative mortality risk, % (95% CI)**	**Deaths/ Women**	**Person-years at risk**	**5-year cumulative mortality risk, % (95% CI)**
Low	1-20	0	1/50	225·9	2·5 (0·4 - 15·5)	0/5	21·7	-
		1-3	0/15	68·5	-	0/1	5·3	-
		4-9	0/0	-	-	0/0	-	-
		10+	0/0	-	-	0/0	-	-
	21-50	0	2/16	64·5	-	0/1	2·8	-
		1-3	0/12	53·3	-	0/2	10·0	-
		4-9	1/2	5·1	-	0/0	-	-
		10+	0/0	-	-	0/0	-	-
	50+	0	0/0	-	-	0/0	-	-
		1-3	0/0	-	-	0/0	-	-
		4-9	0/0	-	-	0/0	-	-
		10+	0/0	-	-	0/0	-	-
Medium	1-20	0	7/376	1706·8	2·0 (0·9 - 4·5)	4/83	366·1	5·2 (1·9 - 13·6)
		1-3	7/95	425·2	7·8 (3·8 - 15·7)	4/24	101·9	-
		4-9	1/23	105·9	-	0/6	28·2	-
		10+	0/5	22·7	-	0/1	1·9	-
	21-50	0	15/246	1080·0	6·5 (4·0 - 10·6)	2/59	248·4	4·6 (1·1 - 18·2)
		1-3	16/160	687·7	10·5 (6·5 - 16·8)	5/25	101·5	-
		4-9	9/54	221·2	17·6 (10·1 - 29·9)	0/8	31·0	-
		10+	7/26	103·3	-	3/8	30·7	-
	50+	0	2/18	79·9	-	0/1	2·4	-
		1-3	4/19	81·3	-	0/2	8·2	-
		4-9	4/10	39·2	-	2/4	8·8	-
		10+	5/14	55·6	-	2/2	5·7	-
High	1-20	0	18/286	1274·6	6·3 (3·9 - 10·3)	23/225	946·4	10·8 (7·2 - 16·0)
		1-3	11/101	438·3	10·8 (5·8 - 19·5)	17/86	329·6	21·4 (14·5 - 30·8)
		4-9	7/28	106·5	-	9/24	86·0	-
		10+	1/9	38·9	-	5/16	55·5	-
	21-50	0	20/320	1398·9	6·4 (4·0 - 10·2)	30/263	1064·7	12·6 (8·6 - 18·1)
		1-3	48/270	1104·8	18·8 (14·8 - 23·7)	51/207	739·2	27·8 (22·9 - 33·6)
		4-9	27/109	432·5	25·8 (19·5 - 33·6)	38/96	317·5	43·4 (36·8 - 50·6)
		10+	20/59	200·1	37·7 (29·6 - 47·2)	34/60	178·2	59·4 (53·7 - 65·1)
	50+	0	4/12	47·5	-	6/18	60·3	-
		1-3	5/25	92·9	-	13/34	113·1	-
		4-9	9/20	72·3	-	12/25	73·5	-
		10+	8/18	54·7	-	16/27	69·5	-
No. with estimated risk			2126			1079		
No. in categories with fewer than 40 women, for whom risks were not estimated			272			234		
**Total number of women**			**2398**			**1313**		

Table S10: Distribution of cumulative five-year breast cancer mortality risks for women diagnosed with early breast cancer during 2010-2015

5-year cumulative risk of breast cancer mortality (%)	Number of women	Percentage of women*
<1	52257	34.2
<3	96085	62.8
<5	112814	73.7
<10	134655	88.0
≥10	18351	12.0
≥20	6962	4.6
≥40	1085	0.7
≥60	238	0.2

*Percentages based on the 153,006 women (132,692 with ER-positive and 20,314 with ER-negative disease) out of a total of 156,338 registered with early invasive breast cancer during 2010-2015 for whom cumulative five-year risks could be estimated. See table S9 for details.

Breast cancer mortality, square root scale (to accompany figure 8)

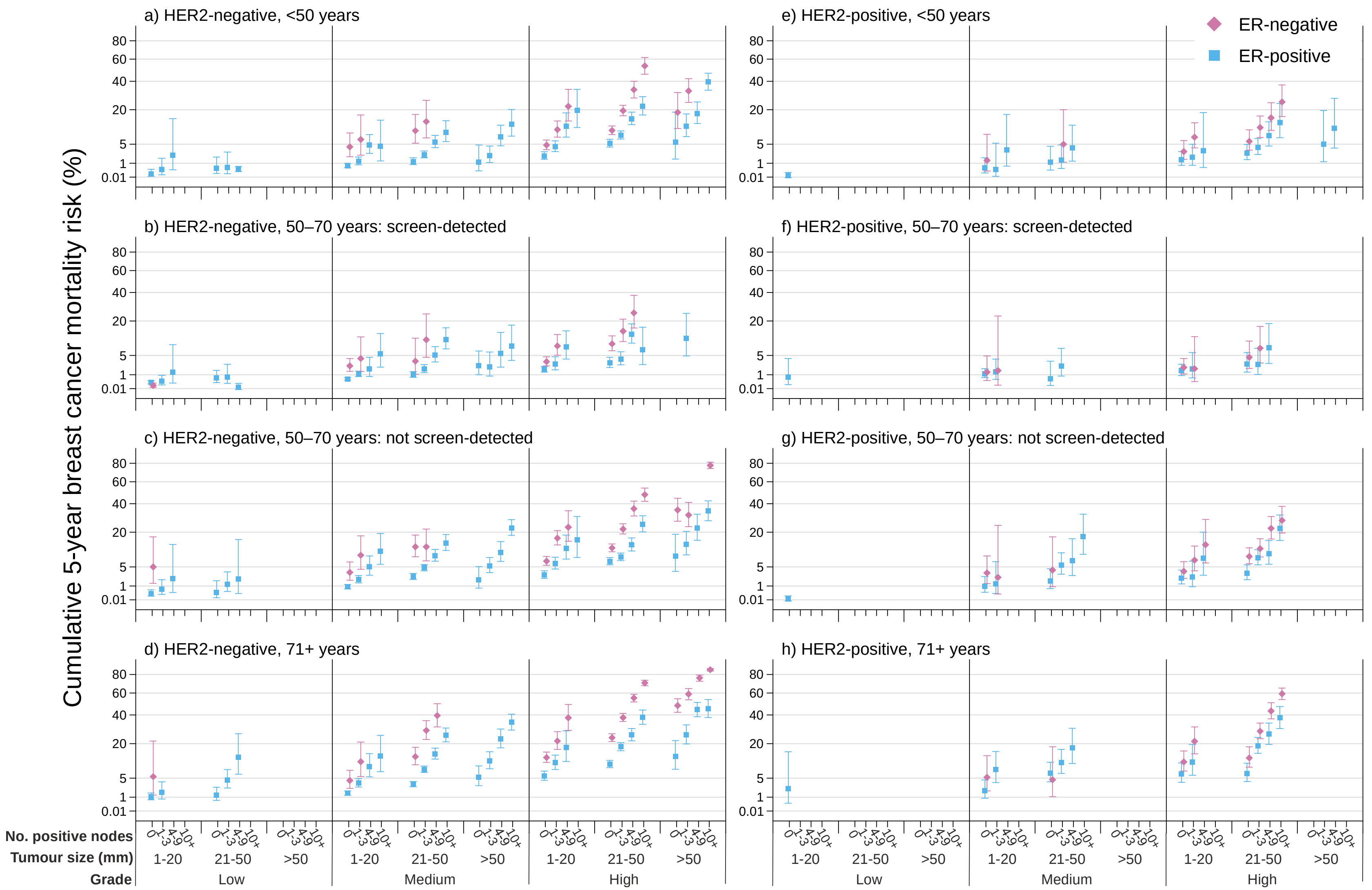

Figure S22: Cumulative five-year breast cancer mortality risks in 156,338 women with early breast cancer diagnosed during 2010-2015 by categories of tumour grade, size and number of positive nodes in women with ER-positive or ER-negative disease. Figures are split by HER2 status, age and screening status.

Y-axes are plotted using square root scale. Vertical lines are 95% confidence intervals. Points are plotted for groups of women with data on at least 40 women and include 153,006/156,338 (97.9%) of the women.

Other Causes of Mortality

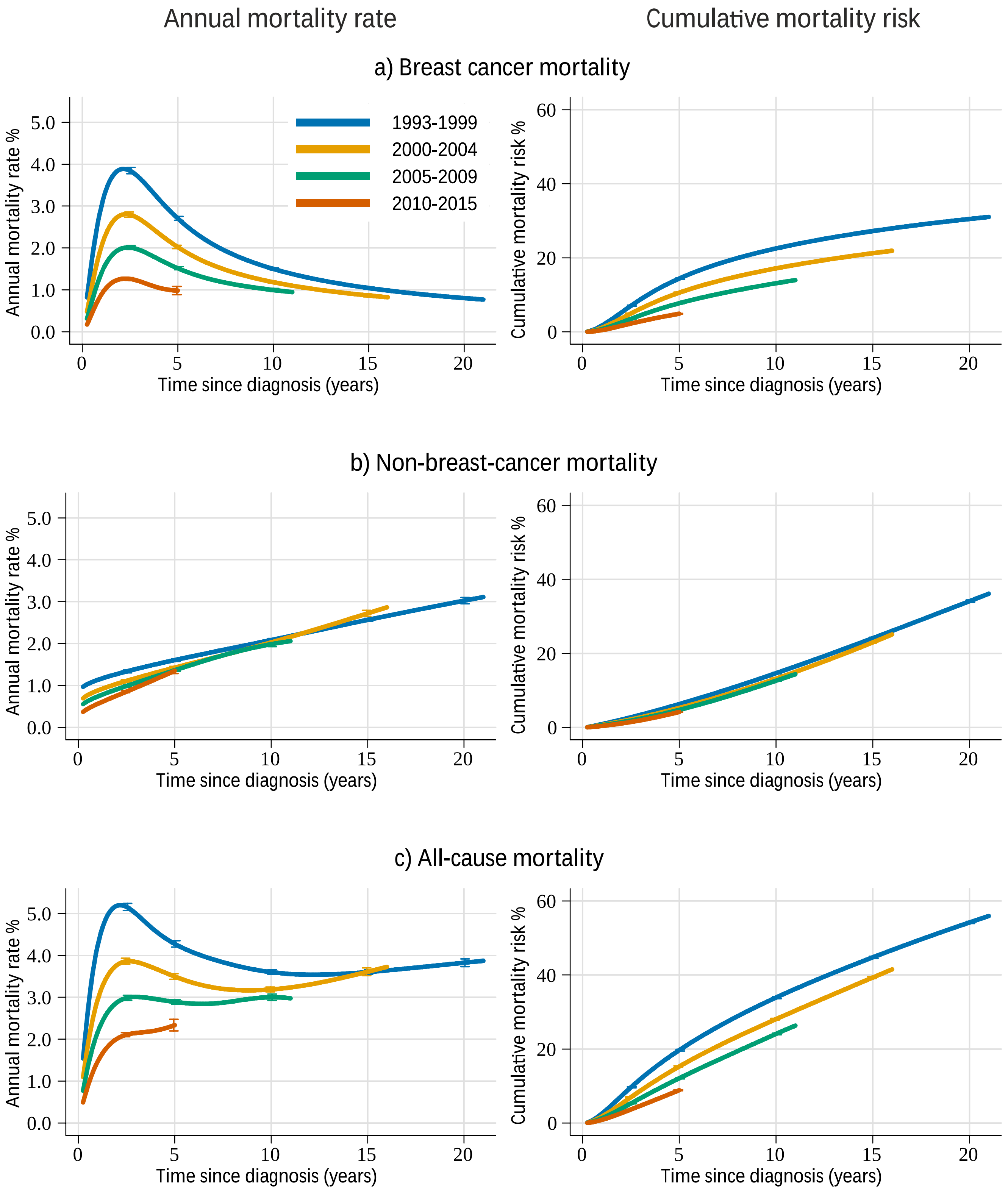

Figure S23: Crude annual rates and cumulative risks of (a) breast cancer mortality (b) non-breast-cancer mortality and (c) all-cause mortality in 512,447 women with early breast cancer by time since diagnosis according to calendar period of diagnosis

Vertical lines are 95% confidence intervals.

All-cause mortality (to accompany figure 1)

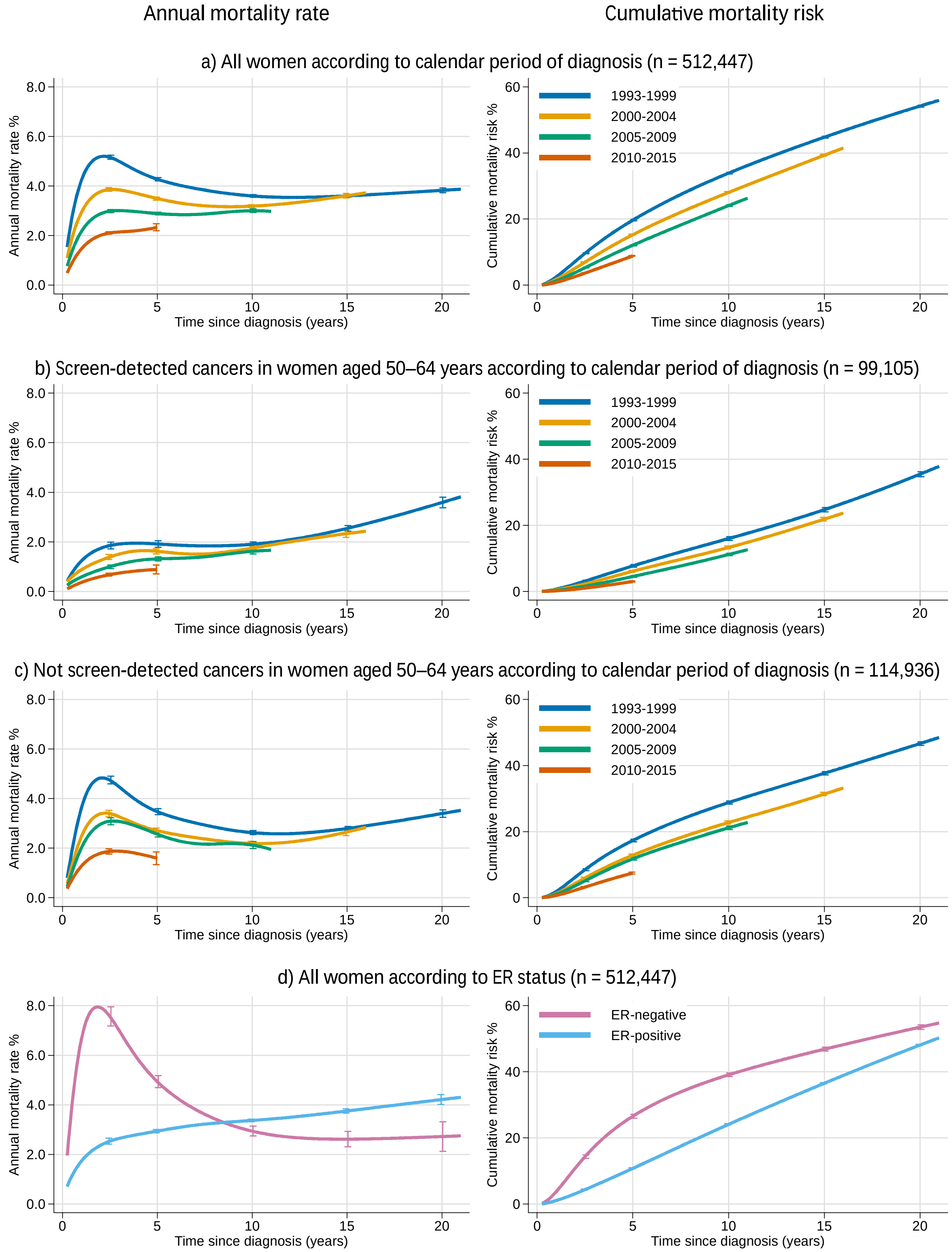

Figure S24: Crude annual all-cause mortality rates and cumulative all-cause mortality risks in 512,447 women with early breast cancer by time since diagnosis according to calendar period of diagnosis: (a) for all women; (b and c) for women aged 50-64 years (who would all have been eligible for screening) according to whether or not their cancer was screen-detected; and (d) according to ER status

Vertical lines are 95% confidence intervals.

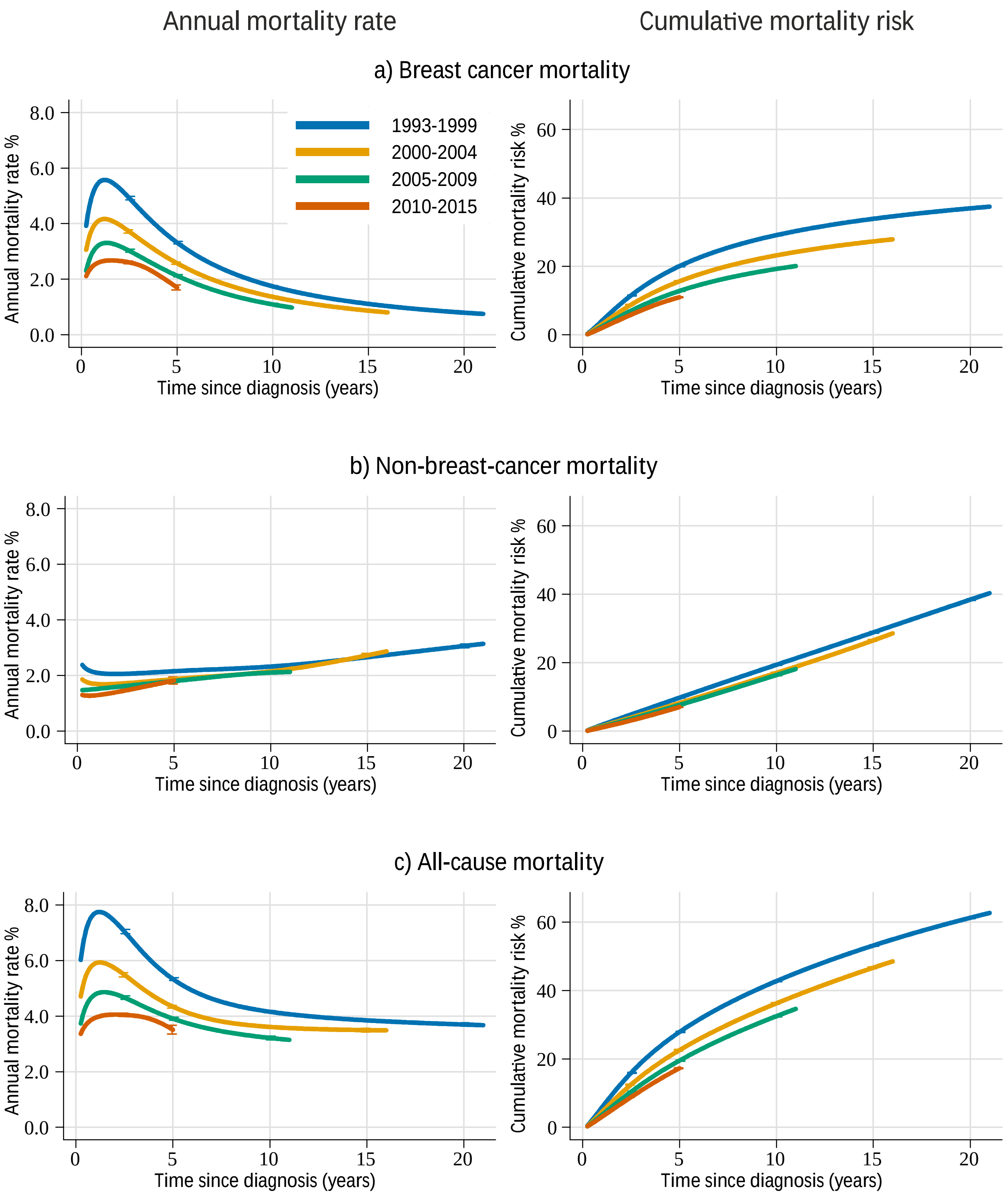

Figure S25: Crude annual rates and cumulative risks of (a) breast cancer mortality (b) non-breast-cancer mortality and (c) all-cause mortality in 693,362 women, including 512,447 women with early breast cancer, 138,911 with probable metastatic disease, and 42,004 recorded as receiving neoadjuvant therapy, by time since diagnosis according to calendar period of diagnosis

Vertical lines are 95% confidence intervals.

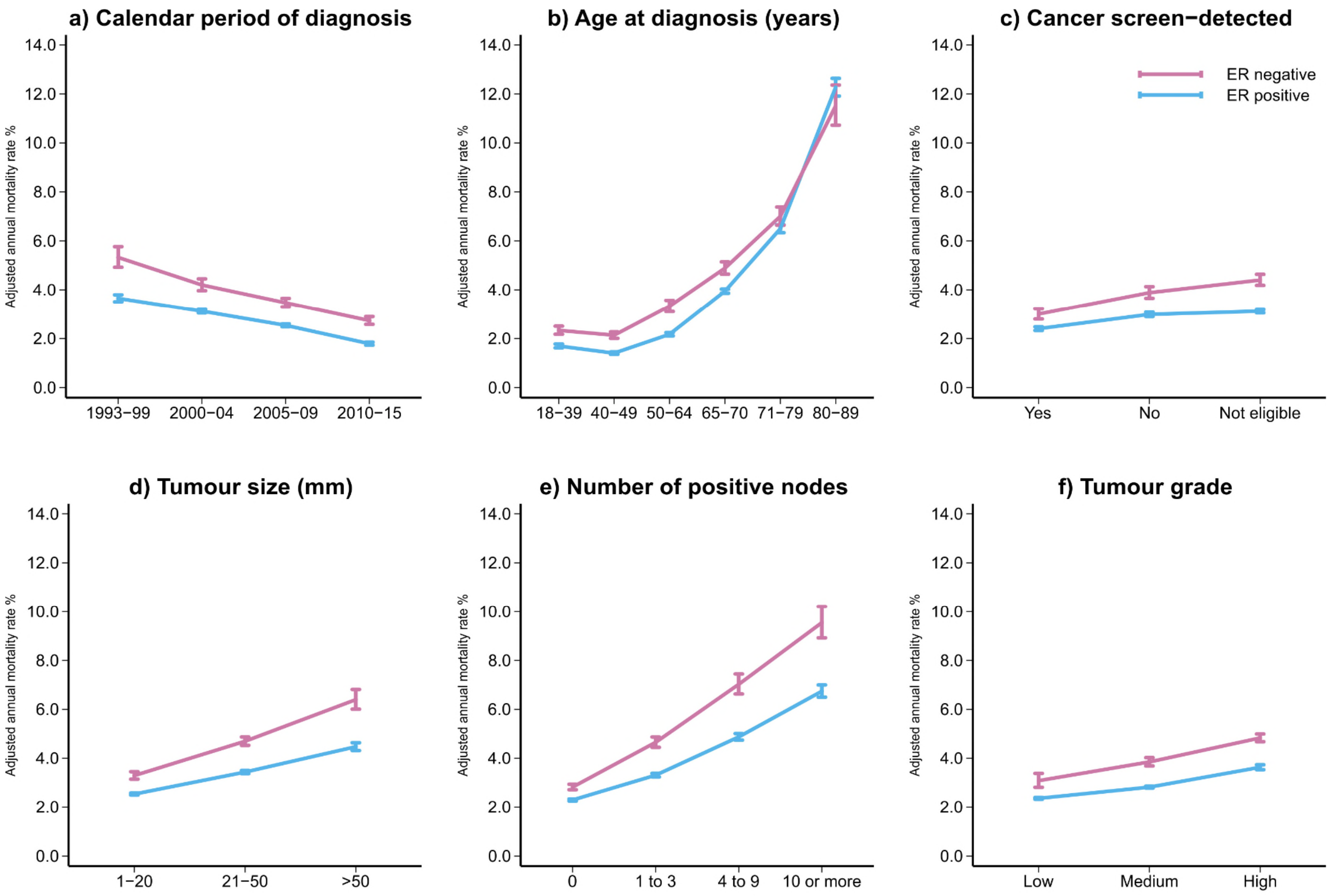

Figure S26: Adjusted annual all-cause mortality rates in women with early breast cancer with ER-positive or ER-negative disease, by various characteristics

For each characteristic, rates are adjusted for all the other characteristics shown and also for time since diagnosis, breast cancer laterality, index of multiple deprivation and region of residence. Further details are in figure S28.
Vertical lines are 95% confidence intervals

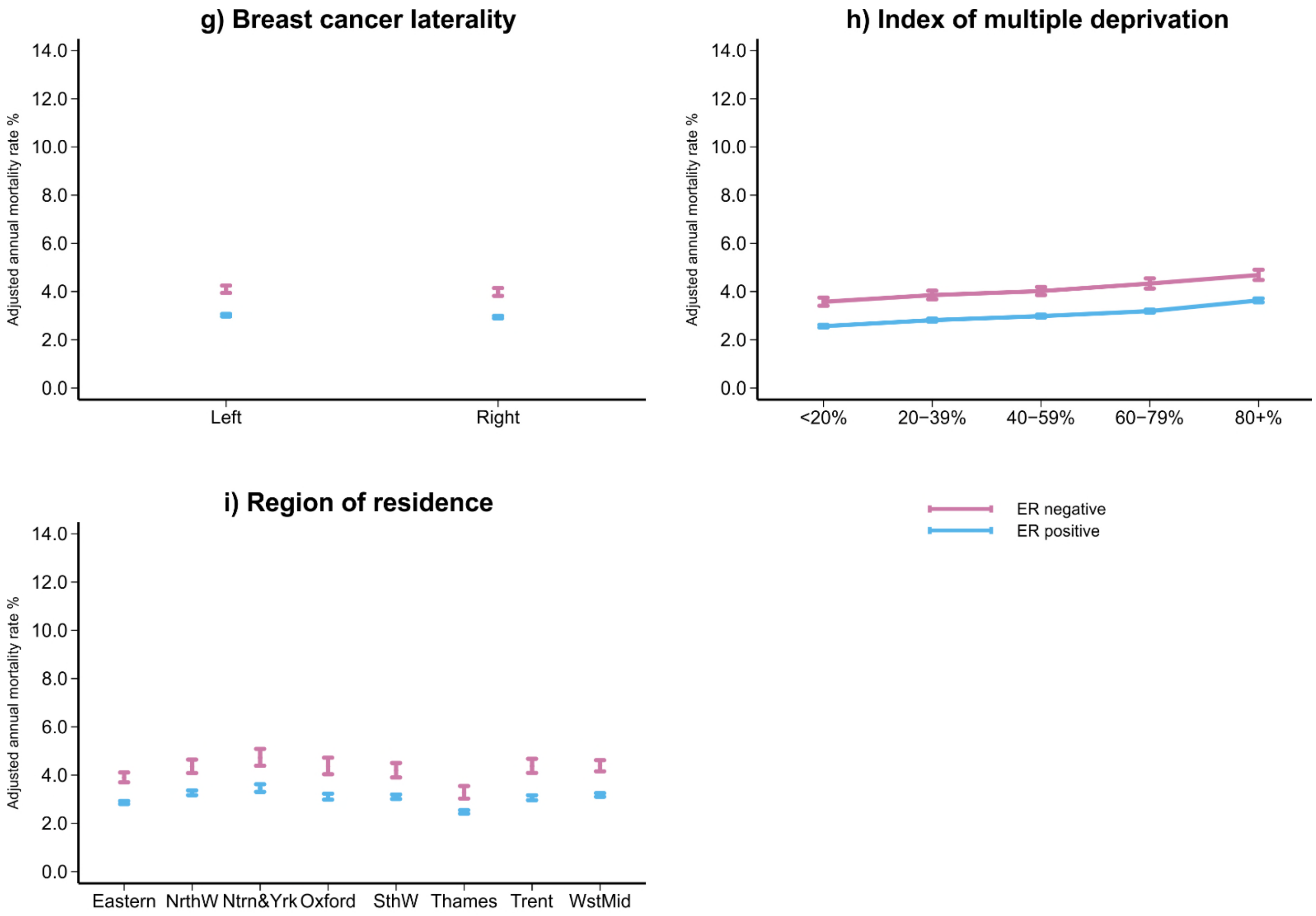

Figure S27: Adjusted annual all-cause mortality rates in women with early breast cancer with ER-positive or ER-negative disease, by breast cancer laterality, index of multiple deprivation and region of residence

For each characteristic, rates are adjusted for all the other characteristics shown and also for the characteristics in Figure S26 and for time since diagnosis.

Further details are in figure S28.

Vertical lines are 95% confidence intervals.

ER-positive

Characteristic	Deaths/ Women	Adjusted annual mortality rate % (95% CI)	Rate ratio
**Calendar period of diagnosis ($p=5.2x10^{-31}$)**			
1993- 1999	46,656/86,965	3.65 (3.51, 3.80)	1.00
2000- 2004	36,903/93,781	3.12 (3.05, 3.20)	0.86
2005- 2009	25,211/106,484	2.54 (2.48, 2.60)	0.70
2010- 2015	9,372/134,397	1.79 (1.73, 1.85)	0.49
**Age at diagnosis (years) ($p=1.x10^{-107}$)**			
18- 39	4,648/20,157	1.70 (1.62, 1.78)	1.00
40- 49	12,195/73,435	1.40 (1.35, 1.45)	0.83
50- 64	35,431/177,712	2.17 (2.11, 2.24)	1.28
65- 70	19,340/64,589	3.94 (3.86, 4.03)	2.33
71- 79	31,295/60,749	6.50 (6.34, 6.66)	3.83
80- 89	15,233/24,984	12.27 (11.91, 12.64)	7.24
**Cancer screen- detected ($p=3.5x10^{-72}$)**			
Eligible: screen- detected	15,357/114,900	2.40 (2.33, 2.48)	1.00
Eligible: not screen- detected	25,868/104,493	2.99 (2.90, 3.08)	1.24
Not eligible for screening	76,918/202,235	3.12 (3.05, 3.19)	1.30
**Tumour size (mm) ($p=3.x10^{-139}$)**			
1- 20	57,690/257,689	2.54 (2.49, 2.58)	1.00
21- 50	53,407/147,684	3.44 (3.37, 3.50)	1.36
>50	7,045/16,254	4.47 (4.31, 4.64)	1.76
**Number of positive nodes ($p=2.1x10^{-97}$)**			
0	52,026/242,061	2.29 (2.25, 2.34)	1.00
1 to 3	40,911/127,078	3.31 (3.24, 3.38)	1.44
4 to 9	16,997/37,339	4.87 (4.74, 5.01)	2.12
10 or more	8,208/15,149	6.74 (6.50, 7.00)	2.94
**Tumour grade ($p=6.0x10^{-54}$)**			
Low	21,430/93,798	2.36 (2.32, 2.40)	1.00
Medium	61,282/223,802	2.82 (2.78, 2.86)	1.20
High	35,431/104,027	3.63 (3.53, 3.73)	1.54
**Breast cancer laterality ($p=6.7x10^{-05}$)**			
Left	61,288/215,990	3.02 (2.97, 3.07)	1.00
Right	56,855/205,638	2.94 (2.89, 2.99)	0.97
**Index of multiple deprivation ($p=4.x10^{-135}$)**			
<20% (least deprived)	23,496/98,369	2.57 (2.51, 2.62)	1.00
20- 39%	25,853/97,503	2.82 (2.76, 2.87)	1.10
40- 59%	25,289/89,098	2.98 (2.93, 3.04)	1.16
60- 79%	23,550/76,540	3.19 (3.13, 3.25)	1.24
80+% (most deprived)	19,954/60,117	3.64 (3.56, 3.72)	1.42
**Region of residence ($p=9.6x10^{-67}$)**			
Eastern	12,411/47,477	2.86 (2.80, 2.93)	1.00
North West	17,725/57,846	3.27 (3.17, 3.37)	1.14
Northern & Yorkshire	14,477/52,807	3.46 (3.30, 3.62)	1.21
Oxford	6,293/25,492	3.11 (2.98, 3.23)	1.08
South West	19,678/68,253	3.10 (3.01, 3.20)	1.08
Thames	25,213/88,463	2.47 (2.40, 2.55)	0.86
Trent	8,497/34,691	3.06 (2.96, 3.16)	1.07
West Midlands	13,847/46,597	3.18 (3.10, 3.25)	1.11
**Time since diagnosis (years) ($p=1.4x10^{-29}$)**			
<=1	4,081/421,628	1.21 (1.14, 1.29)	1.00
1- 2	8,497/416,990	1.97 (1.85, 2.10)	1.63
2- 3	10,137/408,456	2.49 (2.38, 2.62)	2.06
3- 4	11,362/398,301	2.97 (2.85, 3.09)	2.45
4- 5	10,401/386,920	2.89 (2.79, 2.98)	2.38
5- 10	37,029/251,543	3.19 (3.14, 3.24)	2.63
10- 15	23,873/213,256	3.91 (3.82, 4.00)	3.23
15- 20	11,073/107,488	4.72 (4.49, 4.95)	3.89
20- 21	1,686/40,078	5.53 (5.10, 6.01)	4.56
**All ER- positive**	118,144/421,628	2.98 (2.93, 3.03)	

ER-negative

Characteristic	Deaths/ Women	Adjusted annual mortality rate % (95% CI)	Rate ratio
**Calendar period of diagnosis ($p=5.0x10^{-18}$)**			
1993- 1999	15,549/26,389	5.33 (4.92, 5.76)	1.00
2000- 2004	9,999/21,044	4.20 (3.96, 4.45)	0.79
2005- 2009	7,987/21,444	3.47 (3.30, 3.65)	0.65
2010- 2015	4,214/21,940	2.74 (2.58, 2.90)	0.51
**Age at diagnosis (years) ($p=4.x10^{-106}$)**			
18- 39	2,619/7,965	2.33 (2.18, 2.51)	1.00
40- 49	5,540/17,872	2.14 (2.01, 2.27)	0.92
50- 64	12,644/36,329	3.33 (3.11, 3.57)	1.43
65- 70	4,941/10,820	4.89 (4.64, 5.14)	2.09
71- 79	7,796/12,035	7.00 (6.64, 7.38)	3.00
80- 89	4,207/5,795	11.51 (10.72, 12.36)	4.93
**Cancer screen- detected ($p=1.4x10^{-18}$)**			
Eligible: screen- detected	3,150/13,339	3.00 (2.80, 3.22)	1.00
Eligible: not screen- detected	11,061/28,933	3.88 (3.65, 4.13)	1.29
Not eligible for screening	23,538/48,544	4.40 (4.18, 4.63)	1.47
**Tumour size (mm) ($p=2.3x10^{-48}$)**			
1- 20	13,331/42,849	3.29 (3.15, 3.45)	1.00
21- 50	20,892/42,575	4.70 (4.53, 4.87)	1.43
>50	3,526/5,393	6.40 (6.01, 6.81)	1.94
**Number of positive nodes ($p=3.3x10^{-67}$)**			
0	13,919/46,397	2.82 (2.71, 2.94)	1.00
1 to 3	13,550/28,585	4.65 (4.45, 4.87)	1.65
4 to 9	6,414/10,385	7.02 (6.63, 7.45)	2.49
10 or more	3,865/5,448	9.55 (8.92, 10.21)	3.38
**Tumour grade ($p=2.9x10^{-30}$)**			
Low	1,273/3,660	3.08 (2.81, 3.38)	1.00
Medium	8,549/21,380	3.85 (3.69, 4.02)	1.25
High	27,927/65,777	4.83 (4.68, 4.99)	1.57
**Breast cancer laterality ($p=3.5x10^{-02}$)**			
Left	20,235/47,916	4.09 (3.94, 4.25)	1.00
Right	17,515/42,901	3.98 (3.82, 4.15)	0.97
**Index of multiple deprivation ($p=1.2x10^{-27}$)**			
<20% (least deprived)	7,205/19,678	3.58 (3.41, 3.75)	1.00
20- 39%	7,731/19,562	3.85 (3.68, 4.03)	1.08
40- 59%	7,861/18,710	4.02 (3.86, 4.19)	1.12
60- 79%	7,508/16,996	4.33 (4.13, 4.55)	1.21
80+% (most deprived)	7,443/15,869	4.69 (4.48, 4.90)	1.31
**Region of residence ($p=3.4x10^{-20}$)**			
Eastern	3,722/9,312	3.90 (3.70, 4.11)	1.00
North West	5,200/12,153	4.35 (4.08, 4.64)	1.12
Northern & Yorkshire	5,920/14,122	4.72 (4.39, 5.09)	1.21
Oxford	2,216/5,449	4.37 (4.04, 4.72)	1.12
South West	4,959/12,789	4.19 (3.90, 4.50)	1.07
Thames	8,219/19,147	3.28 (3.03, 3.55)	0.84
Trent	2,864/7,264	4.37 (4.09, 4.68)	1.12
West Midlands	4,646/10,577	4.38 (4.16, 4.62)	1.12
**Time since diagnosis (years) ($p=1.2x10^{-09}$)**			
<=1	3,111/90,818	3.35 (3.13, 3.58)	1.00
1- 2	6,343/87,195	5.75 (5.39, 6.13)	1.72
2- 3	5,804/80,814	6.08 (5.72, 6.47)	1.82
3- 4	4,914/74,995	5.83 (5.47, 6.21)	1.74
4- 5	3,399/70,067	4.45 (4.19, 4.74)	1.33
5- 10	8,290/48,957	3.34 (3.17, 3.52)	1.00
10- 15	3,832/40,404	2.63 (2.40, 2.88)	0.79
15- 20	1,778/23,132	2.51 (2.06, 3.05)	0.75
20- 21	276/10,491	2.58 (1.92, 3.47)	0.77
**All ER- negative**	37,750/90,818	4.04 (3.89, 4.19)	

Figure S28: Adjusted annual all-cause mortality rates in women with early breast cancer with ER-positive or ER-negative disease according to nine characteristics, and time since diagnosis

For each characteristic, rates are adjusted for all the other characteristics shown including time since diagnosis.

All-cause mortality (to accompany figures 3-7)

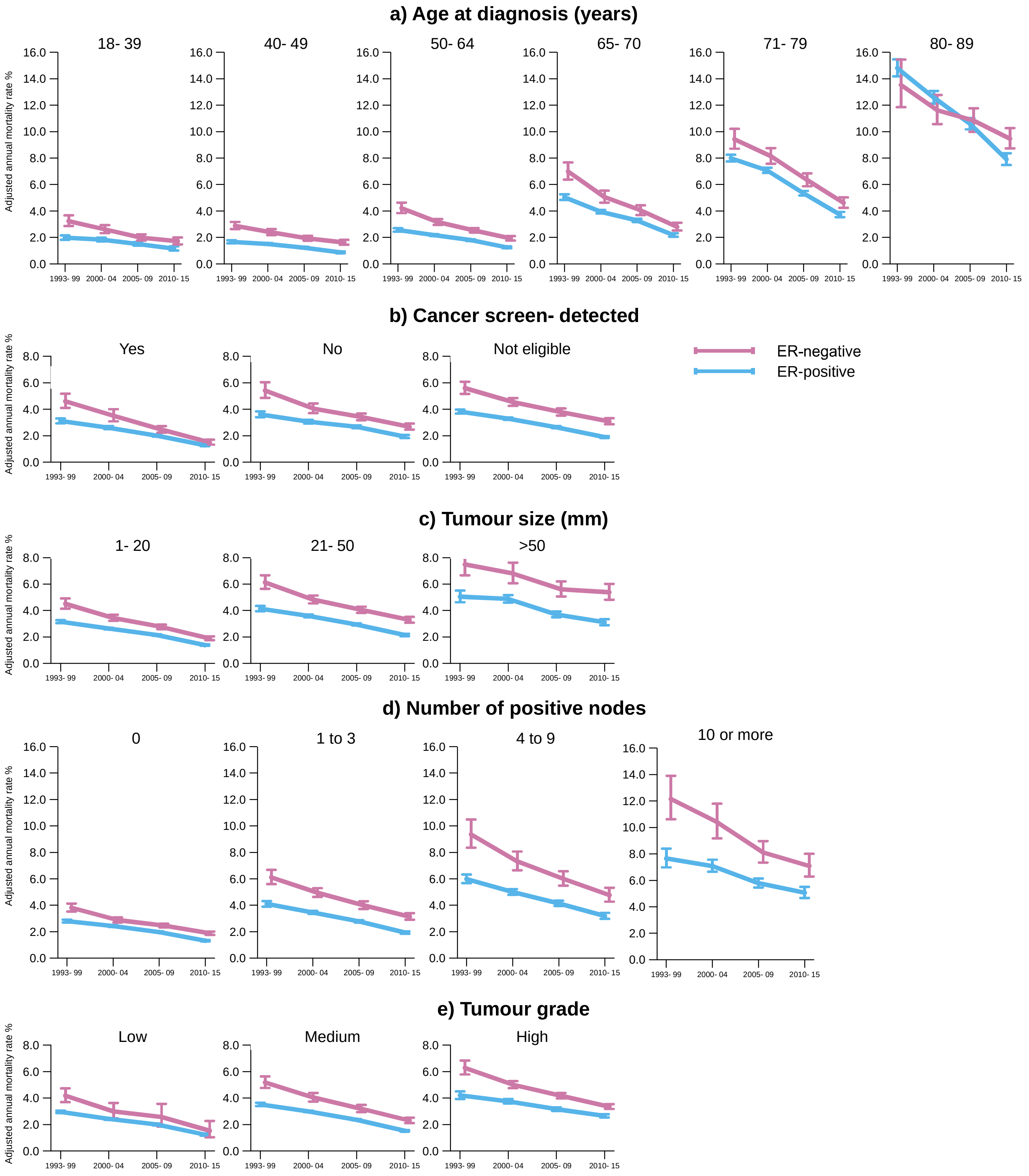

(Figure S29 continued on next page)

Figure S29 (continued)

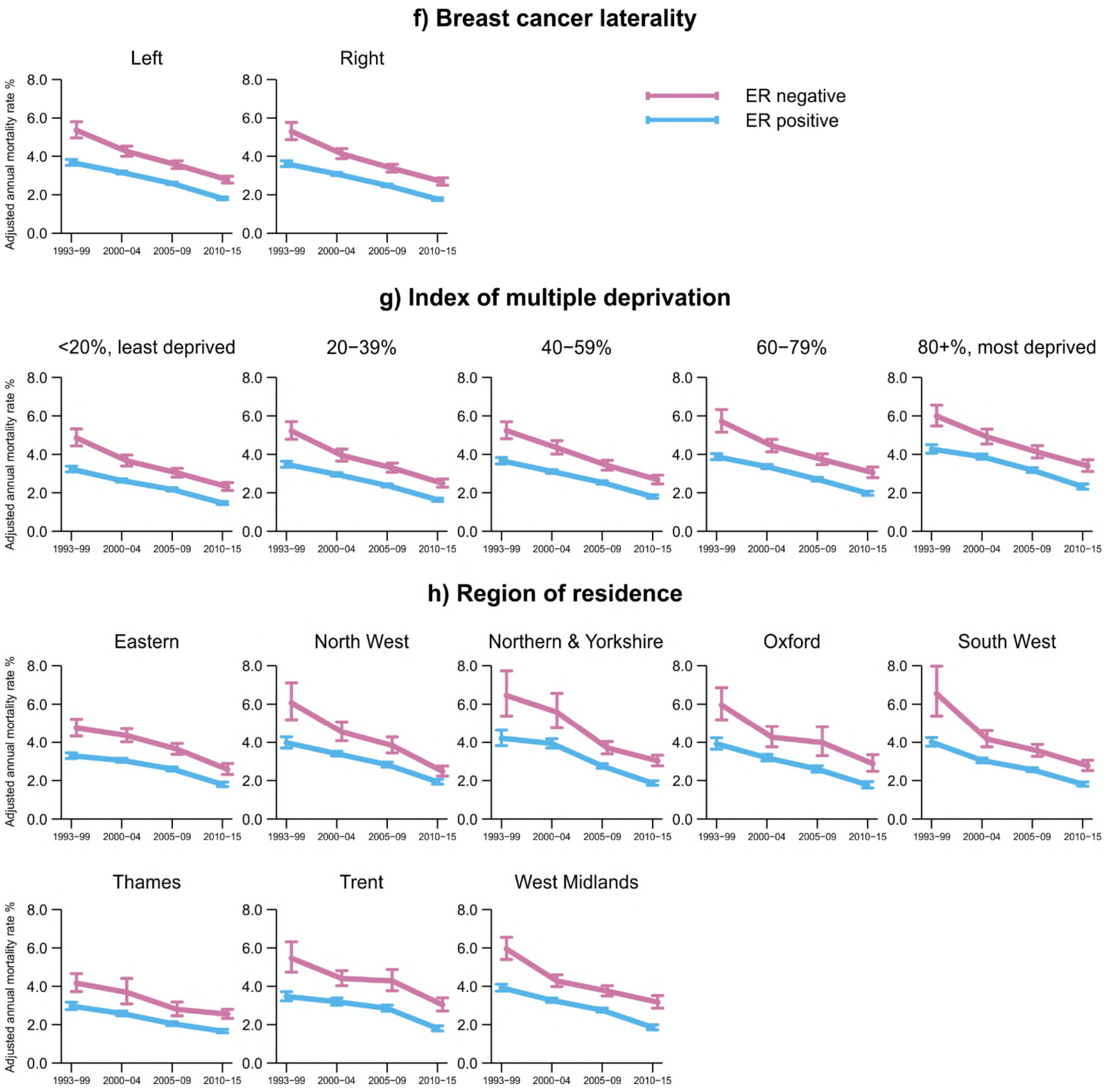

Figure S29: Adjusted annual all-cause mortality rates in women with early breast cancer with ER-positive or ER-negative disease by calendar period of diagnosis, according to various characteristics

For each characteristic, rates are adjusted for all the characteristics shown in figure 2 and also for time since diagnosis, breast cancer laterality, index of multiple deprivation and region of residence.

Vertical lines are 95% confidence intervals.

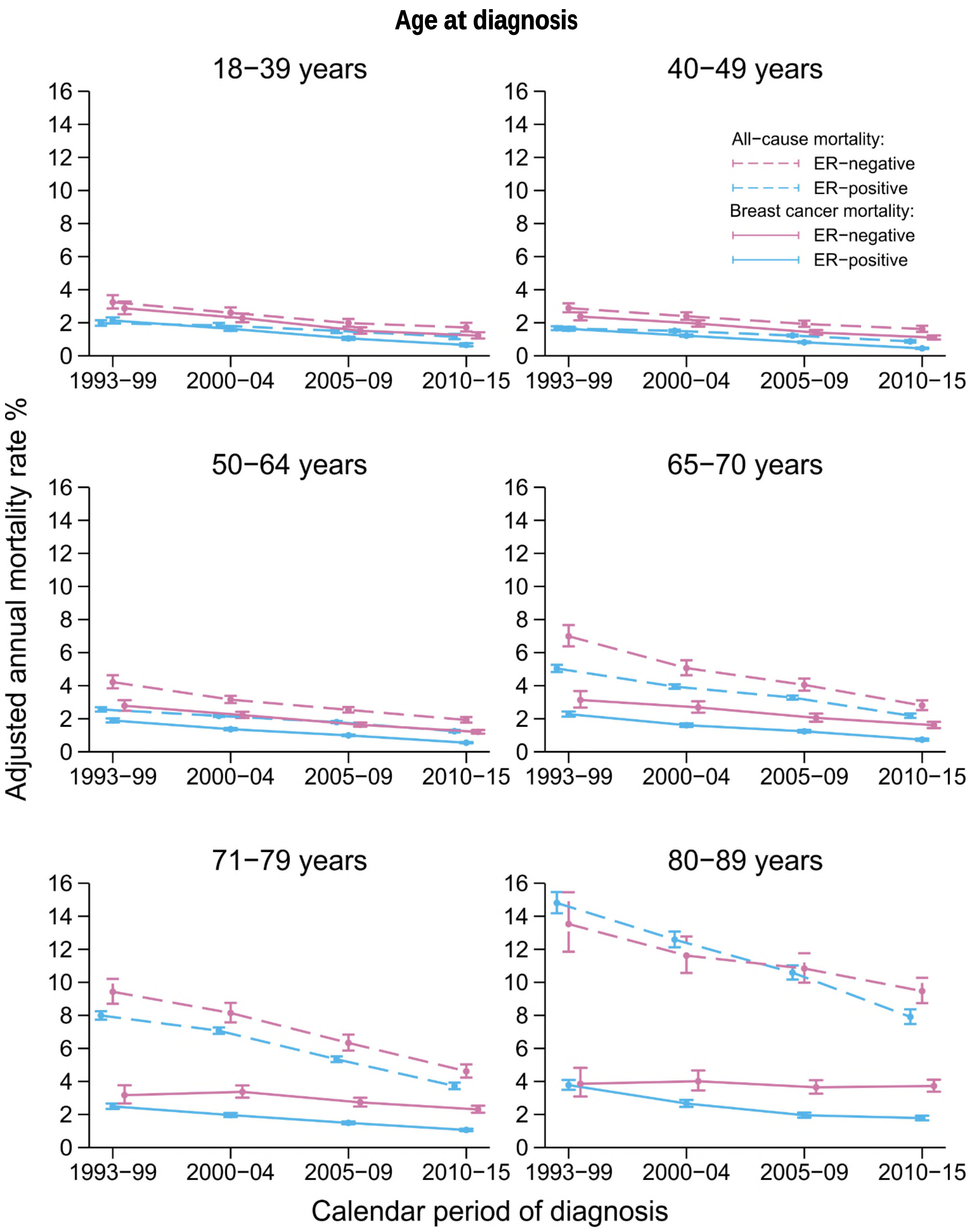

Figure S30: Adjusted annual all-cause mortality rates (dashed lines) and breast cancer mortality rates (solid lines) in women with early breast cancer with ER-positive or ER-negative disease by calendar period of diagnosis, according to age at diagnosis

Rates are adjusted for all the characteristics shown in figure 2 and also for time since diagnosis, breast cancer laterality, index of multiple deprivation and region of residence.
Vertical lines are 95% confidence intervals

All-cause mortality (to accompany figure 8)

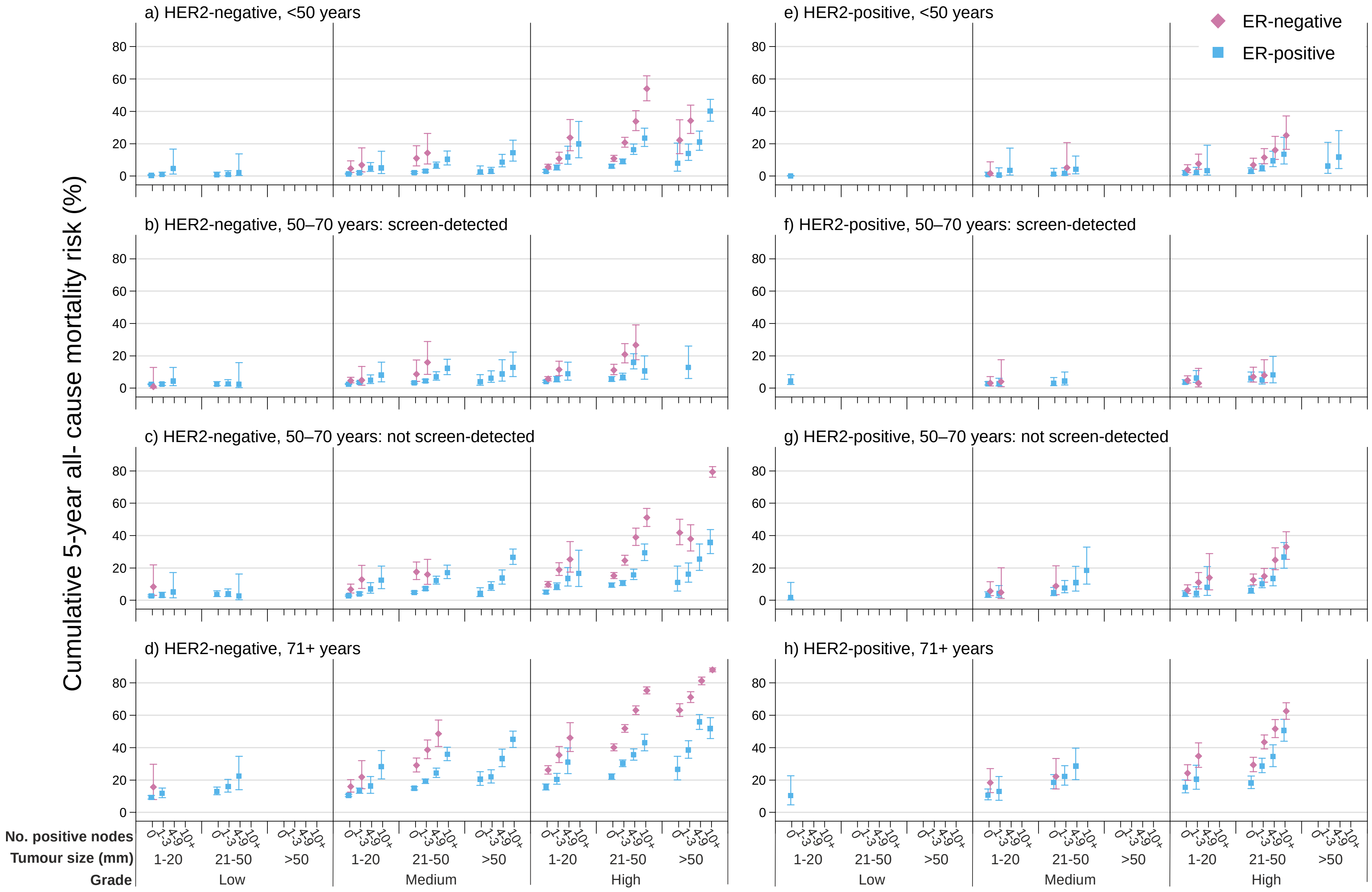

Figure S31: Cumulative five-year all-cause mortality risks in 156,338 women with early breast cancer diagnosed during 2010-2015 by categories of tumour grade, size and number of positive nodes in women with ER-positive or ER-negative disease. Figures are split by HER2 status, age and screening status

Vertical lines are 95% confidence intervals. Points are plotted for groups of women with data on at least 40 women and include 153,006/156,338 (97.9%) of the women.

Comparable Studies

Table S11: Other studies of breast cancer specific mortality in populations of patients with breast cancer or of relative mortality in populations of patients with breast cancer compared with the general population

We searched Medline for papers published between January 2000 and August 2021 that reported breast cancer specific mortality in populations of patients with breast cancer or relative mortality in populations of patients with breast cancer compared with the general population. We used search terms: "breast cancer or breast neoplasm" and "survival or mortality". We excluded studies with less than 5,000 women because these would lack power for assessing relationships between patient or tumour characteristics and breast cancer mortality. Studies reporting results from a single centre were also excluded, as were studies where all women were diagnosed before 1990. The search found 2126 publications of which 16 reported findings that were comparable with our study. Ten studies reported breast cancer mortality in women with early invasive breast cancer (section a below). Six studies reported only combined results for women with early and metastatic cancer (section b) so they are less comparable with our analyses. Studies are ordered according to the number of women with breast cancer.

Most studies only considered a few specific aspects of breast cancer mortality and none considered as wide a range of characteristics as our study. Where results reported were comparable with those in our study they were, without exception, consistent with our findings

Reference	Geographical region	Type of study	Inclusion criteria and follow-up	Number of women with breast cancer	Results (relevant table or figure in study)	Comparable results in present study (relevant table or figure in present study)
**(a) Studies that reported outcomes in early invasive breast cancer**						
Coleman 2011[1]	International	Population-based Registries from Australia, Canada, Denmark, Norway, Sweden, and the UK	Adults (aged 15-99 years) diagnosed with invasive primary malignancy of colorectum, breast, lung, or ovary during 1995-2007, with follow-up to 31 Dec 2007	833,350 women with early invasive breast cancer	Increasing age-standardised 5-year survival for women diagnosed in 1995-99, 2000-02, 2005-07 in all countries (Figure 2)	Decreasing breast cancer mortality with increasing calendar year of diagnosis. (Figure 2)
Sopik 2018[2]	United States	Population-based SEER Cancer Registries	Women diagnosed with invasive breast cancer as first cancer between 1990 and 2014, followed to 31 Dec 2014.	735,781 early invasive breast cancer (excluding distant metastases)	Positive association between tumour size and 15-year breast cancer mortality (Supplementary figure 7)	Higher adjusted annual breast cancer mortality in women with larger tumours. (Figure 2)
Holleczek[3] 2012	United States<br>Germany	Population-based SEER 9 registries<br><br>Saarland Registry	Women diagnosed with invasive breast cancer between 1988 and 2008, followed to 31 Dec 2008	United States: 275,555 with localized cancer, 132,354 with regional cancer<br><br>Germany: 5856 with localized cancer, 5502 with regional cancer<br><br>Total: 419,267	5-year age-standardized relative survival (%) by diagnosis year<br>United States: Localized breast cancer<br>1993-96 – 95.7<br>1997-00 – 96.3<br>2001-04 – 97.3<br>2005-08 – 97.3<br>United States: Regional breast cancer<br>1993-96 – 75.1<br>1997-00 – 77.5<br>2001-04 – 81.7<br>2005-08 – 82.8<br>Germany: Localized breast cancer<br>1993-96 – 91.4<br>1997-00 – 94.5<br>2001-04 – 97.4<br>2005-08 – 98.7<br>Germany: Regional breast cancer<br>1993-96 – 64.0<br>1997-00 – 70.3<br>2001-04 – 74.8<br>2005-08 – 78.6<br>(Table 3)	Decreasing breast cancer mortality with increasing calendar year of diagnosis irrespective of prognostic score. (Figures 5,6)

(continued on next page)

Reference	Geographical region	Type of study	Inclusion criteria and follow-up	Number of women with breast cancer	Results (relevant table or figure in study)	Comparable results in present study (relevant table or figure in present study)
Van der Meer 2021[4]	Netherlands	Population-based Netherlands cancer registry	Women diagnosed with invasive breast cancer between 1989 and 2017, followed to Jan 31, 2018	300,417 with Stages I,II and III breast cancer	5-year relative survival (%) by year of diagnosis<br>Stage I<br>1989–1999: 91.4<br>2000–2009: 97.5<br>2010–2016: 99.2<br><br>Stage II<br>1989–1999: 75.5<br>2000–2009: 89.0<br>2010–2016: 93.1<br><br>Stage III<br>1989–1999: 50.1<br>2000–2009: 67.6<br>2010–2016: 78.6<br>(Figure 5b, Table S6)	Increasing breast cancer survival with increasing calendar year of diagnosis, irrespective of prognostic score (Figures 5,6)
Ai 2020[5]	United States	Population based SEER database	Women with primary, operable, invasive breast cancer, diagnosed between Jan 1998 and Dec 2015	142,808 with early invasive breast cancer	Adjusted hazard ratios for breast-cancer specific mortality:<br>Age in years:<br>≤50: 1.00<br>51-60: 1.03<br>61-70: 1.20<br>≥70: 1.85<br><br>Grade:<br>1: 1.00<br>2: 1.78<br>3: 2.66<br><br>ER status:<br>Positive: 1.00<br>Negative:1.30<br>(Table 2)	Similar patterns for these characteristics (Figure 2)
EBCTCG 2017[6]	International	Women in randomised trials	ER-positive early invasive breast cancer, randomised between 1976 and 2011 to receive 5 years endocrine therapy. Length of follow-up and other inclusion criteria variable.<br><br>Excluded:<br>Women aged ≥75 years<br>Tumours >5cm<br>10+ involved nodes	74,194 women with early invasive breast cancer in 78 trials	20-year cumulative risk of breast cancer death:<br>0 positive nodes: 15%<br>1-3 positive lymph nodes: 28%<br>4-9 positive lymph nodes: 49%<br>(Figure 2B)<br><br>Breast cancer mortality increased according to increasing tumour size, number of nodes involved and grade (Figure S17)	Comparable 20-year cumulative risk of breast cancer death for all women with ER-positive cancer: 33.5%.<br>(Figure 1 bottom panel, Table S6)<br><br>Similar variation seen with these characteristics. (Figure 2)

(continued on next page)

Reference	Geographical region	Type of study	Inclusion criteria and follow-up	Number of women with breast cancer	Results (relevant table or figure in study)	Comparable results in present study (relevant table or figure in present study)
Nordenskjold 2019[7]	Sweden	Population-based Southeastern and western healthcare regions	Women diagnosed with invasive breast cancer between 1989 and 2013, followed to 31 Dec 2014	38,747 women with stage I, II or III breast cancer	10-year excess mortality ratio relative to first category listed by calendar year of diagnosis:<br>Stage I<br>1989–1993: 1.00<br>1994–1998: 0.88<br>1999–2003: 0.72<br>2004–2008: 0.55<br><br>Stage II N0<br>1989–1993 1.00<br>1994–1998: 1.01<br>1999–2003: 0.75<br>2004–2008: 0.70<br><br>Stage II N+<br>1989–1993: 1.00<br>1994–1998: 0.95<br>1999–2003: 0.68<br>2004–2008: 0.56<br><br>Stage III<br>1989–1993: 1.00<br>1994–1998: 0.96<br>1999–2003: 0.74<br>2004–2008: 0.64<br>(Table 4)	Comparable decreasing breast cancer mortality with increasing calendar year of diagnosis irrespective of prognostic score. (Figures 5,6)
					For each operable stage of cancer, patients aged below 40 years or more than 70 years when diagnosed tended to have lower survival than patients diagnosed at 40–69 years of age. (Abstract)	Adjusted breast cancer mortality was higher in women diagnosed when aged 71-79 years or <40 years than at other age groups. (Figure 2).
					Survival significantly increased over time for patients diagnosed at ages below 40, 40–54 and 54–69, but there were less marked increases for patients older than 70 when diagnosed. (Abstract)	Decrease in mortality with calendar year of diagnosis less marked for women aged 71-79 at diagnosis than for younger women. (Figure 3)
Guo, 2018[8]	United States	Population-based SEER 9 registries	Women diagnosed with breast cancer between 1975 and 2015 at ages 20-39, followed to 31 Dec 2015	17,718 with localized breast cancer	5-year breast cancer survival (%) by year of diagnosis:<br>Localized breast cancer<br>1975-1979 – 85.4<br>1980-1984 – 86.2<br>1985-1989 – 89.1<br>1990-1994 – 90.8<br>1995-1999 – 93.2<br>2000-2004 – 94.8<br>2005-2009 – 96.1<br>2010-2015 – 95.4	Decreasing breast cancer mortality with increasing calendar year of diagnosis, irrespective of prognostic score. (Figures 5,6)

(continued on next page)

Reference	Geographical region	Type of study	Inclusion criteria and follow-up	Number of women with breast cancer	Results (relevant table or figure in study)	Comparable results in present study (relevant table or figure in present study)
Guo 2018[8] (*Continued)*				15,520 with regional breast cancer<br><br>Total: 33,238	Regional breast cancer<br>1975-1979 – 63.8<br>1980-1984 – 64.8<br>1985-1989 – 65.7<br>1990–1994 –72.1<br>1995-1999 – 77.3<br>2000-2004 – 83.1<br>2005-2009 – 86.5<br>2010-2015 – 85.6<br>(Table 1)	
Rose, 2015[9]	United States	Population-based SEER 17 database	Women between 18 and 70, diagnosed with node-positive, non-metastatic breast cancer between 1 Jan 1990 and 31 Dec 2003. Median follow-up 109 months	8719 with node-positive invasive breast cancer	Adjusted hazard ratios for breast-cancer specific mortality relative to first category:<br>Age in years:<br>≤45: 1.0<br>46-55: 0.8<br>56-65: 0.9<br>≥66: 1.3<br><br>Grade:<br>1: 1.0<br>2: 2.0<br>3: 2.8<br><br>ER status:<br>Positive: 1.0<br>Negative:1.1<br><br>Number of involved nodes:<br>1: 1.0<br>2-3: 1.3<br>>4: 2.0<br>(Table 3)	Similar patterns for these characteristics (Figure 2)
Van Maaren 2018[10]	Netherlands	Netherlands cancer registry	Women registered with first invasive breast cancer stages T1-2, N0-1 during 2005 and treated with surgery, followed to 1 Feb 2017.<br><br>Excluded: Unknown ER, PR or HER2 status	7969 with early invasive breast cancer	Triple negative patients had lower relative overall survival than patients in other groups.<br>(Figure 5)	Higher cumulative breast cancer mortality in ER-negative than ER-positive women.<br>(Figure 1 bottom panel)

(continued on next page)

Reference	Geographical region	Type of study	Inclusion criteria and follow-up	Number of women with breast cancer	Results (relevant table or figure in study)	Comparable results in present study (relevant table or figure in present study)
**(b) Studies that reported only combined results for women with early and metastatic cancer**						
Hill, 2019[11]	United States	Population-based SEER, 18 registries	Women diagnosed with first invasive breast cancer between 1975 and 2010, followed to 31 Dec 2014	930, 910 (includes unknown number with metastatic cancer)	Five-year breast cancer survival probability increased with diagnosis year for women with ER-positive and ER-negative cancer (Figures 2 and 3)	Decreasing breast cancer mortality with increasing calendar year of diagnosis in both ER-positive and ER-negative cancer (Figure 2)
Du 2008[12]	United States	Population-based SEER 9 registries	Women diagnosed with breast cancer between 1975 and 2003, followed to 31 Dec 2003.	316,149 (includes 20,967 with metastatic cancer)	Breast cancer mortality in specific time-since-diagnosis categories decreased with calendar year of diagnosis (Table 1)	Breast cancer mortality decreased with calendar period of diagnosis. (Figure 2)
Johnson 2019[13]	United States	Population-based SEER registries	Women diagnosed with invasive breast cancer as their first cancer between 1 Jan 2000 and 31 Dec 2015, followed to 31 Dec 2015.	206,332 (includes 8491 with metastatic cancer)	Adjusted breast cancer mortality was lowest for women aged around 45 years at diagnosis. Rates for women diagnosed at younger and older ages were higher. (Figure 3)	Adjusted breast cancer mortality rates were lowest for women aged 40-49 years at diagnosis and higher for women diagnosed at younger and older ages. (Figure 2)
Jatoi 2007[14]	United States	Population-based SEER 9 registries	Women diagnosed with invasive breast cancer between 1990 and 2003, followed to 31 Dec 2003	147,289 with ER-positive cancer<br><br>43,544 with ER-negative cancer	Over the study period, breast-cancer mortality rates decreased by 4.23% per year among women with ER-positive tumours and 2.12% per year among women with ER-negative tumours. (Figure 3A)	Decreases in breast cancer mortality with calendar period of diagnosis. (Figure 2)
				(Includes 10,172 with metastatic cancer)<br><br>Total: 190,833	Over the study period, breast cancer mortality rates among women ≥70 years decreased 14% for ER-positive tumours, with no decline for ER-negative tumours (Figure 3C&D)	Decrease in mortality with calendar year of diagnosis less marked for women aged 71-79 with ER-negative cancer than for other women. (Figure 3)
					ER-negative breast-cancer mortality rates increased to a sharp peak of 7-8% per year 2 years after initial breast cancer diagnosis, and then declined. In contrast, ER-positive hazard rates lacked a sharp peak but had a stable long-term rate of 1% to 2% per year. (Figure 2)	Difference between ER-positive and ER-negative cancer in patterns of annual breast cancer mortality. (Figure 1 bottom panel)
Yu 2009[15]	United States	Population-based SEER 13 registries	Women aged 15 years or older diagnosed with first invasive breast cancer between 1 Jan 1998 and 31 Dec 2002, followed to 31 Dec 2005	112, 543 (includes 4770 with metastatic cancer)	Hazard ratio for breast cancer mortality by socioeconomic status adjusted for age, calendar year, and stage:<br>Highest: 1.00<br>Upper middle: 1.00<br>Lower middle: 1.08<br>Lowest: 1.10<br>(Table 2)	Adjusted breast cancer mortality increased according to index of multiple deprivation (Figure S10)
Australian Institute of Health and Welfare 2013[16]	Australia	Population-based Australian Cancer Database	People diagnosed with breast cancer between 1982 and 2010, followed to 31 Dec 2010 (Analyses also performed for other cancer types)	Not stated All patients diagnosed with invasive cancer (metastatic cancer not excluded)	Five year survival increased from 72% for women diagnosed in 1982-1987 to 89% for women diagnosed in 2006-2010. (Figure 4)<br><br>Relative survival was lower for women in the lowest socioeconomic quintile compared with the other quintiles. (Figure 6)	Breast cancer mortality decreases with calendar period of diagnosis. (Figure 2)<br><br>Breast cancer mortality higher in more deprived groups. (Figure S10)

Abbreviations: ER oestrogen receptor; HER human epidermal growth factor receptor

References for Table S11

1 Coleman MP, Forman D, Bryant H, et al. Cancer survival in Australia, Canada, Denmark, Norway, Sweden, and the UK, 1995-2007 (the International Cancer Benchmarking Partnership): an analysis of population-based cancer registry data. *Lancet* 2011; **377**: 127-38.

2 Sopik V, Narod SA. The relationship between tumour size, nodal status and distant metastases: on the origins of breast cancer. *Breast Cancer Res Treat* 2018; **170**: 647-56.

3 Holleczek B, Brenner H. Trends of population-based breast cancer survival in Germany and the US: decreasing discrepancies, but persistent survival gap of elderly patients in Germany. *BMC Cancer*. 2012; **12**: 317.

4 Van der Meer DJ, Kramer I, Van Maaren MC, et al. Comprehensive trends in incidence, treatment, survival and mortality of first primary invasive breast cancer stratified by age, stage and receptor subtype in the Netherlands between 1989 and 2017. *Int J Cancer* 2021; **148**: 2289-303.

5 Ai B, Wang X, Kong X, Wang Z, Fang Y, Wang J. Conditional survival of female patients with operable invasive breast cancer in US: A population-based study. *J Cancer* 2020; **11**: 5782-91.

6 Early Breast Cancer Trialists' Collaborative Group. 20-Year Risks of Breast-Cancer Recurrence after Stopping Endocrine Therapy at 5 Years. *N Engl J Med* 2017; **377**: 1836-46.

7 Nordenskjold AE, Fohlin H, Arnesson LG, et al. Breast cancer survival trends in different stages and age groups - a population-based study 1989-2013. *Acta Oncol* 2019; **58**: 45-51.

8 Guo F, Kuo YF, Shih YCT, Giordano SH, Berenson AB. Trends in breast cancer mortality by stage at diagnosis among young women in the United States. *Cancer* 2018; **124**: 3500-9.

9 Rose BS, Jiang W, Punglia RS. Effect of lymph node metastasis size on breast cancer-specific and overall survival in women with node-positive breast cancer. *Breast Cancer Res Treat* 2015; **152**: 209-16.

10 Van Maaren MC, Strobbe LJA, Smidt ML, Moossdorff M, Poortmans PMP, Siesling S. Ten-year conditional recurrence risks and overall and relative survival for breast cancer patients in the Netherlands: Taking account of event-free years. *Eur J Cancer* 2018; **102**: 82-94.

11 Hill DA, Prossnitz ER, Royce M, Nibbe A. Temporal trends in breast cancer survival by race and ethnicity: A population-based cohort study. *PLoS One* 2019; **14**: e0224064.

12 Du XL, Fox EE, Lai D. Competing causes of death for women with breast cancer and change over time from 1975 to 2003. *Am J Clin Oncol* 2008; **31**: 105-16.

13 Johnson HM, Irish W, Muzaffar M, Vohra NA, Wong JH. Quantifying the relationship between age at diagnosis and breast cancer-specific mortality. *Breast Cancer Res Treat* 2019; **177**: 713-22.

14 Jatoi I, Chen BE, Anderson WF, Rosenberg PS. Breast Cancer Mortality Trends in the United States According to Estrogen Receptor Status and Age at Diagnosis. *J Clin Oncol* 2007; **25**: 1683-90.

15 Yu XQ. Socioeconomic disparities in breast cancer survival: relation to stage at diagnosis, treatment and race. *BMC Cancer* 2009; **9**: 364.

16 Australian Institute of Health and Welfare. Cancer survival and prevalence in Australia: Period estimates from 1982 to 2010. *Asia Pac J Clin Oncol* 2013; **9**: 29-39.
